# *ZmCTLP1* is required for the maintenance of lipid homeostasis and the basal endosperm transfer layer in maize kernels

**DOI:** 10.1111/nph.17754

**Published:** 2021-10-11

**Authors:** Mingjian Hu, Haiming Zhao, Bo Yang, Shuang Yang, Haihong Liu, He Tian, Guanghou Shui, Zongliang Chen, Lizhu E, Jinsheng Lai, Weibin Song

**Affiliations:** ^1^ State Key Laboratory of Plant Physiology and Biochemistry and National Maize Improvement Center Department of Plant Genetics and Breeding China Agricultural University Beijing 100193 China; ^2^ State Key Laboratory of Plant Physiology and Biochemistry College of Biological Sciences China Agricultural University Beijing 100193 China; ^3^ State Key Laboratory of Molecular Developmental Biology Institute of Genetics and Developmental Biology Chinese Academy of Sciences Beijing 100101 China; ^4^ Waksman Institute of Microbiology Rutgers University Piscataway NJ 08854‐8020 USA; ^5^ Center for Crop Functional Genomics and Molecular Breeding China Agricultural University Beijing 100193 China

**Keywords:** choline transporter, kernel development, maize, plasmodesmata, *ZmCTLP1*

## Abstract

- Maize kernel weight is influenced by the unloading of nutrients from the maternal placenta and their passage through the transfer tissue of the basal endosperm transfer layer (BETL) and the basal intermediate zone (BIZ) to the upper part of the endosperm.
- Here, we show that *Small kernel 10* (*Smk10*) encodes a choline transporter‐like protein 1 (ZmCTLP1) that facilitates choline uptake and is located in the *trans*‐Golgi network (TGN). Its loss of function results in reduced choline content, leading to smaller kernels with a lower starch content.
- Mutation of *ZmCTLP1* disrupts membrane lipid homeostasis and the normal development of wall in‐growths. Expression levels of *Mn1* and *ZmSWEET4c*, two kernel filling‐related genes, are downregulated in the *smk10*, which is likely to be one of the major causes of incompletely differentiated transfer cells. Mutation of *ZmCTLP1* also reduces the number of plasmodesmata (PD) in transfer cells, indicating that the *smk10* mutant is impaired in PD formation. Intriguingly, we also observed premature cell death in the BETL and BIZ of the *smk10* mutant.
- Together, our results suggest that ZmCTLP1‐mediated choline transport affects kernel development, highlighting its important role in lipid homeostasis, wall in‐growth formation and PD development in transfer cells.

## Introduction

Kernel weight, a key determinant of grain yield, depends on the effective transport of carbohydrates from the maternal phloem to the filial tissues (Gui *et al*., 2014; Sosso *et al*., 2015). Nutrients pass through four cell types from the maternal tissues to the central endosperm: the pedicel (PED), the placento‐chalazal region (PC), the basal endosperm transfer layer (BETL), and the basal intermediate zone (BIZ) (Porter *et al*., 1987; Lalonde *et al*., 2004; Royo *et al*., 2007; Zhang *et al*., 2007; Becraft & Gutierrez‐Marcos, 2012; Bihmidine *et al*., 2013; Leroux *et al*., 2014; Larkins, 2019; Song *et al*., 2019). Assimilates are unloaded through maternal vascular terminals in the PED and pass symplastically and apoplastically into the PC, then apoplastically through the BETL into the central endosperm (Muhitch, 1989; Miller & Chourey, 1992; Santandrea *et al*., 2002). The PED and PC are two key maternal tissues that control the supply of nutrients to filial tissues (Muhitch, 1993; Santandrea *et al*., 2002; Kladnik *et al*., 2004).

Adjacent to the PC are the BETL and BIZ, two elongated cell types located at the bottom of the endosperm (Shannon, 1972; Becraft & Gutierrez‐Marcos, 2012; Leroux *et al*., 2014; Larkins, 2019). The BETL, a unique endosperm cell layer with wall in‐growths that increase the surface area of the plasma membrane and cell wall, plays a major part in the transfer of nutrient solutes from the maternal placenta to the developing endosperm (Davis *et al*., 1990; Wang *et al*., 1994). Several genes have been reported to influence nutrient uptake and allocation in this region, thereby regulating kernel development. Mn1 (Miniature1) is located in the BETL and the PED and catalyses the cleavage of sucrose into hexoses to generate a physiological gradient of photosynthate that passes symplastically and apoplastically into the PC, then apoplastically through the BETL to the endosperm (Miller & Chourey, 1992). Loss of *Mn1* function results in smaller kernel size (Miller & Chourey, 1992; Cheng *et al*., 1996; Cheng & Chourey, 1999; Kang *et al*., 2009; Sosso *et al*., 2015), whereas *Mn1* overexpression improves kernel filling and markedly increases kernel size and weight (Wang *et al*., 2008; Li *et al*., 2013). Its rice homologue *GIF1* (*Grain Incomplete Filling 1*) is also recognised as a positive regulator of seed filling (Wang *et al*., 2008).

A second gene, *ZmSWEET4c* (*Sugars Will Eventually be Exported Transporter 4c*), encodes a sugar transporter reported to transport hexoses produced by Mn1 to the endosperm (Sosso *et al*., 2015). Its soybean homologue *GmSWEET10* regulates oil content and seed size by transporting sucrose and hexose from the seed coat to the developing embryo (Wang *et al*., 2020). The development of fully differentiated BETL with wall in‐growths requires the normal functions of *Mn1* and *ZmSWEET4c* (Kang *et al*., 2009; Sosso *et al*., 2015). Wall in‐growth formation enlarges the surface area of the plasma membrane and therefore requires abundant lipid precursors such as phosphatidic acid, triglyceride, acetyl‐CoA and choline (van Greevenbroek *et al*., 1995; Michel *et al*., 2006; Correa *et al*., 2020).

Choline is a quaternary amine that plays a fundamental role in cell membrane phospholipid metabolism and the synthesis of the neurotransmitters acetylcholine and betaine (Michel *et al*., 2006). Disorders of choline uptake, transport and metabolism affect cell proliferation and differentiation in animals, thereby contributing to neurodegenerative disorders such as Alzheimer’s disease and Parkinson’s disease (Michel *et al*., 2006; Ueland, 2011). Choline transport is mediated by three different systems: high‐affinity choline transporters (CHT1s), intermediate‐affinity choline transporter‐like (CTL) proteins, and low‐affinity polyspecific organic cation transporters (OCTs) (Michel *et al*., 2006). *CTL1* is widely expressed in animal tissues, and its encoded protein specifically regulates choline supply for phospholipid and sphingolipid synthesis (Machová *et al*., 2009; Wang *et al*., 2017). In plants, the first choline transporter AtCTL1 was isolated in *Arabidopsis thaliana*, and it has been shown to have choline transport activity (Dettmer *et al*., 2014). *AtCTL1* also regulates auxin distribution by promoting the trafficking of auxin efflux transporters during plant growth and development (Wang *et al*., 2017). *AtCTL1* is essential for the formation and development of PD and plays vital roles in the formation of sieve tubes; mutation of *AtCTL1* alters ion homeostasis in rosette leaves from one 5‐wk‐old plant, ultimately causing a severely stunted growth phenotype (Dettmer *et al*., 2014; Gao *et al*., 2017; Kraner *et al*., 2017; Wang *et al*., 2017). Nonetheless, information on the choline transporter in plants is very limited, and its roles in kernel development and kernel biology remain largely unknown.

In this study, we identified the maize CTL1 choline transporter homologue ZmCTLP1 through map‐based cloning of *smk10*. Mutation of *ZmCTLP1* disrupts kernel development, dramatically decreasing kernel size and weight. In *smk10*, the nutrient transfer cells of the BETL and BIZ exhibit typical features of dysfunction, including irregular cell shape, decreased PD number, premature cell death and impaired lipid homeostasis. RNA‐seq and immunoblot analysis revealed that the mutant shows altered expression of many genes involved in nutrient metabolism and transport. Collectively, these findings demonstrate that *ZmCTLP1* is required for kernel development and participates in lipid homeostasis, wall in‐growth formation and PD development in transfer tissues.

## Materials and Methods

### Plant materials

The ethyl methanesulfonate (EMS)‐generated *smk10* mutant was obtained from mutant populations produced in our laboratory on the B73 genetic background. The *smk10* mutant was crossed with Mo17 and then self‐pollinated to generate F2 and F3 populations. The F3 population was used for fine mapping of *smk10*. The wild‐type and the *smk10* were used for cytological and biochemical analyses. Leaves and roots were collected from at least three 10‐d‐old B73 seedlings. Leaf, root, internode, node, silk, cob and husk tissues were collected at the R1 stage 70 d after sowing. Immature B73 kernels were harvested at 2, 4, 6, 8, 10, 12, 14, 16 and 20 d after pollination (DAP). Maize plants were grown under natural conditions at the Shang‐Zhuang experimental field of China Agricultural University in Beijing.

### Light microscopy, fluorescence microscopy and transmission electron microscopy

For light microscopy, 4–12 DAP wild‐type and *smk10* kernels were harvested. Paraffin sections were prepared using the methods described by Li *et al*. (2018). The sections were stained with fuchsin or eosin and observed using an Olympus SZ51 microscope. Detection of PD after aniline blue staining (0.1% aniline blue in double‐distilled water and 1 M glycine, pH 9.5, at a volume ratio of 2 : 3) was performed using a ZEISS LSM710 confocal microscope (Germany) with scan speed set at ‘Best signal’. The excitation wavelength for PD signal was 440 nm, and the emission wavelengths were 450–511 nm.

For transmission electron microscopy observation, 12 DAP immature kernels from the wild‐type and *smk10* were cut along the horizontal axis for resin section preparation. Each sample was fixed with glutaraldehyde and osmic acid and then embedded with low viscosity resin (Electron Microscopy Sciences, cat. #14300). Sections were stained with lead citrate and observed using a Hitachi H7600 transmission electron microscope (Japan).

### Determination of soluble sugar, starch, protein, choline and lipid contents

#### Soluble sugars, starch and protein

Mature kernels (15 g) were pulverised and extracted with water. The samples were dispersed with an ultrasonic dispersion instrument for 30 min and then measured using a Chromeleon chromatography management system (Dionex, Sunnyvale, CA, USA). Starch and protein contents were measured as previously described (Clegg, 1956; Wang *et al*., 2011).

#### Choline and lipids

Samples were prepared and measured using the methods described by Kraner *et al*. (2017). In brief, *c*. 30 mg of maize kernel tissue were frozen in liquid N_2_ and ground to a fine powder in a cold mortar. Metabolites were extracted using a MeOH : CHCl_3_ : H_2_O (6 : 2 : 2) extraction buffer. MS analysis for the quantification of choline was performed using an Ultimate 3000 UHPLC system coupled to a Q‐Exactive MS instrument (Thermo Scientific, Bremen, Germany). Lipid contents were determined according to a previously described protocol (Shui *et al*., 2010; Gao *et al*., 2017). In brief, the sample (100 mg) was combined with 900 μl of chloroform : methanol (1 : 2) extraction buffer and acid‐washed glass beads (Sigma), vortexed for 10 min, and incubated overnight at 4°C with shaking at 1100 rpm. Next, 600 μl of chloroform : H_2_O (1 : 1) was added, and the sample was vortexed for 30 s. The mixtures were centrifuged at 7500 ***g*** for 5 min, and the lower organic phase was collected and dried with a Savant SpeedVac vacuum system (Thermo Fisher Scientific, Milford, MA, USA). The samples were stored at −80°C before mass spectrometric analysis.

### Map‐based cloning of *smk10*

To clone *smk10*, an F2 population was produced by crossing *smk10* with Mo17 and then self‐pollinating their F1 progeny. Thirty‐five individual miniature kernels were pooled, and 65 genetic markers were used for genotyping. The causal locus was mapped to a 2.3‐Mb genomic region on chromosome 2. To fine map the candidate gene, 3264 F3 individuals were yielded from the cross of *smk10* and Mo17. Five genetic markers within the 2.3‐Mb genomic region further narrowed the *smk10*‐containing region to 300 kb between markers M096 and M127. The candidate genes within this region were sequenced.

### Maize transformation

To obtain *ZmCTLP1* CRISPR lines, a 20‐bp sgRNA‐specific target site in the first exon of *ZmCTLP1* was cloned into the backbone of the pCAMBIA3301 vector and transformed into maize by *Agrobacterium*‐mediated transformation as previously described (Zhu *et al*., 2016). Transgenic plants were identified by PCR, and the targeted fragments were sequenced to detect variable sites. Two independent Cas9‐edited knockout lines (*cas‐1* and *cas‐2*) were obtained.

### Expression in *Xenopus* oocytes and choline uptake assays

#### Isolation and RNA injection of *Xenopus* oocytes

Coding sequences of *ZmCTLP1* and *AtCTL1* were codon optimised and synthesised. These cDNAs were cloned into the pT7TS expression vector. After linearisation of pT7TS plasmids with *Bam*HI, RNA was transcribed *in vitro* using an mRNA synthesis kit (mMESSAGE mMACHINE T7 kit, Ambion). *Xenopus laevis* oocytes were isolated in 25 ml ND96 (Ca^2+^ free) solution containing 43 mg collagenase and 12.5 mg trypsin inhibitor for 1.5 h and were then recovered in ND96 for 24 h. Oocytes were injected with cRNA (25 ng in 25 nl) after recovery and were incubated at 18°C in MBS solution (88 mM NaCl, 1 mM KCl, 2.4 mM NaHCO_3_, 0.71 mM CaCl_2_, 0.82 mM MgSO_4_, and 15 mM HEPES, pH 7.5) with gentamycin (0.1 mg ml^–1^) and streptomycin (0.1 mg ml^–1^). The incubation buffer was changed every 24 h. Oocytes injected with water were used as the negative control.

#### [^14^C]‐labelled choline uptake by *Xenopus* oocytes

The assay was performed as described previously (Kraner *et al*., 2017) with some modifications. At 72 h after injection, the oocytes were transferred into 500 μl Na‐Ringer (115 mM NCl, 2 mM KCl, 1 mM MgCl_2_, 1.8 mM CaC1_2_, and 10 mM MES‐Tris (pH 5.5) or 10 mM HEPES‐NaOH (pH 7.4)) with 0.1 mg ml^–1^ gentamycin and 5 µM choline ([methyl‐^14^C]‐choline chloride 55 mCi mmol^–1^; ARC). After incubation at 20°C for 2 h, oocytes were transferred to ice‐cold Barth’s medium, washed four times, and solubilised with 100 ml 1% (w/v) SDS. Three oocytes were used as one sample, and the accumulated radioactivity in the oocytes was measured. The experiments were repeated independently using three different batches of oocytes with similar results.

### Subcellular localisation

The full‐length open reading frame (ORF) of *ZmCTLP1* was amplified by PCR from maize B73 and cloned into pCAMBIA1300‐35S‐green fluorescent protein (GFP) using restriction enzymes and ligases. The fused pCAMBIA1300‐35S‐ZmCTLP1‐GFP construct and RFP‐SYP61 (a TGN marker) were transiently transformed into maize mesophyll protoplasts as previously described (Yoo *et al*., 2007). The GFP signal was observed and merged with the RFP signal using a ZEISS LSM710 confocal microscope (Germany) with scan speed set at ‘Best signal’. The excitation wave lengths for GFP and RFP signals were 488 and 561 nm, and the emission wave lengths were 500–586 and 598–656 nm, respectively.

### qPCR and RNA‐seq

RNA was extracted from the leaves and roots of 10‐d‐old seedlings, immature kernels (2–20 DAP at 2‐d intervals), and leaf, root, internode, node, silk, cob and husk tissue were harvested at the R1 stage (70 d after sowing) using the TRIzol reagent according to the manufacturer’s instructions (Invitrogen). cDNA was synthesised from the extracted RNA using PrimeScript RT Master Mix (Takara, Dalian, China), and qPCR was performed using the TB Green Premix Ex Taq kit (Takara) following the manufacturer’s protocols. Gene expression levels were calculated using the 2^−ΔΔCt^ relative quantification method with *Actin* as the endogenous control gene. For RNA‐seq analysis, total RNA was extracted from two biological replicates of immature 6 and 12 DAP wild‐type and *smk10* kernels using TRIzol reagent (Invitrogen). RNA‐seq libraries were constructed according to the protocol of the VAHTS mRNA‐seq v2 Library Prep Kit (Vazyme, Nanjing, China) and sequenced on the Illumina HiSeq 2500 platform to generate 150‐bp paired‐end reads. The reads were mapped to the B73 reference genome using HISAT2 2.0.4 (Kim *et al*., 2015). The unique bam files were acquired using cufflinks (v.2.2.0) (Ghosh & Chan, 2016), and FPKM values were calculated to identify differentially expressed genes (DEGs) (*P* < 0.05, log_2_ fold‐change > 1.0). GO enrichment analysis of the DEGs was performed using the agriGO singular enrichment analysis tool (http://bioinfo.cau.edu.cn/agriGO), and the DEGs were visualised using MeV (https://sourceforge.net/projects/mev‐tm4/).

### Antibodies

For production of monoclonal antibody against Mn1, a specific cDNA fragment (129–450 sequence site, representing 43–150 amino acids) of *Mn1* was cloned into pET‐28a expression vector (Amersham Biosciences, Piscataway, NJ, USA) and transformed into BL21 cells (TransGen Biotech, Beijing, China). Mn1‐His fusion protein was purified using Ni Sepharose™ 6 Fast Flow (GE Healthcare, Uppsala, Sweden), and production of antibodies in mice was performed according to standard protocol of Abmart Inc. of China. Anti‐α‐tubulin (anti‐α‐tubulin mouse monoclonal antibody, BE0031; Easybio) was purchased from EASYBIO Inc. of China.

### Immunoblot analysis

Total proteins extracted from immature wild‐type and *smk10* kernels at 4, 8 and 12 DAP were used for western blot using the methods described by Wang *et al*. (2018). The nitrocellulose membranes with the protein samples attached were incubated with anti‐Mn1 and anti‐α‐tubulin in TBST (10 mM Tris‐HCl, pH 8.0, 150 mM NaCl, 0.05% Tween‐20) with 5% milk for 2.5 h at 25°C and then washed three times using TBST for 10 min. The nitrocellulose membrane and attached protein were then incubated with the secondary antibody (anti‐rabbit IgG, horseradish peroxidase (HRP)‐linked antibody; Proteintech, Rosemont, IL, USA). HRP was detected using the Immobilon Crescendo Western HRP substrate kit (Millipore), and the signals were visualised with a C300 imaging system (Azure Biosystems, USA).

### *In* *situ* hybridisation

Paraffin sections were prepared as described above, and gene‐specific Zm*SWEET4c* and *ZmCTLP1* probes with locked nucleic acid modification was generated (Supporting Information Table S2, see later). *In situ* hybridisation was performed according to previously described protocols (Ding *et al*., 2015).

### TUNEL assay

Sections from wild‐type and *smk10* mutant kernels collected at 8 and 12 DAP were dewaxed using the HistoChoice Clearing Agent (Sigma) and hydrated through an ethanol series (95%, 90%, 80%, 70%). The sections were treated with proteinase K in phosphate buffer (pH 7.4), and the terminal deoxynucleotidyl transferase (TdT)‐mediated dNTP nick end labelling (TUNEL) assay was performed using the DeadEnd Fluorometric TUNEL Kit (Promega) according to the manufacturer’s protocol. Cell nuclei were stained using propidium iodide (PI) (Beyotime Biotechnology, Shanghai, China). The green fluorescence (the TUNEL signal) and the red fluorescence of PI were observed using a ZEISS LSM710 confocal microscope (Germany) with the parameters as described in the section of ‘Subcellular localisation’.

### Phylogenetic tree

ZmCTLP1 and its orthologous protein sequences were downloaded using MaizeGDB (https://www.maizegdb.org/) and NCBI (https://www.ncbi.nlm.nih.gov/) databases. The obtained orthologous full‐length proteins grouped together and their sequences were aligned to compare equivalent residues using the program clustalw. (https://www.megasoftware.net/). Neighbour‐joining phylogenetic trees were constructed from full‐length protein sequences, and the numbers at the nodes represent the percentage support from 1000 bootstrap replicates.

## Results

### Phenotypic and genetic characterisation of *smk10*

**Fig. 1:**
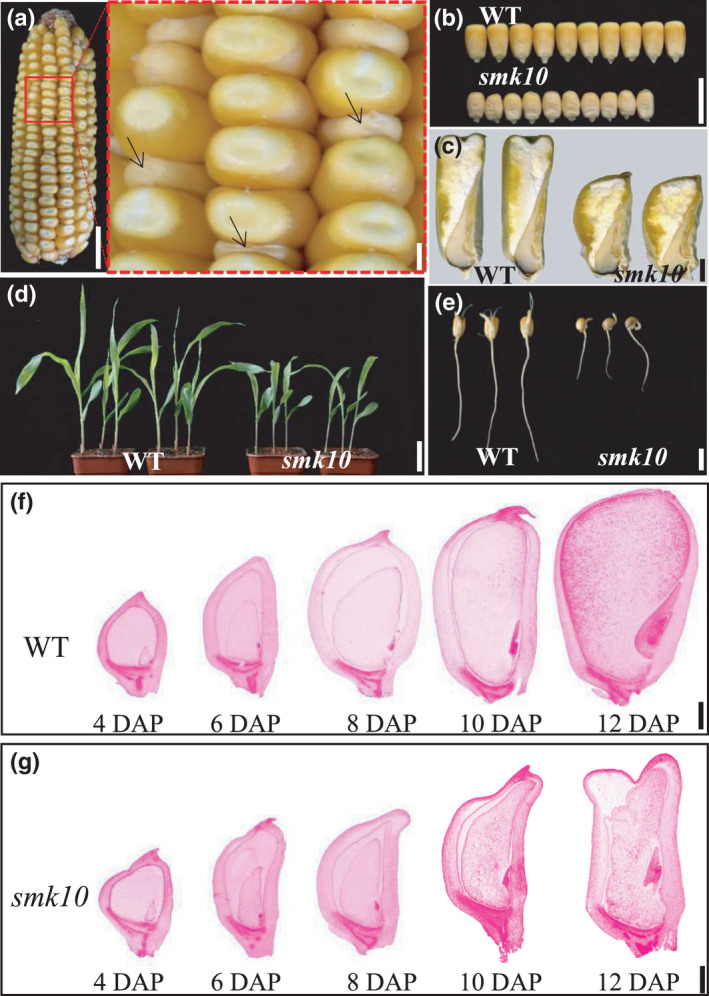
Phenotypic features of the maize *smk10* mutant. (a) Mature *smk10* × B73 F2 ear (wild‐type (WT) and *smk10* kernels segregate in a 3 : 1 ratio). Black arrows indicate *smk10* kernels. Bar, 2 cm. The magnified sections are indicated by dotted boxes. Bar, 2 mm. (b, c) Mature wild‐type and *smk10* kernels. Mature WT and *smk10* kernels (b) and their longitudinal sections (c). Bars: (b) 1 cm (c) 0.2 cm. (d) Leaf phenotypes of 10‐d‐old WT and *smk10* seedlings. Bar, 3 cm. (e) Root phenotypes of 2‐d‐old WT and *smk10* seedlings. Bar, 1 cm. (f, g) Longitudinal sections of developing WT (f) and *smk10* (g) kernels at 4–12 d after pollination (DAP). Bars: (f, g) 1 mm.

We screened a mutant library generated by EMS mutagenesis on the B73 background, aiming to identify novel regulators of kernel development based on the phenotype of small kernel size. We screened one mutant, designated *smk10* and performed three continuous backcrosses with B73 to clean the *smk10* background. We confirmed that *smk10* harboured a recessive mutation by genetic analysis, as the F2 ears segregated at a 3 : 1 ratio (χ^2^ = 0.16–0.92 < χ^2^_0.05_ = 3.84) of wild‐type vs *smk10* kernels. The average hundred‐kernel weight of *smk10* was significantly lower than that of the wild‐type (Fig. S1a). On the B73 genetic background, *smk10* exhibited reduced size of both endosperm and embryo (Figs 1a–c, S1b,c). The *smk10* mutant had a higher embryo : endosperm ratio than that of wild‐type (Fig. S1d), and its kernel development was slower (Fig. S1e). Soluble sugar and storage protein contents were much higher in mature *smk10* kernels, whereas total starch content was much lower (Fig. S2), suggesting that the kernel development process was attenuated in *smk10*. The growth of seedling leaves and roots was slower in *smk10* than that of the wild‐type, which could mainly be due to the smaller endosperm and embryo. However, there were no obvious differences between the wild‐type and *smk10* in important agronomic traits such as kernel row number, ear length, plant height, and tassel branch number at the mature stage (Figs 1d,e, S3–S5).

Paraffin sections of immature wild‐type and *smk10* kernels collected 4–12 DAP were prepared to observe morphological features during kernel development (Fig. 1f,g). The mutant kernel phenotype was distinguishable at 6 DAP, when the endosperm and pericarp showed an irregular periphery. Phenotypic differences in the embryo could be discerned at 8 DAP: the wild‐type embryo showed a visible scutellum, whereas the *smk10* embryo was still in the proembryo stage. Clear phenotypic differences were observed at 12 DAP: *smk10* exhibited an empty pericarp and a small, irregular endosperm and embryo (Figs 1f,g, S6a,b). Starch granules were smaller in *smk10* than in the wild‐type at 12 DAP and in mature kernels (Fig. S6c–h).

**Fig. 2:**
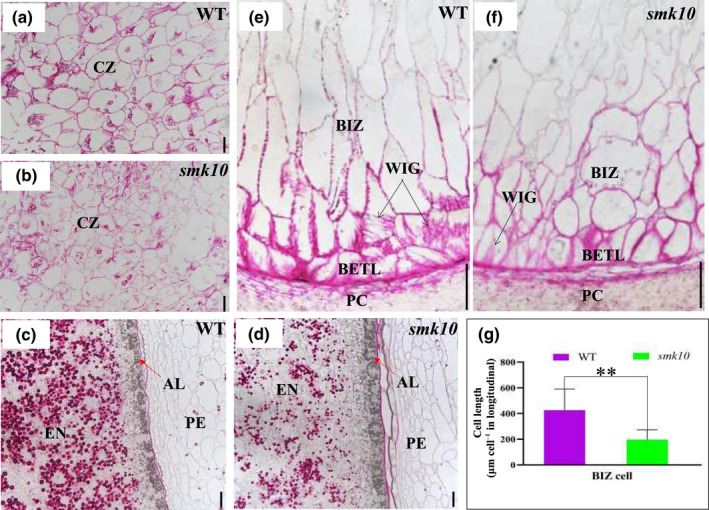
Longitudinal sections of wild‐type (WT) and *smk10* kernels in maize. (a, b) Comparison of the developing CZ in WT (a) and *smk10* (b) kernels at 12 d after pollination (DAP). CZ, conducting zone. Bars, 100 μm. (c, d) Comparison of the developing aleurone layer (AL) in WT (c) and *smk10* (d) kernels at 12 DAP. The AL is indicated by a red arrow. EN, endosperm; PE, pericarp. Bars, 20 μm. (e, f) Comparison of the developing basal endosperm transfer layer (BETL) and basal intermediate zone (BIZ) in WT and *smk10* kernels at 12 DAP. Black arrows indicate wall in‐growths (WIG) of reticulate and flange BETL cells and BIZ cells in the WT (e). Wall in‐growths (WIG) were stunted in *smk10* (f). PC, placento‐chalazal region. Bars, 50 μm. (g) The lengths of developing BIZ cells in wild‐type and *smk10* longitudinal sections. Values are means ± SE, *n* = 200 cells (**, *P* < 0.01, Student’s *t*‐test).

These phenotypic changes motivated us to investigate the morphological differences in different endosperm compartments. At 12 DAP, the wild‐type conducting zone (CZ) and central starchy endosperm (CSE) cells were fairly uniform in size and shape, whereas the *smk10* CZ and CSE cells were smaller and nonuniform with highly variable sizes (Figs 2a,b, S7a–d). The wild‐type AL cells were similar to those of *smk10* (Fig. 2c,d). In the wild‐type, the BETL displayed a characteristic slightly elongated shape with labyrinth‐like wall in‐growths (Figs 2e, S7e). By contrast, the mutant exhibited misshapen BETL cells with few thickenings or wall in‐growths (Figs 2f, S7f). The wild‐type had extremely elongated BIZ cells with wall in‐growths that decreased gradually from the second to the fourth cell layers, but the elongation and wall in‐growths were less pronounced in *smk10*, and some of its cells were rounded like those of the starchy endosperm (Fig. 2e–g). These results suggested that the *smk10* endosperm develops dysfunctional nutrient transfer cells, especially the BETL and BIZ cells, and this may block nutrient transport from the maternal tissue to endosperm.

### Map‐based cloning of *smk10*

**Fig. 3:**
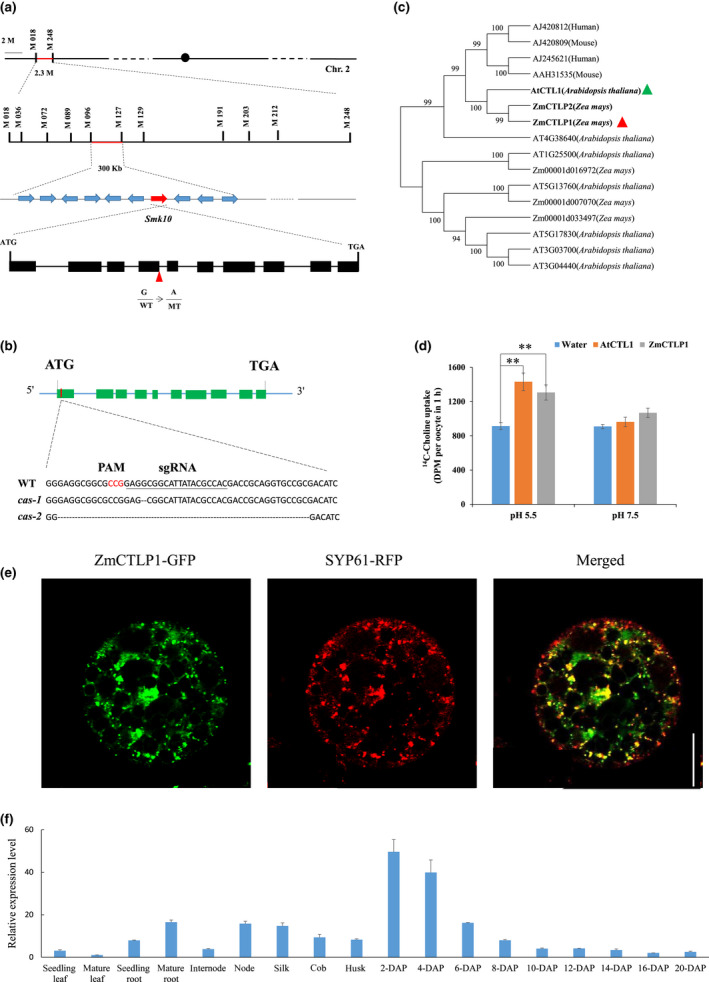
Positional cloning and identification of maize *smk10* mutant. (a) The *smk10* locus was mapped to a 300‐kb region on chromosome 2 using a high‐resolution map consisting of 3264 F3 individuals derived from a cross between *smk10* and Mo17. The red triangle denotes the site of a mutation in the predicted intron‐exon splicing site. (b) *Zm00001d001803* was targeted with a specific gRNA located in the first exon. Two independent events with different fragment deletions were generated with the CRISPR/Cas9 system. (c) Neighbour‐joining phylogenetic tree using *Arabidopsis thaliana* paralogues of AtCTL1 and maize paralogues of ZmCTLP1 (with four human and mouse protein sequences as an outgroup). Red and green triangles indicate ZmCTLP1 and AtCTL1, respectively. (d) pH‐dependent choline uptake mediated by *ZmCTLP1* in *Xenopus* oocytes. The means ± SE from five independent experiments are presented (**, *P* < 0.01, Student’s *t*‐test). (e) Subcellular localisation of ZmCTLP1 in maize protoplasts. Bar, 10 μm. (f) Expression pattern of *ZmCTLP1* in various tissues measured by qRT‐PCR. *Actin* was used as the endogenous control gene. Three biological replicates of each tissue type were analysed, and the values are reported as means ± SE.

To identify the causal gene of *smk10*, an F2 mapping population of 70 individuals was obtained from a cross between heterozygous *smk10* and Mo17. The *smk10* mutant locus was mapped to a 2.3‐Mb physical interval on chromosome 2 that was flanked by the two InDel markers Chr2‐M018 and Chr2‐M248. To narrow down the interval, a high‐resolution map was obtained from 3264 F3 individuals, and *smk10* was fine mapped to a 300‐kb region that contained 10 candidate genes following by sequencing (Fig. 3a; Tables S1, S2). RNA‐seq analysis indicated that there was a single nucleotide transition (G→A) in the 5′ splicing site of the fourth intron of the candidate *Zm00001d001803*, encoding a putative choline transporter with 10 transmembrane helices (Fig. S8a,b). Consistent with this nucleotide transition in the intron splicing site, PCR analysis with a gene‐specific primer pair (F, R) based on the *Zm00001d001803* sequence revealed at least four different transcripts in *smk10* kernels, visualised by agarose gel electrophoresis (Fig. S8c). A primer set (DJ‐1) was designed to amplify the full‐length cDNA. Subsequent cloning and sequencing of the products indicated that five alternative spliced variants with premature termination codons (PTCs) generated in upstream regions of the intact mRNA were found (Fig. S8d). Interestingly, the five alternative splicing transcripts were at least partially escaped from nonsense‐mediated mRNA decay (NMD), a surveillance pathway that degrades mRNAs containing PTCs. Although the cause of the escape remains unclear, several studies had also reported examples of NMD escapes (Ruiz‐Echevarria *et al*., 1998; Nyikó *et al*., 2009; Lindeboom *et al*., 2019).

CRISPR‐Cas9 knockouts were created to confirm that *Zm00001d001803* was responsible for the small kernel phenotype. A guide RNA was designed to target the first exon, and two knockout events with frameshifts in the first exon were identified (Figs 3b, S9, S10a). The kernels of knockout plants exhibited a serious defective kernel phenotype with empty pericarps at the crown kernels, very similar to *smk10*. An allelism test was performed by crossing *smk10* with heterozygous *cas‐1* (*cas‐1*/+), heterozygous *cas‐2* (*cas‐2*/+) and *cas‐1*; neither *cas‐1* nor *cas‐2* complemented *smk10* (Fig. S10b; Table S3). In addition, *cas‐1* developed defective basal endosperm cells that resembled those of *smk10* (Fig. S10c). To investigate whether there is maternal effect of *ZmCTLP1* or not, we performed phenotypic analysis by using kernels of reciprocal‐crossed ears derived from wild‐type (WT) (+/+) and *cas‐1* (*−/−*). No significant differences were found for 100‐kernel weight, suggesting that there was no visible maternal effect of *ZmCTLP1* (Fig. S11). All these results confirmed that a null mutation of *Zm00001d001803* is the molecular basis for the *smk10* phenotype.

### *Smk10* encodes the choline transporter ZmCTLP1 that facilitates choline transport

Smk10 and its homologous proteins were used to construct a neighbour‐joining phylogenetic tree to investigate their evolutionary relationships (Fig. S12). The sequences were divided into three clades: dicots (clade I), monocots (clade II), and human–mouse (clade III). Smk10 was grouped with the monocots, and AtCTL1 was grouped with the dicots. A phylogenetic tree containing Smk10 and its paralogues indicated that Smk10 and Zm00001d052748 were highly similar to AtCTL1 (Fig. 3c), which is a CTL protein located at the TGN (Fig. S13) (Dettmer *et al*., 2014). However, the expression of *Zm00001d052748* (named *ZmCTLP2*) was much lower than that of *ZmCTLP1* (Fig. S14a). Therefore, *Smk10* was named *ZmCTLP1*. ZmCTLP1, like other CTL proteins, is a transmembrane protein with 10 highly conserved transmembrane helices and a large extracellular loop at the N terminus, as predicted by TOPO2 (http://www.sacs.ucsf.edu/cgi‐bin/open‐topo2.py). In maize, five genes containing a conserved plasma‐membrane choline transporter domain were identified (Fig. S15). They contain four conserved amino acids (V^422^G^500^R^516^N^560^), and these sites may be functionally important. The other three maize genes with lower expression levels were less similar (<26%) to *AtCTL1* (Fig. S14a).

ZmCTLP1 is closest in homology to animal and *Arabidopsis thaliana* CTL1 proteins (Fig. S12), which have been shown to have choline transport activity (O'Regan *et al*., 2000; O'Regan & Meunier, 2003; Kommareddi *et al*., 2010; Dettmer *et al*., 2014). To examine the choline transport activity of ZmCTLP1, we expressed both *ZmCTLP1* and *AtCTL1* in *Xenopus laevis* oocytes and measured their choline uptake capacity at two pH levels (pH 5.5 and pH 7.5). The results showed that the choline uptake capacity of ZmCTLP1 was similar to that of AtCTL1 (Fig. 3d).

### Subcellular localisation and expression patterns of *ZmCTLP1*

In previous studies, AtCTL1 was localised to the TGN (Dettmer *et al*., 2014; Kraner *et al*., 2017). To examine the subcellular localisation of ZmCTLP1, the full‐length ORF of *ZmCTLP1* from the wild‐type was fused to the N terminus of GFP driven by the CaMV 35S promoter. The construct was co‐transformed into maize protoplasts with SYP61‐RFP (a TGN marker; Poulsen *et al*., 2014; Rosquete *et al*., 2018). Transient expression of ZmCTLP1‐GFP showed that it co‐localised with SYP61 (Fig. 3e), suggesting that ZmCTLP1 was located at the TGN in maize. *ZmCTLP1* was constitutively expressed in various tissues, with higher expression levels in kernels and lower expression levels in other examined tissues (Fig. 3f). During kernel development, the expression of *ZmCTLP1* was higher at 2–6 DAP and slowly decreased after 8 DAP (Figs 3f, S14a). At the protein level, the ZmCTLP1 content was higher in endosperms at 10–12 DAP (Fig. S14b). To accurately delineate the expression patterns of *ZmCTLP1* within the kernel, we performed RNA *in situ* hybridisation on 12 DAP kernels. *ZmCTLP1* expression was higher in the BETL, BIZ, aleurone layer (Al), PC, and embryo (EMB), but lower in the CSE and in the endosperm adjacent to scutellum (EAS) (Fig. S16).

### *ZmCTLP1* is involved in lipid homeostasis during kernel development

**Fig. 4:**
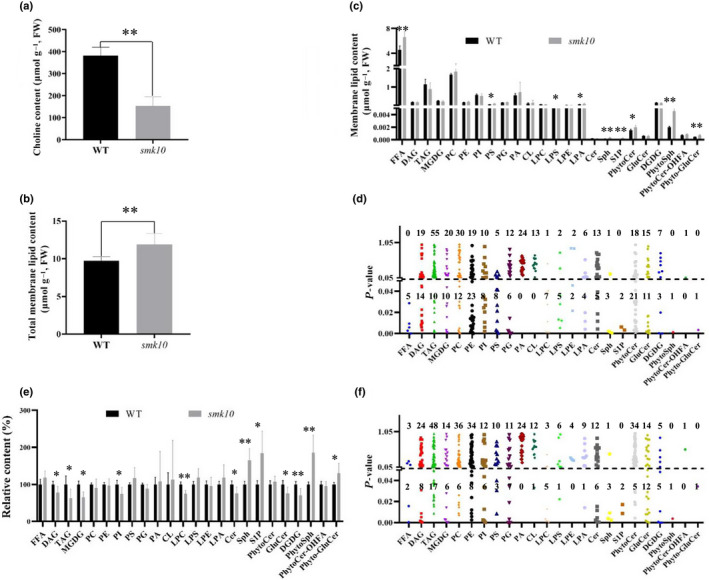
Changes in choline and lipid homeostasis accompany abnormal maize kernel development in the *smk10* mutant at 12 d after pollination (DAP). (a) Choline contents in wild‐type (WT) and *smk10* kernels. The means ± SE of four replicates per genotype are presented (**, *P* < 0.01, Student’s *t*‐test). (b) Total lipid contents in WT and *smk10* kernels. (c) Twenty‐four classes of membrane lipids were measured in WT and *smk10* kernels. (d) The absolute contents of 423 lipid species from 24 classes were measured. The *y*‐axis shows the *P‐*values associated with *t*‐tests between the WT and *smk10*. (e) The relative contents of 24 classes of membrane lipids in WT and *smk10* kernels. (f) The relative contents of 423 lipid species from 24 classes were measured. The *y*‐axis shows the *P‐*values associated with *t*‐tests between the WT and *smk10*. Cer, ceramides; CL, cardiolipins; DAG, diacylglycerols; DGDG, digalactosyl diacylglycerols; FFA, free fatty acids; GluCer, glucosylceramides; LPA, lyso‐PA; LPC, lyso‐PC; LPE, lyso‐PE; LPS, lyso‐PS; MGDG, monogalactosyl diacylglycerols; PA, phosphatidic acids; PC, phosphatidylcholines; PE, phosphatidylethanolamines; PG, phosphatidylglycerols; PhytoCer, phytoceramides; PhytoCer‐OHFA, phytoceramides with hydroxylated fatty acyls; Phyto‐GluCer, phyto‐glucosylceramides; PhytoSph, phytosphingosines; PI, phosphatidylinositols; PS, phosphatidylserines; S1P, sphingosine‐1‐phosphate; Sph, sphingosines; TAG, triacylglycerols. In (b, c, e, f) the means ± SE of five replicates per genotype are presented (**, *P* < 0.01, *, *P* < 0.05; Student’s *t*‐test).

*ZmCTLP1* functions in choline transport, and we therefore measured choline content in 12 DAP kernels using high‐performance liquid chromatography (HPLC). The *smk10* mutant had significantly lower choline content than the wild‐type (Fig. 4a), implying that *ZmCTLP1* was required for normal choline homeostasis. Choline serves as a precursor for phosphatidylcholines and sphingolipids, which are the main components of the cell membrane (Wang *et al*., 2017), and we therefore investigated the contents of 24 classes of membrane lipids using HPLC‐mass spectrometry (HPLC‐MS). Content of total lipids was higher in the *smk10* mutant (Fig. 4b), and free fatty acid (FFA) levels were also greater in *smk10* than that of wild‐type. A third of the phospholipids, including phosphatidylserines (PS), lyso‐PS (LPS), and lyso‐PA (LPA), and more than half of the sphingolipids, including phytoceramides (PhytoCer), phyto‐glucosylceramides (Phyto‐GluCer), phytosphingosines (PhytoSph), sphingosines (Sph), and sphingosine‐1‐phosphate (S1P) showed significant alteration in *smk10*. Glycolipid profiles did not differ between *smk10* and the wild‐type (Fig. 4c).

Furthermore, we also examined the global lipid profiles of wild‐type and *smk10* kernels by measuring 423 molecular species from 24 classes (Fig. 4d; Table S4). Over 30% of the individual lipids were markedly higher in the *smk10* mutant, and 6.9% were markedly lower. All FFA and most phospholipid molecular species showed significant alteration in *smk10*. Although the total contents of lyso‐PC (LPC), phosphatidylethanolamines (PE), and lyso‐PE (LPE) were similar between *smk10* and the wild‐type, more than half of the individual molecular species were significantly altered in *smk10* (Fig. 4d; Table S4). Phosphatidylcholines are the major phospholipid components of the plasma membrane. There was no significant difference in total phosphatidylcholine content between the genotypes, but 29% of the individual phosphatidylcholine species differed markedly between the wild‐type and *smk10* (Fig. S17).

Interestingly, although the absolute lipid contents were higher in *smk10*, the relative proportions of some lipid classes were significantly lower. The phospholipids including LPC and phosphatidylinositols (PI), glycolipids including monogalactosyl diacylglycerols (MGDG) and digalactosyl diacylglycerols (DGDG) etc., were markedly lower in the *smk10* than in the wild‐type (Fig. 4e). Over 16% of the individual lipids were markedly lower in the *smk10*, and 8.5% were markedly higher (Fig. 4f). As the endosperm cell size in *smk10* was smaller than that of wild‐type (Fig. S7a,b), the increased absolute contents of lipids in *smk10* might due to the large number of cells per unit weight of kernel. In fact, the total lipid content and most of individual lipids per kernel were significantly decreased in *smk10* than that of the wild‐type (Fig. S18). Overall, our results indicated that lipid homeostasis was severely disrupted in *smk10*.

### *ZmCTLP1* is crucial for PD formation in endosperm transfer cells

**Fig. 5:**
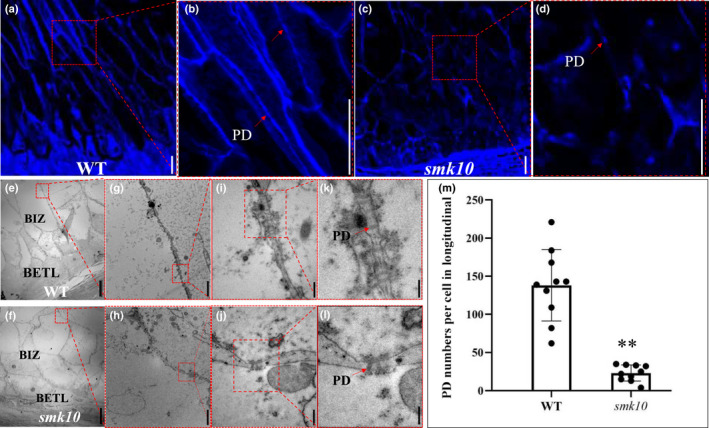
*ZmCTLP1* is essential for plasmodesmata (PD) development in maize. (a–d) Aniline blue staining of callose shows the numbers and locations of PD in 12 d after pollination (DAP) wild‐type (WT) (a) and *smk10* (c) kernels. (b, d) Magnified sections of (a) and (c) indicated by dotted boxes. PD is indicated by a red arrow. Bars, 50 μm. (e, f) Longitudinal sections of the basal endosperm transfer layer (BETL) and basal intermediate zone (BIZ) from 12 DAP WT (e) and *smk10* (f) kernels. Bars, 20 μm. (g, h) The magnified sections of (e) and (f) indicated by dotted boxes. Bars, 2 μm. (i, j) The magnified sections of (g) and (h) indicated by dotted boxes. Bars, 500 nm. (k, l) The magnified sections of (i) and (j) indicated by dotted boxes. PD structure and numbers are shown. Red arrows indicate PD. Bars, 100 nm. (m) Number of PD in 10 independent longitudinal sections of BIZ cells from 12 DAP WT and *smk10* kernels. The number of PDs differed significantly between the WT and *smk10*. The means ± SE are presented (**, *P* < 0.01; Student’s *t*‐test).

Because the transfer cells in *smk10* were misshapen, we further investigated whether the symplastic pathway of nutrient transport among transfer cells was affected. To this end, we performed aniline blue staining of paraffin sections followed by confocal microscopy to identify the location of callose deposition around the neck regions of the PD. Signals at the BIZ cell walls were significantly diminished in *smk10* compared with the wild‐type at 12 DAP (Fig. 5a–d). The number of PD in the BIZ region at 12 DAP was dramatically lower in *smk10*, but there were no obvious differences in PD morphology between the wild‐type and *smk10* (Fig. 5e–m). These observations are consistent with previous reports in *Arabidopsis thaliana* (Kraner *et al*., 2017) and show that *ZmCTLP1* is required for PD formation in endosperm transfer cells.

### Loss of *ZmCTLP1* function alters the expression of key kernel development genes

**Fig. 6:**
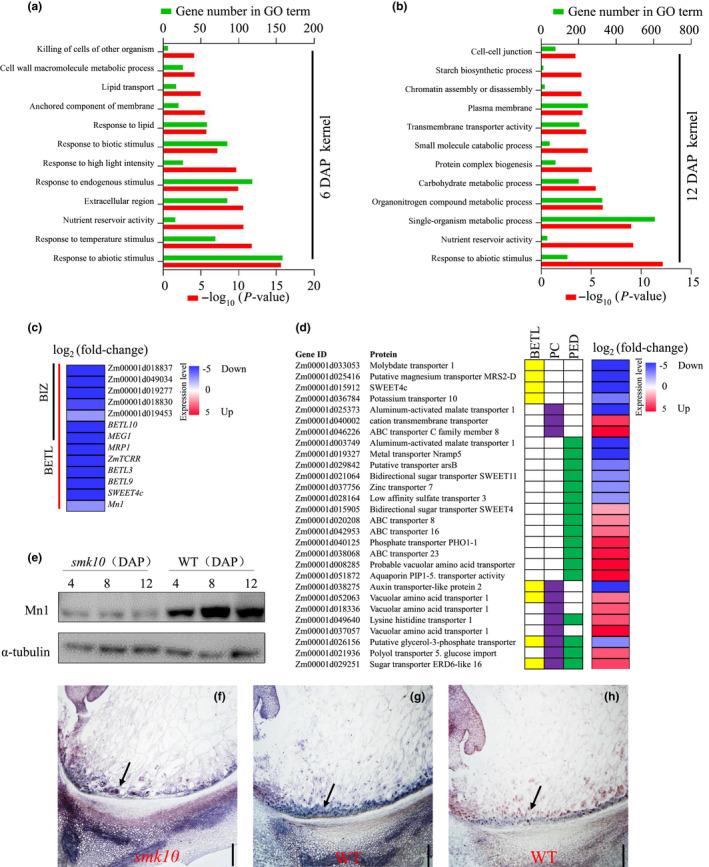
Gene Ontology analysis of the DEGs between 6 and 12 d after pollination (DAP) wild‐type (WT) and *smk10* maize kernels based on RNA‐seq data. (a, b) The most significantly enriched GO terms in the DEGs of 6 DAP kernels (a) and 12 DAP kernels (b) and their associated *P*‐values are shown. Lower x‐axis, −log_10_ (*P*‐value); upper *x*‐axis, the number of genes with a given GO term. (c) Expression of basal endosperm transfer layer (BETL) and basal intermediate zone (BIZ)‐specific genes quantified by RNA‐seq analysis. BIZ‐specific genes were obtained from Li *et al*. (2014). The log_2_ (fold‐change) values between *smk10* and the WT were calculated from RNA‐seq data and are shown as a heat map. (d) Tissue‐specific expression patterns of transport‐related genes associated with kernel development quantified by RNA‐seq analysis. PC (purple), placento‐chalazal region; PED (green), pedicel. The log_2_ (fold‐change) values between *smk10* and the WT were calculated from RNA‐seq data and are shown as a heat map (right). (e) Immunoblot analysis showing Mn1 protein accumulation in 4, 8, and 12 DAP WT and *smk10* kernels. α‐Tubulin served as the loading control. (f–h) *In situ* hybridization of *ZmSWEET4c* using antisense probes in sections of *smk10* and WT kernels at 12 DAP. (f, g) *ZmSWEET4c*‐antisense probes show the expression of *ZmSWEET4c* in the BETL of *smk10* and wild‐type kernels. (h) The *ZmSWEET4c*‐sense probe served as the negative control. The BETL is indicated by a black arrow. Bars, 200 μm.

To better understand the impacts of *smk10* on global gene expression, RNA‐seq analysis was performed on 6 and 12 DAP *smk10* and wild‐type kernels. In total, we identified 578 DEGs (97 upregulated and 481 downregulated in *smk10*) in 6 DAP kernels and 3477 DEGs (1322 upregulated and 2155 downregulated) in 12 DAP kernels (*P* < 0.05, log_2_fold‐change > 1.0) (Table S5). Here, 10 DEGs were selected for qRT‐PCR validation of the RNA‐seq results, and both methods showed similar expression differences between the wild‐type and *smk10* (Fig. S19). Gene Ontology (GO) analysis of DEGs in 6 DAP kernels revealed enrichment of the GO terms ‘response to abiotic stimulus’, ‘nutrient reservoir activity’, ‘response to lipid’, ‘cell wall macromolecule metabolic process’ and ‘killing of cells of other organism’ (Fig. 6a). The DEGs at 12 DAP were enriched in GO terms related to nutrient metabolism and transport such as ‘nutrient reservoir activity’, ‘carbohydrate metabolic process’, ‘transmembrane transporter activity, and ‘cell–cell junction (PD)’ (Fig. 6b). To further explore GO terms enriched in DEGs from different endosperm compartments, we analysed available data from a laser‐capture microdissection experiment (Zhan *et al*., 2015). Enriched GO terms included ‘cell killing’ in the BETL at 6 and 12 DAP, ‘hydrolase activity, acting on glycosyl bonds’ in the PE, and ‘ion homeostasis’ and ‘ion transport’ in the PED at 12 DAP (Fig. S20; Table S6).

Eight BETL‐specific genes, including *Mn1* and *ZmSWEET4c*, were significantly downregulated in *smk10* (Fig. 6c). Tissue‐specific transporters for ions, amino acids and sugars located in the PED, PC, and BETL were also differentially expressed in *smk10* at 12 DAP (Fig. 6d). In fact, *c*. 11.5% (90/784) of all transporters in *smk10* were differentially expressed at 12 DAP; these included 27 ion transporters, 13 sugar transporters, nine amino acid transporters, and two lipid transporters (Table S7).

*Mn1* and *ZmSWEET4c* are essential for the development of fully differentiated transfer cells. To measure the abundance of Mn1 protein, total proteins were isolated from immature wild‐type and *smk10* kernels at 4, 8 and 12 DAP and analysed by western blotting with anti‐Mn1 mouse monoclonal antibody. Mn1 protein accumulation was significantly lower in *smk10* than in the wild‐type (Fig. 6e). To accurately determine the expression level of *ZmSWEET4c* in *smk10* and the wild‐type, we performed RNA *in situ* hybridisation with 12 DAP kernels. Consistent with the RNA‐seq results, *ZmSWEET4c* expression was again significantly lower in *smk10* than in the wild‐type (Fig. 6f–h). These results indicated that *ZmCTLP1* influenced the expression of key kernel development genes.

### Loss of *ZmCTLP1* causes premature cell death in transfer tissues

**Fig. 7:**
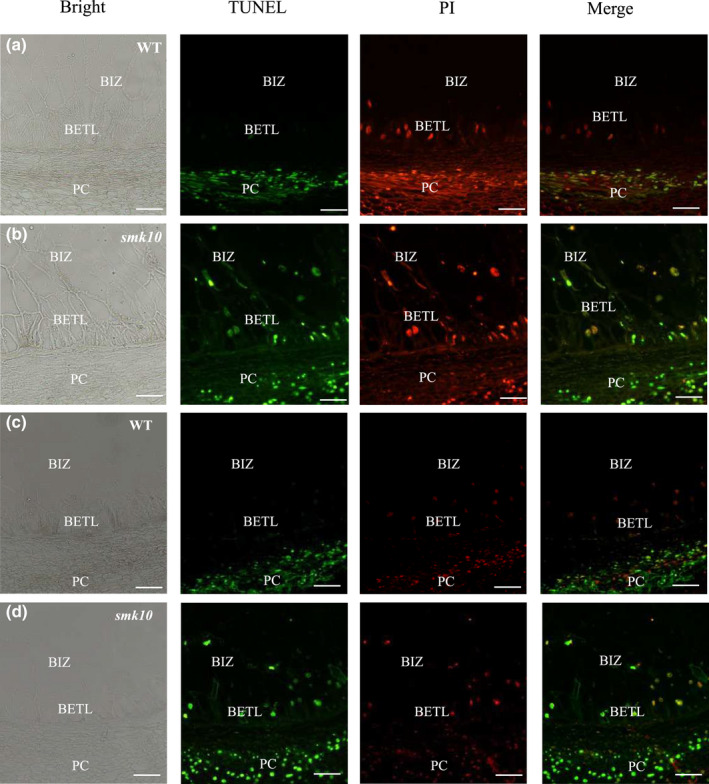
Cell death in wild‐type (WT) and *smk10* maize kernels analysed by the TUNEL assay. Green fluorescent dots indicate the TUNEL signals of fragmented DNA, and red fluorescent dots indicate nuclei stained with PI. (a, b) Cell death detected by the TUNEL assay in 8 d after pollination (DAP) WT (a) and *smk10* (b) kernels. (c, d) Cell death detected by the TUNEL assay in 12 DAP wild‐type (c) and *smk10* (d) kernels. BETL, basal endosperm transfer layer; BIZ, basal intermediate zone; PC, placento‐chalazal region. Bars, 50 μm.

Disruption of choline and lipid homeostasis can trigger cell death in plants and animals (Li *et al*., 1998, 2020; Hirasawa *et al*., 2004; Lin *et al*., 2008), and we were therefore motivated to investigate cell death in the BETL and BIZ of *smk10*. We performed a TUNEL assay to examine this cell death signal *in situ* at 8 and 12 DAP. Intense positive TUNEL signals that co‐localised with cell nuclei were detected at the BETL and BIZ positions in *smk10*, whereas no TUNEL signals were detected in the wild‐type (Fig. 7). We also observed that both the PC and EAS exhibited similar cell death in the wild‐type and the mutant, as reported by Kladnik *et al*. (2004) and Doll *et al*. (2020) (Figs 7, S21). The transfer cells are extremely active and do not undergo programmed cell death before maturity in normal maize kernels (Gao *et al*., 1998). Therefore, these findings indicated that *smk10* exhibited premature BETL and BIZ cell death, which presumably attenuates nutrient transport from the maternal placenta to the filial tissues.

## Discussion

Choline and its metabolism have been studied extensively in animals and humans. Despite its important physiological functions, little information is known about the role of choline in plants (Michel *et al*., 2006; Dettmer *et al*., 2014; Iwao *et al*., 2016). In *Arabidopsis thaliana*, choline deficiency impairs phloem development and conductivity by reducing the number of sieve pores and altering the pore structure in sieve areas (Dettmer *et al*., 2014). In this study, the *smk10* mutant contained less choline than the wild‐type and exhibited an irregular and small endosperm, a small embryo and significantly lower kernel weight. BETL and BIZ cells in the basal endosperm region of the mutant displayed irregular shapes and had few wall thickenings and wall in‐growths. We therefore concluded that loss of *ZmCTLP1* function disrupted choline and lipid homeostasis of transfer cells, thereby reducing nutrient transport from the maternal placenta to the developing endosperm.

### *ZmCTLP1* is required for the normal expression of *Mn1* and other nutrient transporters

**Fig. 8:**
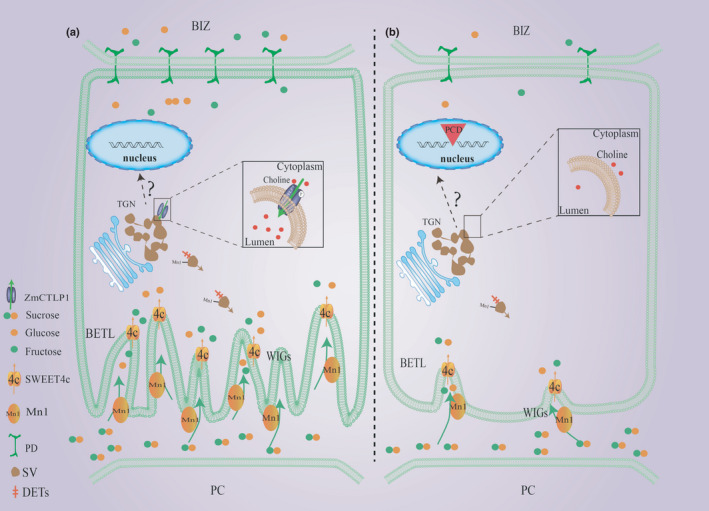
Working model for the control of maize kernel development by *ZmCTLP1*. (a) In wild‐type kernels, *ZmCTLP1* maintains lipid homeostasis, normal plasmodesmata (PD) numbers, *Mn1* and *ZmSWEET4c* expression and the function of wall in‐growths (WIG) in transfer cells through choline transport. (b) In *smk10* kernels, altered choline uptake, transport and/or metabolism affect lipid homeostasis and reduce the number of PDs. The basal endosperm transfer cells undergo premature cell death. DET, differentially expressed transporter; SV, secretory vesicle.

Membrane lipid composition is crucial for maintaining the correct structure and function of membrane proteins (Sanders & Mittendorf, 2011). Choline, an essential membrane lipid component, is predominantly used for the synthesis of phosphatidylcholine and sphingomyelin (Michel *et al*., 2006; Wang *et al*., 2017). *HP* *C1* (*High Phosphatidylcholine 1*) encodes a phospholipase A1 enzyme; when introgressed from teosinte *Mexicana*, it induces early maize flowering by modulating phosphatidylcholine levels (Rodríguez‐Zapata *et al*., 2021). Here, we showed that *ZmCTLP1* is required for kernel development and that it appears to regulate choline transport to maintain the homeostasis of membrane lipids. Nutrient transfer tissues such as the BETL and BIZ have convoluted wall in‐growths that increase the surface area of the plasma membrane, thereby enhancing nutrient transport efficiency (Kang *et al*., 2009). The formation of cell wall architecture is a membrane‐related process that relies on the supply of lipids, proteins and other components (Liu *et al*., 2015; Rosquete *et al*., 2018). Here, loss of *ZmCTLP1* function significantly disrupted choline and lipid homeostasis, and this appeared to block the formation of wall in‐growths. Mn1 is synthesised on the endoplasmic reticulum and delivered to the wall in‐growths by the TGN (Kang *et al*., 2009). Mn1 and wall in‐growths are interdependent, reinforcing each other (Kang *et al*., 2009). Our results suggested that ZmCTLP1‐mediated regulation of membrane lipid homeostasis is required for the normal accumulation of Mn1 (Figs 6e, 8).

Lipid‐dependent protein sorting plays a central role in cargo sorting and trafficking to the cell surface (Surma *et al*., 2012). Disruption of membrane lipid homeostasis in the *smk10* mutant appeared to strongly influence membrane transport capacity, as 11.5% of all transporter genes were differentially expressed in the mutant, including ion transporters, amino acid transporters and sugar transporters (Table S7). *In situ* hybridisation suggested that *ZmSWEET4c*‐mediated hexose import may have been reduced in *smk10* (Fig. 6f–h). It has been proposed that ZmSWEET4c‐mediated sugar accumulation in the BETL induces hexose import by increasing membrane area of the basal cells in developing endosperm, therefore the capacity to hold transporters (including *ZmSWEET4c*) exists as a result of increased ZmSWEET4c activity. In our study, loss of function of *ZmCTLP1* disrupted lipid homeostasis and led to incomplete wall in‐growth of BETL, which could decrease the membrane area in transfer cells, resulting in reduced accumulations of Mn1 and ZmSWEET4c in BETL. Thereby, the overall activities of hydrolysing sucrose by Mn1 and importing hexose into BETL by ZmSWEET4c were decreased, which might be one of the major causes for small kernel phenotype in *smk10*.

### *ZmCTLP1* is essential for PD formation and development

PD are membrane‐lined channels between neighbouring cells that provide a direct route for the symplastic movement of proteins, RNAs, sugars and other small molecules (Wu *et al*., 2016; Sager & Lee, 2018). During the development of primary PD, materials derived from ER tubules (desmotubules) are enclosed by cytoplasmic strands, and TGN secretory vesicles provide wall materials and membrane lipids (Ehlers & Kollmann, 2001). Sphingolipids that are enriched in the TGN perform essential functions to regulate PD permeability (Albright *et al*., 1996). Our results showed that sphingolipid levels differed significantly between *smk10* and the wild‐type, and it is plausible that disordered membrane lipid homeostasis in *smk10* may have caused the defects in PD formation. There were fewer PDs in the *smk10* mutant than in the wild‐type, indicating that the mutant was impaired in PD formation and development. The morphological abnormalities of the BETL cells in *smk10* were partly attributable to reduced numbers of PD, which would have disrupted cell‐to‐cell movement of nutrients and other signals (Fig. 8). PD numbers are also reduced in the shoot apical meristems and developing leaves of the *atctl1* mutant, causing a severely stunted growth phenotype. Soluble sugar and starch contents are significantly increased in the *atctl1* mutant, suggesting that nutrient homeostasis is disrupted in the leaves (Kraner *et al*., 2017). Our results showed that starch, sugar and protein contents were significantly altered in *smk10* kernels, and this may have resulted from defects in cell–cell symplastic transport mediated by PD.

## Author contributions

WS, JL and MH designed the experiments. MH, HZ, BY, SY, HL, HT, GS, ZC and LE performed the experiments. MH and WS, analysed the data. MH, WS and JL wrote the article. MH, HZ and BY contributed equally to this work.

## Supporting information


**Fig. S1** Analysis of wild‐type and *smk10* kernels at the mature stage and kinetics of kernel and endosperm development.
**Fig. S2** Biochemical analysis of mature wild‐type and *smk10* kernels.
**Fig. S3** Phenotypes of wild‐type and *smk10* seedlings at different time points.
**Fig. S4** The reproductive growth stage of wild‐type and *smk10* plants at 70 d after sowing.
**Fig. S5** Measurements of four agronomic traits in the wild‐type and *smk10*.
**Fig. S6** Kernel features and starch granules of the wild‐type and *smk10*.
**Fig. S7** Statistical analysis of CZ and CSE cell areas and longitudinal sections of wild‐type and *smk10* kernels.
**Fig. S8** Characteristics of the *Smk10* gene.
**Fig. S9** Sequences of the kernels from WT and CRISPR‐generated mutants in the *Zm00001d001803* genomic fragment.
**Fig. S10** Confirmation that *Zm00001d001803* is responsible for the defective kernel phenotype.
**Fig. S11** Investigation of the maternal effect for *ZmCTLP1*.
**Fig. S12** A neighbour‐joining phylogenetic tree of ZmCTLP1 and its orthologous proteins from other organisms.
**Fig. S13** The CTL1 transmembrane helix is conserved among different species.
**Fig. S14** Expression patterns of five maize genes (Chen *et al*., 2014) and the abundance of ZmCTLP1 protein (Walley *et al*., 2016) in various tissues.
**Fig. S15** Comparison of the conserved domains of CTLs in maize, *Arabidopsis thaliana* and rice.
**Fig. S16** *In situ* hybridisation of *ZmCTLP1* using antisense probes in sections of wild‐type kernels at 12 DAP.
**Fig. S17** Forty‐two phosphatidylcholine species were identified in wild‐type and *smk10* kernels.
**Fig. S18** Lipid contents per kernel between *smk10* and wild‐type at 12 DAP.
**Fig. S19** qPCR confirmation of 10 selected DEGs at 12 DAP.
**Fig. S20** Gene Ontology analysis of the DEGs between wild‐type and *smk10* kernels at 6 and 12 DAP.
**Fig. S21** Cell death detected by the TUNEL assay in 12 DAP wild‐type (a–c) and *smk10* (d–f) kernels.
**Table S1** The candidate genes predicted by maizeGDB in the 300‐kb *smk10* interval.
**Table S2** Primers used in this study.
**Table S3** Chi‐squared tests of the mature kernel phenotypes in *smk10* × CRISPR‐generated mutants ears (χ^2^_0.05_ = 3.84).
**Table S4** The contents of 423 lipid species in wild‐type (B1–B5) and *smk10* (A1–A5) kernels.
**Table S6** Enriched GO terms analysis of compartments‐specific genes in *smk10* mutant compared with wild‐type.
**Table S7** Expression of transporter genes in the DEGs quantified by the RNA‐seq analysis.

**Table S5** Expressed genes in 6 and 12 DAP *smk10* kernels compared with wild‐type.
Please note: Wiley Blackwell are not responsible for the content or functionality of any Supporting Information supplied by the authors. Any queries (other than missing material) should be directed to the *New Phytologist* Central Office.

## Data Availability

Sequence data from this article can be found in the MaizeGDB database under the following accession numbers: Zm00001d001803 (*ZmCTLP1*); Zm00001d052748 (*ZmCTLP2*); Zm00001d007070; Zm00001d016972; Zm00001d033497. *ZmCTLP1* orthologues cited in this article can be found in the NCBI’s Nucleotide database under the following accession numbers: AT3G15380 (*AtCTL1*); AT1G25500; AT3G03700; AT3G04440; AT4G38640; AT5G13760; AT5G17830 in *Arabidopsis thaliana*, and AJ420812; AJ245621 in human, and AJ420809; AAH31535 in mouse. The generated raw reads have been uploaded to NCBI’s Sequence Read Archive (SRA) database and are available under the accession number PRJNA720421.
