# Supplementary material for: ZmCTLP1 is required for the maintenance of lipid homeostasis and the basal endosperm transfer layer in maize kernels
Source: New Phytol. 2021 Oct 11;232(6):2384–99. doi: 10.1111/nph.17754 (PMC9292782; doi:10.1111/nph.17754)
Supplement: Supplementary file 1 — Fig. S1 Analysis of wild‐type and smk10 kernels at the mature stage and kinetics of kernel and endosperm development. Fig. S2 Biochemical analysis of mature wild‐type and smk10 kernels. Fig. S3 Phenotypes of wild‐type and smk10 seedlings at different time points. Fig. S4 The reproductive growth stage of wild‐type and smk10 plants at 70 d after sowing. Fig. S5 Measurements of four agronomic traits in the wild‐type and smk10. Fig. S6 Kernel features and starch granules of the wild‐type and smk10. Fig. S7 Statistical analysis of CZ and CSE cell areas and longitudinal sections of wild‐type and smk10 kernels. Fig. S8 Characteristics of the Smk10 gene. Fig. S9 Sequences of the kernels from WT and CRISPR‐generated mutants in the Zm00001d001803 genomic fragment. Fig. S10 Confirmation that Zm00001d001803 is responsible for the defective kernel phenotype. Fig. S11 Investigation of the maternal effect for ZmCTLP1. Fig. S12 A neighbour‐joining phylogenetic tree of ZmCTLP1 and its orthologous proteins from other organisms. Fig. S13 The CTL1 transmembrane helix is conserved among different species. Fig. S14 Expression patterns of five maize genes (Chen et al., 2014) and the abundance of ZmCTLP1 protein (Walley et al., 2016) in various tissues. Fig. S15 Comparison of the conserved domains of CTLs in maize, Arabidopsis thaliana and rice. Fig. S16 In situ hybridisation of ZmCTLP1 using antisense probes in sections of wild‐type kernels at 12 DAP. Fig. S17 Forty‐two phosphatidylcholine species were identified in wild‐type and smk10 kernels. Fig. S18 Lipid contents per kernel between smk10 and wild‐type at 12 DAP. Fig. S19 qPCR confirmation of 10 selected DEGs at 12 DAP. Fig. S20 Gene Ontology analysis of the DEGs between wild‐type and smk10 kernels at 6 and 12 DAP. Fig. S21 Cell death detected by the TUNEL assay in 12 DAP wild‐type (a–c) and smk10 (d–f) kernels. Table S1 The candidate genes predicted by maizeGDB in the 300‐kb smk10 interval. Table S2 Primers used in this study. Table [file NPH-232-2384-s001.pdf]

***New Phytologist* Supporting Information**

Article title: ***ZmCTLP1* is required for the maintenance of lipid homeostasis and the basal endosperm transfer layer in maize kernels**

Authors: Mingjian Hu[1†], Haiming Zhao[†1], Bo Yang[1†], Shuang Yang[1], Haihong Liu[3], He Tian[4], Guanghou Shui[4], Zongliang Chen[1,5], Lizhu E[1,2], Jinsheng Lai[1,2], Weibin Song[1,2]*

Article acceptance date: 15 September 2021

The following Supporting Information is available for this article:

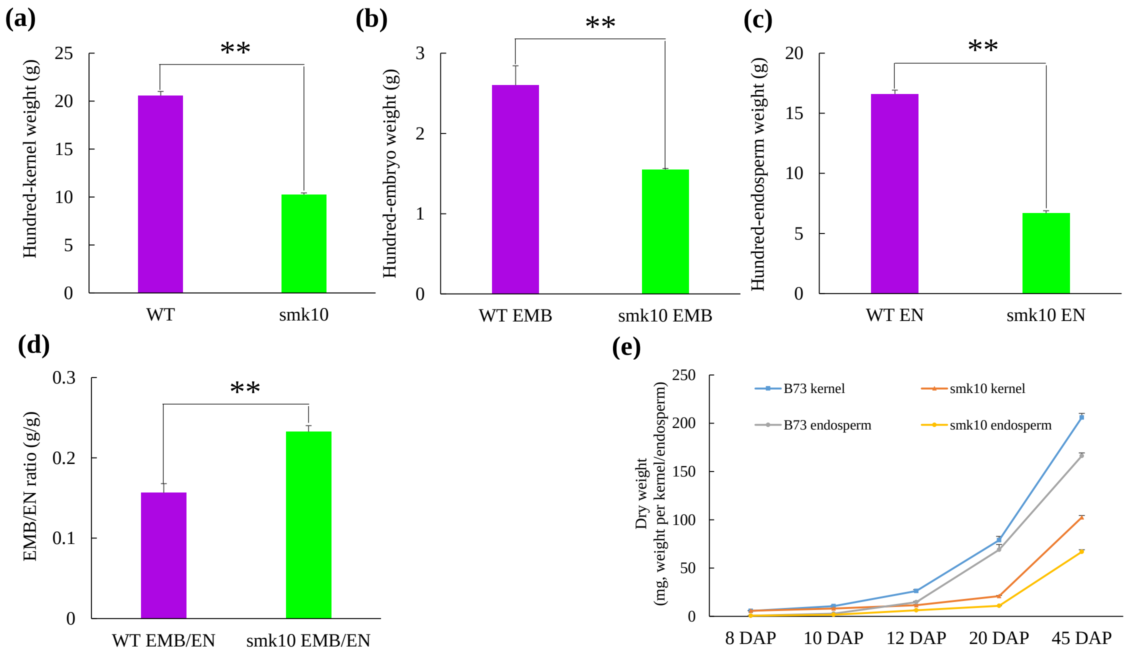

Fig. S1 Analysis of wild-type and *smk10* kernels at the mature stage and kinetics of kernel and endosperm development. The means ± SE of three replicates per genotype are presented (***p* < 0.01, Student's *t*-test). (a) Hundred-kernel weight of mature wild-type and *smk10* kernels (b, c) Embryo (b) and endosperm (c) weight of mature wild-type and *smk10* kernels. EMB, embryo;

EN, endosperm. (d) Embryo/endosperm ratio of mature wild-type and *smk10* kernels. EMB, embryo; EN, endosperm. (e) Developmental kinetics of the kernel and endosperm (Forty kernels or endosperms of wild-type and *smk10* dried at 60 ℃ for each repetition).

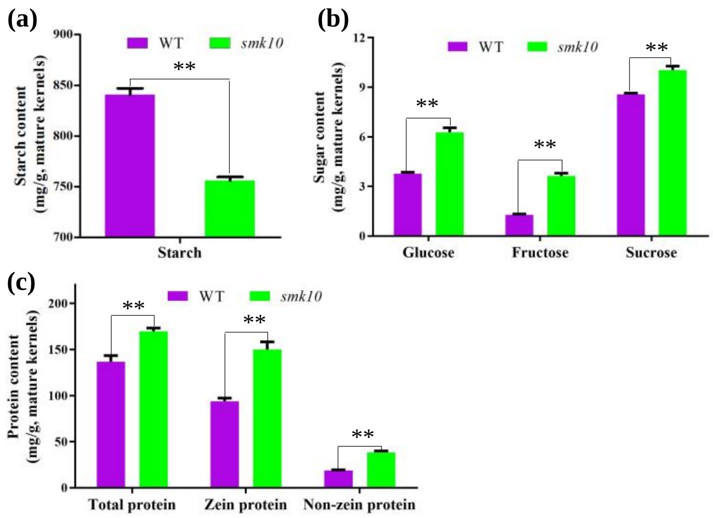

Fig. S2 Biochemical analysis of mature wild-type and *smk10* kernels. The means ± SE of four replicates per genotype are presented (**p < 0.01, Student's t-test). (a) Starch contents of wild-type and *smk10* kernels. (b) The contents of different sugars in wild-type and *smk10* kernels. (c) The contents of total protein, zein protein, and non-zein protein in wild-type and *smk10* kernels.

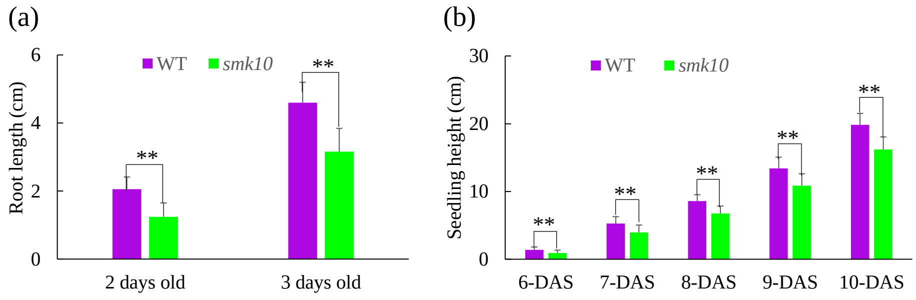

Fig. S3 Phenotypes of wild-type and *smk10* seedlings at different time points. DAS, days after sowing. (a, b) Statistical analysis of root length (a) and seedling height (b) from wild-type and *smk10* seedlings. The means ± SE are presented (n = 42, **p < 0.01, Student's t-test).

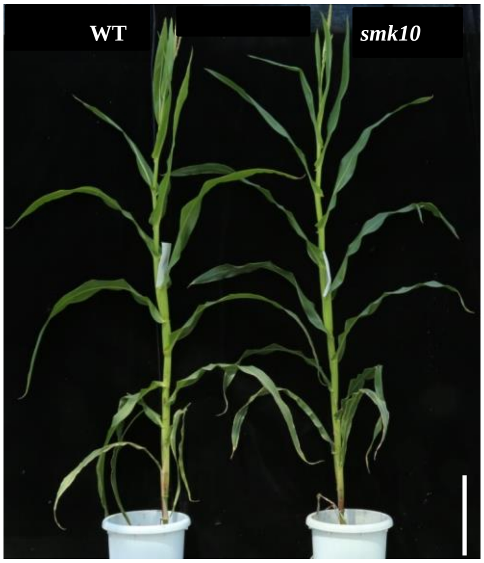

Fig. S4 The reproductive growth stage of wild-type and *smk10* plants at 70 days after sowing. Bar = 50 cm.

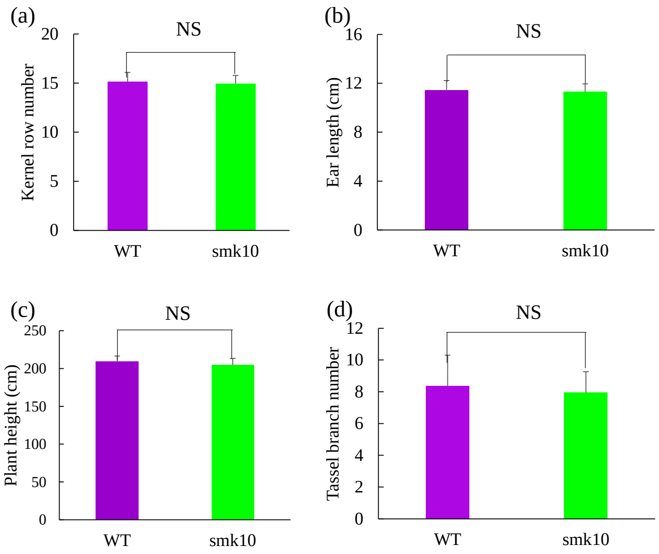

Fig. S5 Measurements of four agronomic traits in the wild type and *smk10*. (a, b) Kernel row number and ear length were measured using mature wild-type and *smk10* ears. (c, d) Plant height and tassel branch number were measured at 80 days after sowing. The means ± SE are presented (n = 20 in (a)-(d), $p > 0.05$, Student's t-test). NS, not significant.

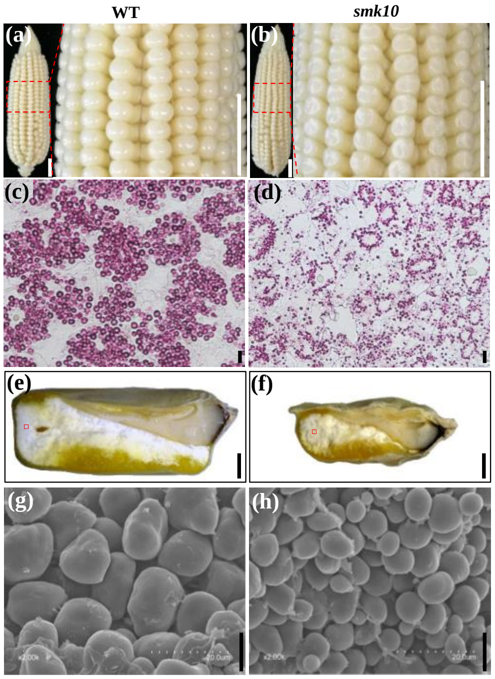

Fig. S6 Kernel features and starch granules of the wild type and *smk10*. (a, b) Features of 12 DAP wild-type (a) and *smk10* (b) kernels. Bar = 2 cm. (c, d) Light microscope images of the starch granules in 12 DAP wild-type (c) and *smk10* (d) kernels. Bar = 20 µm. (e–h) SEM analysis of starch granules in mature endosperm (red box) of wild-type and *smk10* kernels. Longitudinal sections of wild-type (e) and *smk10* (f) kernels. Scanning electron microscopy analysis of wild-type (g) and *smk10* (h) kernels. Bar = 2 mm for e, f and 10 µm for g, h.

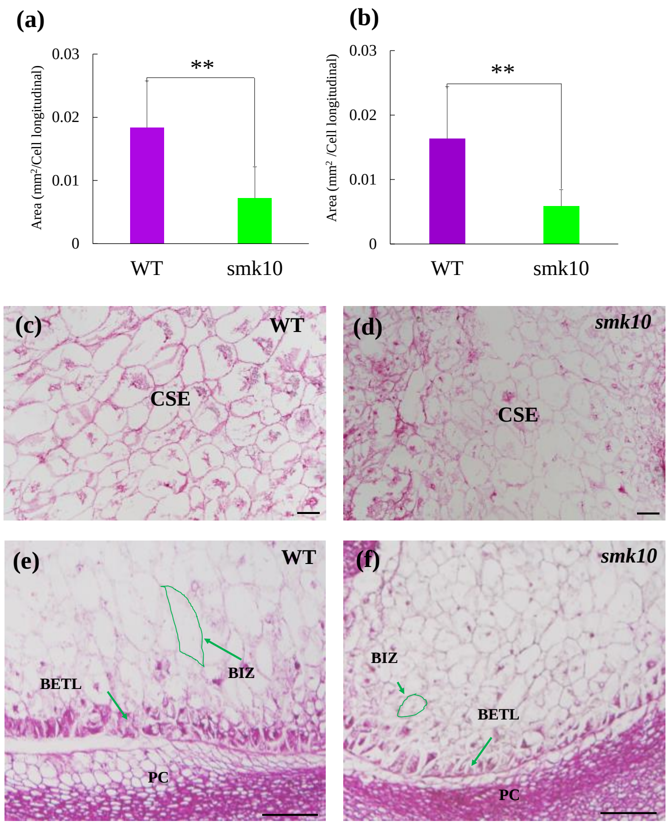

Fig. S7 Statistical analysis of CZ and CSE cell areas and longitudinal sections of wild-type and *smk10* kernels. (a, b) Statistical analysis of CZ (a) and CSE (b) cell areas based on measurements of 200 independent longitudinal cells from 12 DAP wild-type and *smk10* kernels. The means ± SE are presented (n = 200, **$p < 0.01$, Student's t-test). (c, d) Comparison of the developing CSE in wild-type (c) and *smk10* (d) kernels at 12 DAP. CSE, central starchy endosperm. Bar = 100 μm. (e, f) Longitudinal sections of 6 DAP wild-type (e) and *smk10* (f) kernels. BETL, basal endosperm transfer layer; BIZ, basal intermediate zone; PC, placento-chalazal region. Bar = 100 μm.

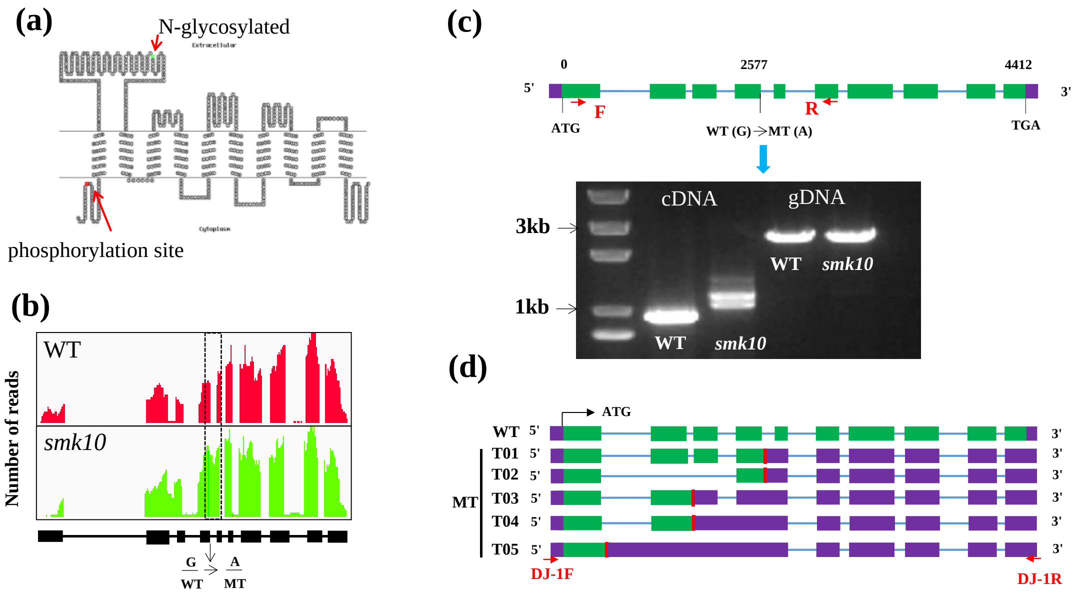

Fig. S8 Characteristics of the *Smk10* gene. (a) Schematic diagram of *Smk10* gene structure. (b) Coverage of the parallel analysis of RNA-sequencing reads from the wild type and *smk10*. The inset shows the substitution in the predicted splicing site of *Smk10* and the retained fourth intron in *smk10*. (c) A schematic representation of the *Zm00001d001803* gene and its transcript variants in *smk10*. F and R represent a pair of primers used for alternative splicing analysis. (d) A mutation (G to A) in the predicted splicing site between the fourth exon and the fourth intron yielded five types of transcripts in *smk10* mutant. The red lines represent the premature termination codon. DJ-1F and DJ-1R represent a pair of primers used to amplify the full-length cDNA. In (c) and (d), the green boxes indicate CDSs in the middle. The purple boxes indicate untranslated regions (UTRs) on the two sides. Lines indicate introns.

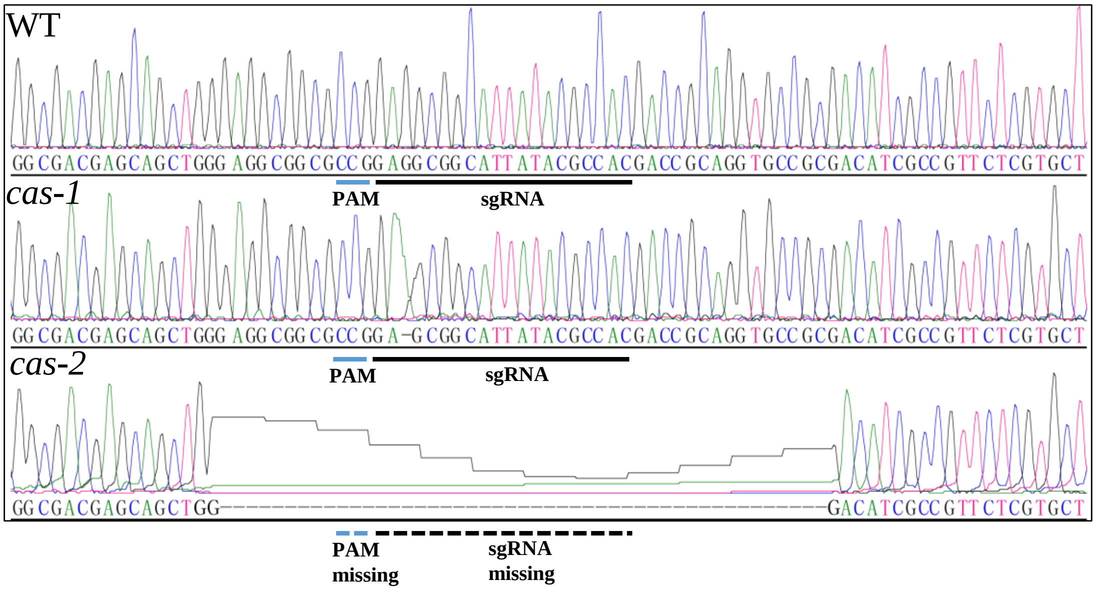

Fig. S9 Sequences of the kernels from WT and CRISPR-generated mutants in the *Zm00001d001803* genomic fragment. Sequence alignment was performed using SeqMan program.

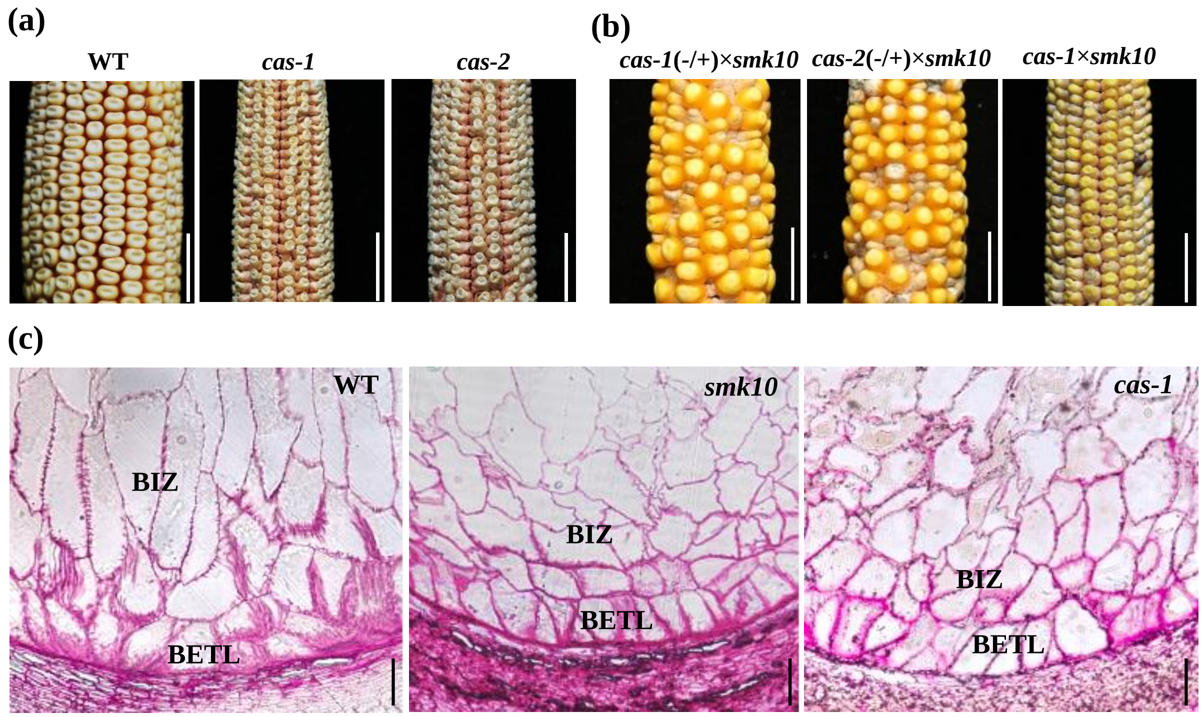

Fig. S10 Confirmation that *Zm00001d001803* is responsible for the defective kernel phenotype. (a) Defect in kernel development of *Zm00001d001803*-knockout mutants. Self-pollinated ears of

the wild type and CRISPR/Cas9-mediated knockout mutants (*cas-1* & *cas-2*). Bar = 2 cm. (b) An allelism test was performed by crossing *smk10* with *cas-1* (*cas-1*/+), *cas-2* (*cas-2*/+) and *cas-1*. Bar = 2 cm. (c) Longitudinal sections of wild-type, *smk10*, and *cas-1* mutant kernels. Bar = 20 μm.

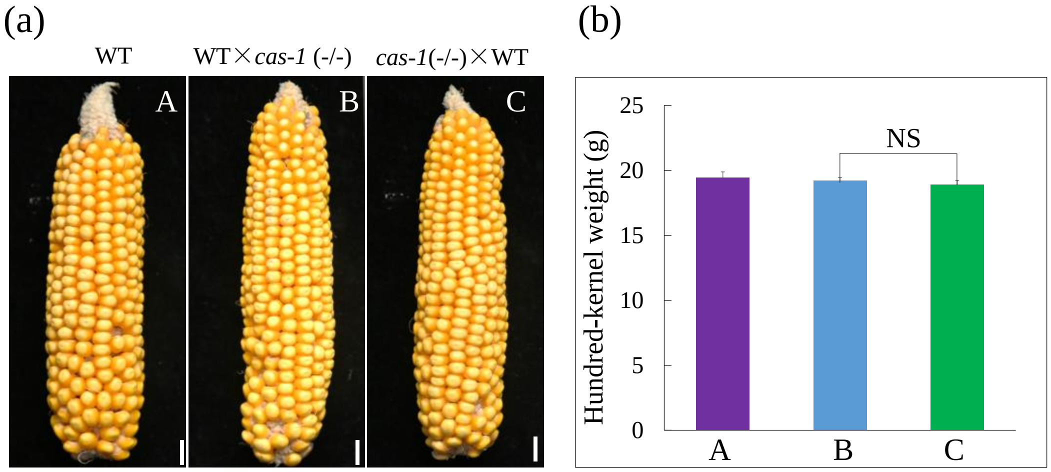

Fig. S11 Investigation of the maternal effect for *ZmCTLP1*. (a) Reciprocal crosses were performed by crossing WT with *cas-1* mutant. Bar = 1 cm. (b) Hundred-kernel weight of wild type (A), WT × *cas-1* (B) and *cas-1* × WT (C) at mature stage. The means ± SE of four replicates per genotype are presented ($p > 0.05$, Student's t-test). NS, not significant.

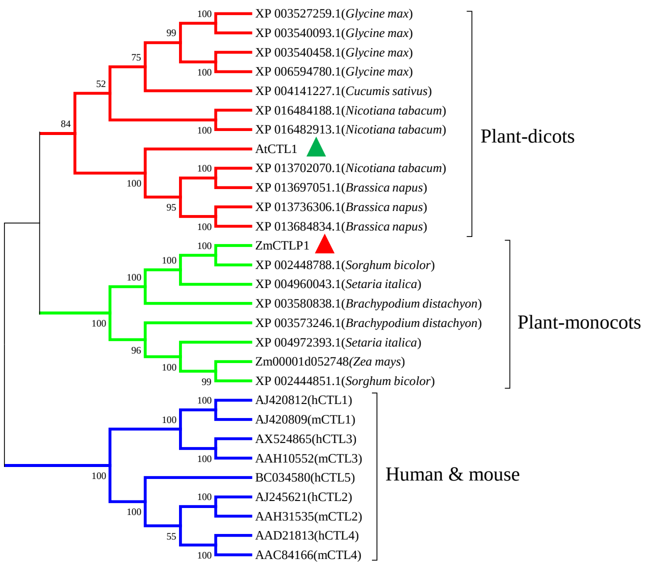

Fig. S12 A neighbor-joining phylogenetic tree of ZmCTLP1 and its orthologous proteins from other organisms. Red and green triangles indicate ZmCTLP1 and AtCTL1, respectively.

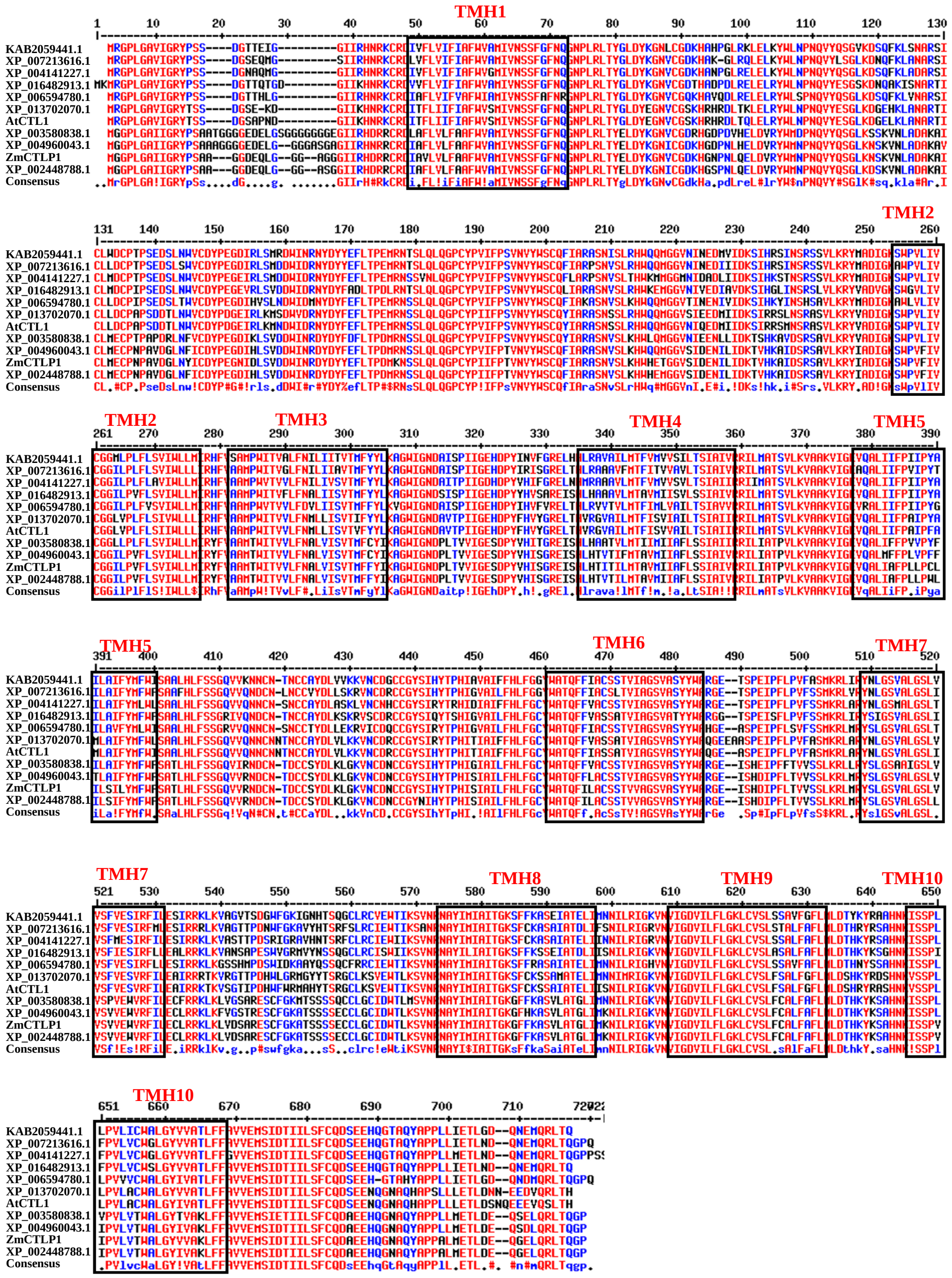

Fig. S13 The CTL1 transmembrane helix is conserved among different species. TMH, transmembrane helix.

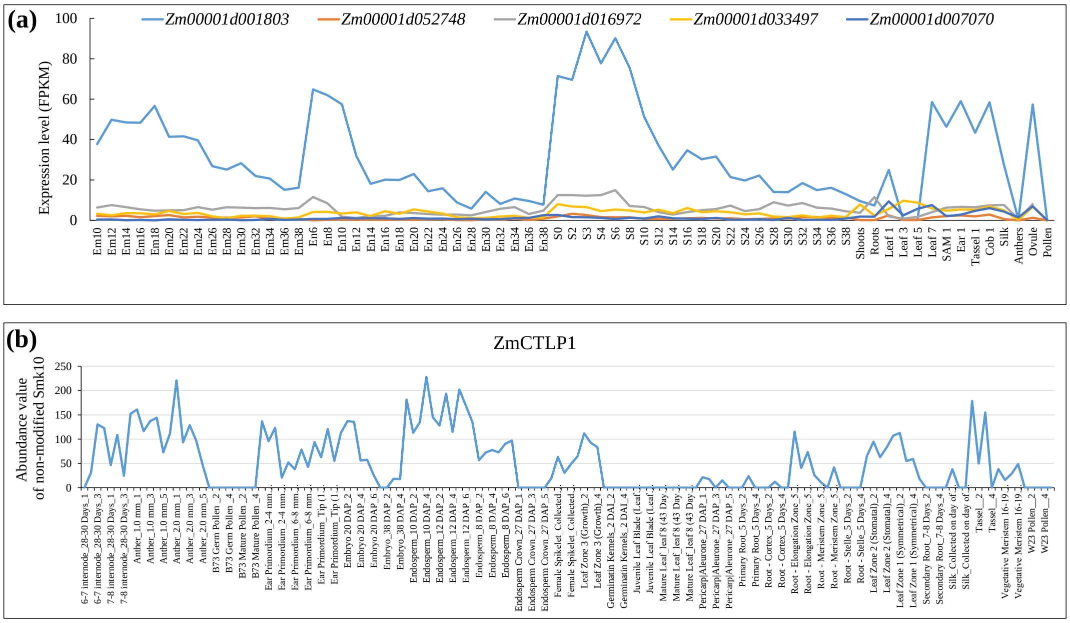

Fig. S14 Expression patterns of five maize genes (Chen *et al*., 2014) (a) and the abundance of ZmCTLP1 protein (Walley *et al*., 2016) (b) in various tissues. EM, embryo; EN, endosperm; s, seed.

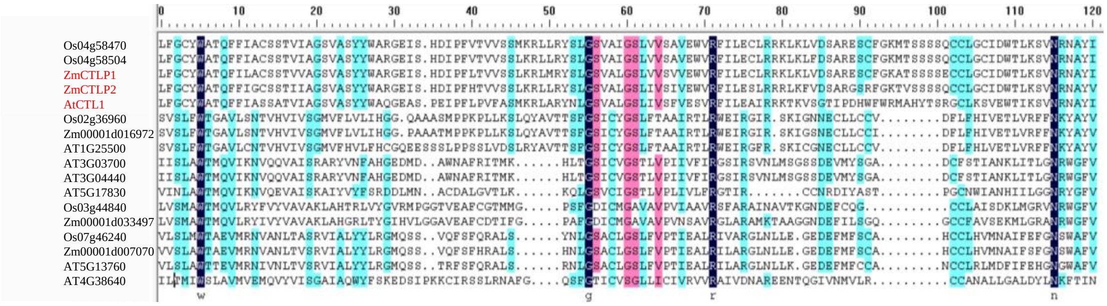

Fig. S15 Comparison of the conserved domains of CTLs in maize, *Arabidopsis thaliana*, and rice.

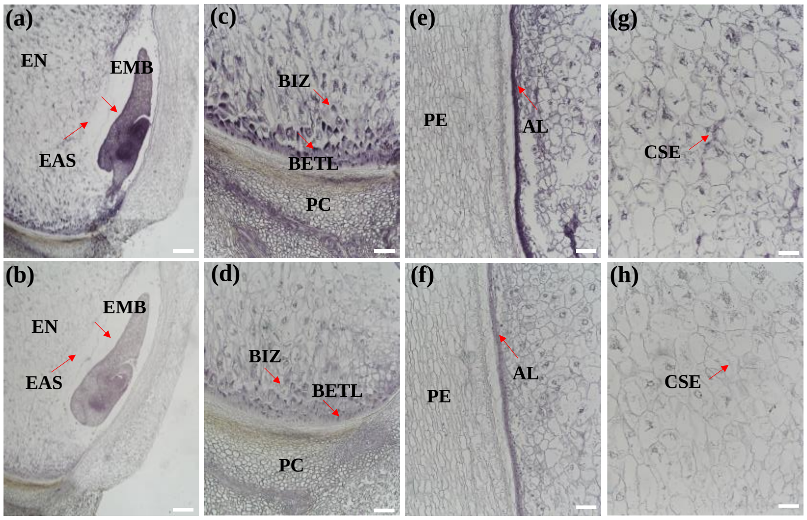

Fig. S16 *In situ* hybridization of *ZmCTLP1* using antisense probes in sections of wild-type kernels at 12 DAP. (a, c, e, g) *In situ* hybridization of *ZmCTLP1* using antisense probes in EMB and ESA (a), BETL, PC and BIZ (c), Al and PE (e) and CSE (g). (b, d, f, h) The *ZmCTLP1*-sense probe served as the negative control. EN, endosperm; EMB, embryo; BIZ, basal intermediate zone; BETL, basal endosperm transfer layer; PC, placento-chalazal region; PE, pericarp; AL, aleurone layer; CSE, central starchy endosperm; EAS, endosperm adjacent to scutellum. Bar = 100 μm for C–H and 250 μm for A and B.

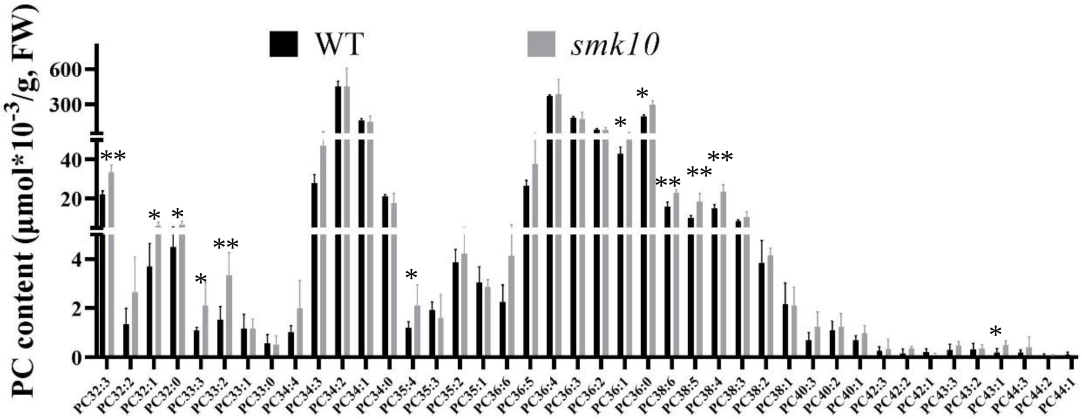

Fig. S17 Forty-two phosphatidylcholine species were identified in wild-type and *smk10* kernels. The means ± SE of five replicates per genotype are presented (**p < 0.01, *p < 0.05, Student's *t*-test).

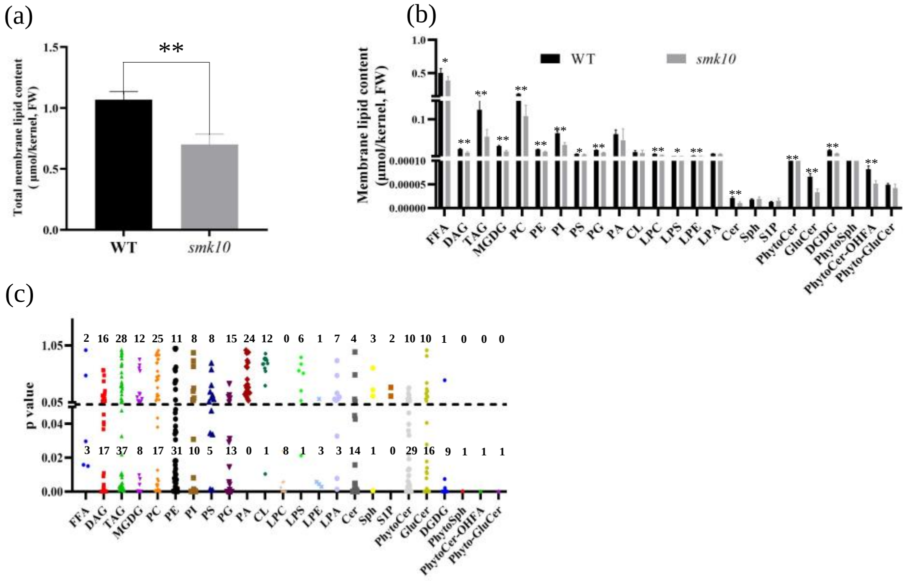

Fig. S18 Lipid contents per kernel between *smk10* and wild type at 12 DAP. The means ± SE of five replicates per genotype are presented (***p* < 0.01, **p* < 0.05, Student's *t*-test). (a) Lipid content per kernel in wild-type and *smk10*. (b) Twenty-four classes of membrane lipids per kernel were measured in wild-type and *smk10* kernels. (c) The contents of 423 lipid species in per kernel were measured. The y-axis shows the *p*-values associated with *t*-tests between the wild type and *smk10*. FFA, free fatty acids; DAG, diacylglycerols; TAG, triacylglycerols; MGDG, monogalactosyl diacylglycerols; PC, phosphatidylcholines; PE, phosphatidylethanolamines; PI, phosphatidylinositols; PS, phosphatidylserines; PG, phosphatidylglycerols; PA, phosphatidic acids; CL, cardiolipins; LPC, lyso-PC; LPS, lyso-PS; LPE, lyso-PE; LPA, lyso-PA; Cer, ceramides; Sph, sphingosines; S1P, sphingosine-1-phosphate; PhytoCer, phytoceramides; GluCer, glucosylceramides; DGDG, digalactosyl diacylglycerols; PhytoSph, phytosphingosines; PhytoCer-OHFA, phytoceramides with hydroxylated fatty acyls; Phyto-GluCer, phyto-glucosylceramides.

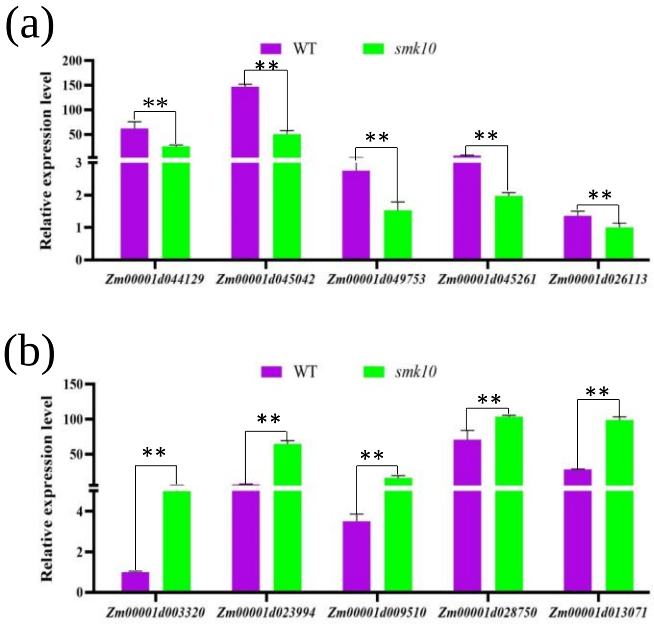

Fig. S19 qPCR confirmation of 10 selected DEGs at 12 DAP. The means ± SE of three replicates per genotype are presented in (a) and (b) (**$p < 0.01$, Student's t-test).

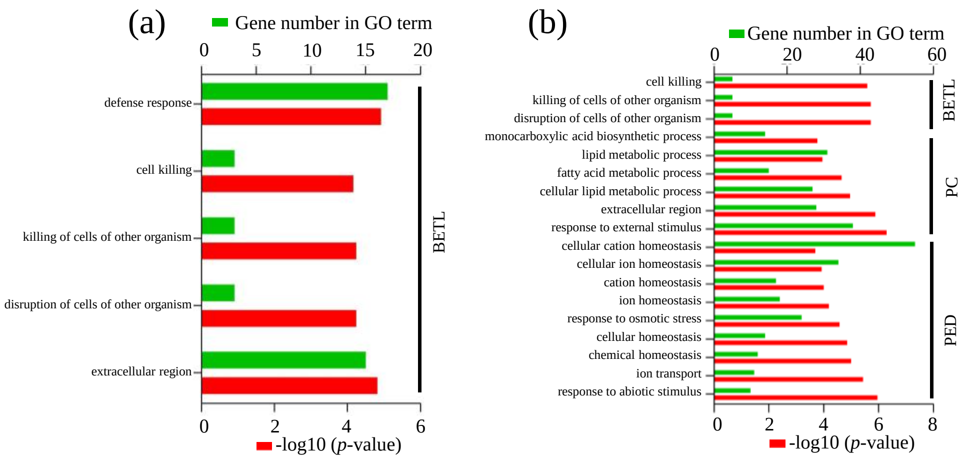

Fig. S20 Gene Ontology analysis of the DEGs between wild-type and *smk10* kernels at 6 and 12 DAP. The most significantly enriched GO terms in the BETL at 6 DAP (a) and in the BETL, PC, and PED at 12 DAP (b). Compartment-specific genes were selected based on publicly available data (Zhan *et al.*, 2015). Lower x-axis: $-\log_{10}$ (p-value); upper x-axis: Number of genes associated with a given GO term. BETL, basal endosperm transfer layer; PC, placento-chalazal region; PED, pedicel.

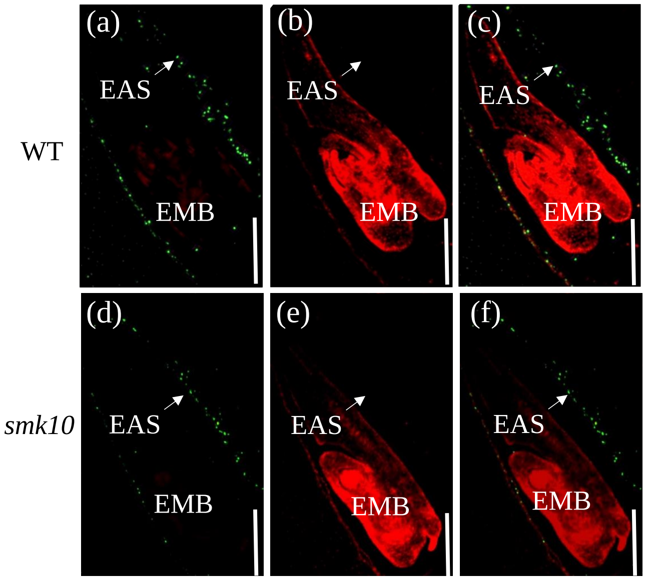

Fig. S21 Cell death detected by the TUNEL assay in 12 DAP wild-type (a–c) and *smk10* (d–f) kernels. EAS, endosperm adjacent to scutellum; EMB, embryo. Bar = 250 μm.

Table S1 The candidate genes predicted by MaizeGDB in the 300-kb smk10 interval.

Gene_ID	Gene Annotation	Expression in kernel
Zm00001d001790	Autophagy-related 4 variant 1	Yes
Zm00001d001791	Glucan endo-1,3-beta-glucosidase	No
Zm00001d001792	Uncharacterized conserved protein	No
Zm00001d001798	Uncharacterized protein	Yes
Zm00001d001799	GINS complex subunit 1 (GINS1, PSF1)	Yes
Zm00001d001800	Arylesterase / Paraoxonase	Yes
Zm00001d001803	SLC family 44 (choline transporter-like protein)	Yes
Zm00001d001804	MLO-LIKE PROTEIN 11-RELATED	Yes
Zm00001d001806	DNA mismatch repair protein MutS	Yes
Zm00001d001807	Uncharacterized protein	Yes

Table S2 Primers used in this study.

Primer name	Forward primer(5' to 3')	Reverse primer(5' to 3')
Primers were used for candidate gene analysis		
Zm00001d001790-seq1	CTCATTTCCTCTTGTTTACGTTACC	AATAAGTCCCAGGCCACATTACTG
Zm00001d001790-seq2	TATGCAAACCAATTTTAGGTTCATG	GCATTGCAAATGTAACATACCAACA
Zm00001d001798-seq1	TATTTTCTACAACTTCTGCGGTCTG	GAAAAAACGCACGAAATTCAAA
Zm00001d001799-seq1	TACTACTTTTCCATTGTCAGAGGC	CTTGAAGAACACTACAGAACTACCG
Zm00001d001807-seq	CAATTTGCTCCGCACCGAC	TTGAGTTTCCAAACTAGAGCTAAGCTAG
Zm00001d001806-seq1	CACCGTGCCGTGTTTTATAATG	GCTCTCAATGCAGCTACTCTGCTT
Zm00001d001806-seq2	AATGAAGCTGCATCTGTTCTTTCAC	GATTTCTGTACTGAAATGTCTGCGT
Zm00001d001804-seq1	TCGGTCGTGACTCGTGAGGAA	CGCCTTGGGTTGAAACTGAGAG
Zm00001d001804-seq2	AGAAAGCAAACCCAAAACTAACC	AACGAAGCCCCACAACAGTC
Zm00001d001804-seq3	ATGGTATTTTGTTTCAGGATCCAAC	AGCTCAGTCCTCAGTACGTCTGCA
Zm00001d001803-seq1	AGAAAGTGGCGGACCTCCTTT	CATTGCTTGTTGTTTCATTATTAGA
Zm00001d001803-seq2	TCCAATACATGTATGATGGGAATAA	ATATGTCTTGCAACAGATTGATTCA
Zm00001d001803-seq3	GTGGAATTCTCCCTGTTTTCCTGT	CAGGTGCAAGAATCTCAAGAGTC
Zm00001d001803-seq4	TTAGTTTTTTTTTGCCGTTTTAGTC	TGGTTAAATTTATGATGTTGGAAGT
Primers were used for amplification of the partly and full-length cDNA		
F, R	CGCCGTTCTCGTGCTCTTCGC	ATCATTACTGCTGTCATTAGGAT
DJ-1	AGGCACAGATCACTCGCTTCC	CAACGCCGTATCAGCAATAGCAC
Primers were used for qPCR and RNA in situ (Red bases indicated locked nucleic acid modification)		
ZmCTLP1-qPCR	TTGTATCTGTTGTTGAATGGGTG	TTTCGATTTACTGACTTGAGGGT
Actin	GATTCCTGGGATTGCCGAT	TCTGCTGCTGAAAAGTGCTGAG
Zm00001d044129	TAACCGCCATATTCATCGTACA	CATTTGTGTAGCCGCTAATACC
Zm00001d045042	TTTGACAAATGCAAGGCAGATC	GCTTCCAGGTGTACTTCTCATA
Zm00001d049753	GTCAACTTCGCCGTCTACTC	CATCGAACCTGTATCCGTAGAG
Zm00001d045261	GAACCTGAAGGGATTGCTGAAG	TCCAACCACAATCTCAGAATCT
Zm00001d026113	CAAGAAGAAGAGGACTATGCCC	CTGAAGTGCTTCTTGATCCCC
Zm00001d003320	TTGTTCCTCCATCTCTTCCG	AAGCAGGCCAAGATCACAG

Zm00001d023994	GAACGTTATGGCATTGTCTACG	CGATTTTTAAGCTGGCGTAAGA
Zm00001d009510	GCATGATTGATTCCTCTATCGC	AATTTCAGGTGAACGATTTCCC
Zm00001d028750	CGTGCTATTGTTACGTGTTGTA	ATGATCCAGACATACAGACACG
Zm00001d013071	CTGTCATGCATGAGAGTTTGAG	CTTGAACCATCACCTCCATCTC
RNA in situ		
Sweet4c	CATTGATAAAACAGTGCACAAGGCCATCGATTCTAG	CTAGAATCGATGGCCTTGTGCACTGTTTTATCAATG
ZmCTLP1	TAGCATGGAAACTTAGCGGCAGCCTAATGTTTTGTTACCCC	GGGGTAACAAAACATTAGGCTGCCGCTAAGTTTCCATGCTA
Primers were used for plasmid construction		
subcellular location	ACGCGTCGACATGGGAGGGCCCCTCGGCGCGAT	CGGGGTACCAGGCCCTTGAGTTAGTCTCTGC
pT7TS-ZmCTLP1	AGATCTATGGGAGGACCACTGGGAGCCAT	ACTAGTTTAAGGTCCCTGAGTCAGTCTCTGC
pT7TS-AtCTL1	AGATCTATGAGAGGACCTCTGGGAG	ACTAGTGTGAGTCAGGCTCTGC

Table S3 Chi-square tests of the mature kernel phenotypes in smk10 × CRISPR-generated mutants ears.

	Ear 1 (kernel number)	Ear 2 (kernel number)	Ear 3 (kernel number)		Ear 1 (kernel number)	Ear 2 (kernel number)	Ear 3 (kernel number)
WT	123	115	108	WT	133	145	131
Mutant$^{smk10 \times cas\text{-}1}$	105	131	124	Mutant$^{smk10 \times cas\text{-}2}$	113	122	119
χ^2	1.42	1.04	1.1	χ^2	1.62	1.98	0.93

Note: $\chi^2_{0.05} = 3.84$

Table S4 The contents of 423 lipid species in WT (B1-B5) and smk10 (A1-A5) kernels.

Species	A1	A2	A3	A4	A5	B1	B2	B3	B4	B5	Student's t-test
DAG32:2(16:1/16:1)	0.00622133	0.0052936	0.0054911	0.0064464	0.0044496	0.0045461	0.0047549	0.004079	0.003591	0.0033763	0.009281
DAG32:2(18:2/14:0)	0.00120668	0.0009244	0.0008538	0.0014675	0.0008238	0.0010029	0.0009679	0.0006224	0.00058	0.0007814	0.117256
DAG32:2(18:1/14:1)	0.000900088	0.0009926	0.0008621	0.0011942	0.0006478	0.0005783	0.0006375	0.000573	0.0005752	0.0006531	0.008253
DAG32:1(18:1/14:0)	0.000811046	0.0006617	0.0004939	0.0006882	0.0004013	0.0002855	0.0004071	0.0004991	0.0002851	0.0004024	0.022556
DAG33:2(15:0/18:2)	0.000227483	0.0002627	0.0001255	0.0001721	0.0001408	8.787E-05	0.000169	0.0001294	0.000174	0.0001108	0.13338
DAG33:1(17:1/16:0)	0.000811046	0.0007201	0.0006194	0.0007995	0.0005351	0.0005418	0.0003764	0.0005053	0.0005655	0.0004315	0.010151
DAG33:1(18:1/15:0)	0.000682449	0.0007006	0.0005608	0.0006275	0.0003309	0.0003514	0.0005223	0.0003943	0.000406	0.000449	0.065202
DAG34:4(18:3/16:1)	0.001622092	0.0018878	0.0014229	0.0015382	0.0011476	0.0010908	0.0011369	0.0007086	0.000754	0.0007698	0.003165
DAG34:3(18:3/16:0)	0.005449839	0.0067436	0.0044364	0.0058393	0.0037315	0.0041655	0.0045244	0.0040359	0.0027936	0.0034522	0.045134
DAG34:3(18:2/16:1)	0.000771491	0.0007493	0.0006529	0.0008805	0.0004647	0.0006149	0.0005992	0.0005915	0.0004108	0.0005773	0.104784
DAG34:2(18:1/16:1)	0.001740756	0.0016543	0.0011886	0.0021759	0.0013166	0.001186	0.001398	0.0012939	0.00116	0.001382	0.10356
DAG34:2(20:0/14:2)	0.038564372	0.0062473	0.0066713	0.0259883	0.0462	0.0051537	0.0066829	0.0045412	0.0108698	0.0048983	0.056397
DAG34:2(22:2/12:0)	0.001780311	0.0022381	0.0013895	0.0021353	0.0014574	0.0017277	0.0017898	0.0018732	0.0015418	0.0015628	0.596676
DAG34:1(15:1/19:0)	0.015617668	0.0026468	0.0024861	0.0102617	0.017904	0.0023646	0.0028268	0.0020272	0.0054905	0.0021751	0.070306
DAG34:1(16:0/18:1)	0.004727835	0.0094682	0.0048632	0.0096647	0.0076531	0.0100001	0.0107848	0.0119598	0.0116672	0.0131147	0.007634
DAG34:1(20:0/14:1)	0.00679496	0.001518	0.0016072	0.0054749	0.0079065	0.0013397	0.0011983	0.0011769	0.0022957	0.0012946	0.043869
DAG36:6(18:4/18:2)	0.000365924	0.0002238	0.0002093	0.0002631	0.0001197	0.0001317	0.000169	0.0001417	8.218E-05	0.0001808	0.060052

DAG36:5(18:4/18:1)	0.000454966	0.0003405	0.000226	0.0003542	0.0002535	0.0001903	0.0002228	0.0001848	0.000145	0.0001983	0.011892
DAG36:5(18:3/18:2)	0.001364899	0.0023354	0.0011886	0.0017811	0.00138	0.0016178	0.0018205	0.0020642	0.0011745	0.001831	0.728005
DAG36:4(18:3/18:1)	0.001829798	0.0022381	0.0014815	0.0022669	0.0018446	0.0028697	0.0024504	0.0031363	0.0023054	0.0027699	0.005917
DAG36:4(18:2/18:2)	0.037961031	0.051564	0.0267939	0.0513999	0.0390819	0.06369	0.0570044	0.069023	0.0492064	0.0654099	0.010145
DAG36:3(18:3/18:0)	0.000939643	0.0009926	0.001063	0.0008501	0.0006407	0.0008272	0.0008834	0.0007024	0.0006138	0.0007231	0.129468
DAG36:3(18:2/18:1)	0.008456695	0.0165426	0.0076171	0.0162427	0.0138065	0.0177819	0.0160697	0.0215905	0.0182983	0.0220832	0.017536
DAG36:2(18:2/18:0)	0.002621067	0.0041356	0.0025447	0.0040481	0.0036047	0.0037995	0.0035335	0.0041406	0.0042918	0.0039536	0.170039
DAG36:2(18:1/18:1)	0.007091708	0.0115506	0.006688	0.0155343	0.0150808	0.0194218	0.0161542	0.0221759	0.0208793	0.0282994	0.005959
DAG36:1(18:1/18:0)	0.00153305	0.0021505	0.0012723	0.0024085	0.002267	0.0021669	0.0019203	0.0026988	0.0029482	0.0030556	0.078357
DAG37:3(20:3/17:0)	0.002947436	0.0033085	0.0024024	0.0032789	0.0022318	0.002306	0.002412	0.0019471	0.0015224	0.0018952	0.017187
DAG32:0(16:0/16:0)	0.011628538	0.0059195	0.0049015	0.0086167	0.0047173	0.0053501	0.004424	0.0057397	0.0147724	0.0071204	0.890791
DAG32:0(18:0/14:0)	0.000433343	0.0003131	0.0006058	0.0007793	0.0004178	0.0004262	0.0003183	0.0003268	0.001095	0.0003956	0.988411
DAG34:0(16:0/18:0)	0.006876004	0.0049376	0.0051218	0.006911	0.0042994	0.0046995	0.0035859	0.0039729	0.015986	0.0048531	0.69227
DAG34:0(22:0/12:0)	0.000707852	0.0005834	0.0006196	0.0007058	0.0003079	0.000415	0.0003076	0.0004698	0.0006208	0.000386	0.145928
DAG36:0(18:0/18:0)	0.010805186	0.0086515	0.0123087	0.0110429	0.0098963	0.0080194	0.0062063	0.0057193	0.0198221	0.0082879	0.736734
DAG36:0(22:0/14:0)	0.000664518	0.0004127	0.0005507	0.0006617	0.0004728	0.0003029	0.0004137	0.0004187	0.0006835	0.0003763	0.202725
LPC14:0	0.035611398	0.0331415	0.0357701	0.0393691	0.0291092	0.0278387	0.0263344	0.0236504	0.0214889	0.0219512	0.001141
LPC15:0	0.001043803	0.0009283	0.0010738	0.0010703	0.0009001	0.000755	0.000823	0.0005677	0.0007538	0.0005704	0.001332
LPC16:1	0.000117521	0.0001196	0.0001168	0.0001536	0.0001082	0.0001371	9.504E-05	8.527E-05	0.000142	0.0001014	0.452057
LPC16:0	0.00394729	0.0070924	0.0048828	0.0067164	0.0045438	0.0099554	0.0102187	0.0158462	0.0172371	0.0154538	0.000985
LPC18:3	0.000808673	0.0012729	0.0008402	0.0013902	0.0007362	0.0012025	0.0012332	0.0015236	0.0017477	0.0018075	0.02764
LPC18:2	0.006615646	0.0116953	0.0079874	0.0116333	0.0073337	0.0120947	0.0111286	0.0146749	0.0168679	0.0154563	0.011201
LPC18:1	0.004128977	0.0053273	0.0044194	0.0058934	0.0041973	0.0052182	0.0051718	0.0062155	0.006774	0.0065506	0.038915
LPC18:0	0.00083724	0.0009705	0.0009532	0.0012239	0.0008196	0.0011298	0.0010404	0.0015662	0.0023751	0.0017543	0.040672
PC32:3	0.032907003	0.0328576	0.033892	0.0390724	0.028199	0.0238397	0.0222203	0.0194847	0.0237032	0.0219024	0.000377
PC32:2	0.002163463	0.0049008	0.0015464	0.0031612	0.0014376	0.0023339	0.0007256	0.0009673	0.0010741	0.0016409	0.103524
PC32:1	0.005123909	0.0089105	0.0042526	0.0068282	0.0054187	0.0026674	0.0048975	0.0035929	0.0030076	0.0043519	0.030083
PC32:0	0.007401201	0.0082422	0.0074743	0.0063224	0.0042022	0.0045845	0.0047162	0.0031783	0.0040818	0.0058502	0.026266
PC33:3	0.001138646	0.0024504	0.0034794	0.0021496	0.001327	0.0010003	0.0010883	0.0012437	0.0012174	0.0009274	0.044453
PC33:2	0.002277293	0.0045667	0.0032217	0.0039199	0.0027646	0.001167	0.0008166	0.0020037	0.0017187	0.0019976	0.004929
PC33:1	0.001480223	0.001448	0.0005155	0.0010116	0.001327	0.0009169	0.0021767	0.0011746	0.0006445	0.0008561	0.993597
PC33:0	0.001138646	0.0003341	0.0005155	0.0003793	0.0002212	0.001167	0.0002721	0.0006218	0.0003581	0.0004281	0.826416
PC34:4	0.001024817	0.0018934	0.0021907	0.0037934	0.0011059	0.0013337	0.001179	0.0010364	0.0009309	0.0006421	0.093929
PC34:3	0.032679256	0.0709502	0.033892	0.0637298	0.0336176	0.0317584	0.0298386	0.0311617	0.0249205	0.0218311	0.057748
PC34:2	0.354006153	0.676533	0.3072183	0.5497954	0.3593989	0.4816285	0.4505727	0.5021114	0.4284474	0.3882516	0.991418
PC34:1	0.121038441	0.2225409	0.1094079	0.1767741	0.1218638	0.1583756	0.1649742	0.1855884	0.1555386	0.1546728	0.560944
PC34:0	0.014460826	0.023613	0.0119846	0.0217491	0.0164771	0.0204221	0.0203157	0.0219721	0.0221993	0.0203329	0.167092
PC35:4	0.001252476	0.0031187	0.0028351	0.0020231	0.001327	0.0015838	0.000907	0.0011055	0.0010741	0.0012842	0.049375
PC35:3	0.00170797	0.0017821	0.0006443	0.0030348	0.0008847	0.0015838	0.0019046	0.0016583	0.0023632	0.0021403	0.493187
PC35:2	0.003757515	0.0051236	0.0045103	0.004805	0.0028752	0.0032509	0.0042626	0.0044912	0.0035089	0.0038526	0.483931
PC35:1	0.00330211	0.0030073	0.0025773	0.0027819	0.002654	0.0027507	0.0038999	0.0026947	0.0035089	0.002283	0.626999
PC36:6	0.00284661	0.008019	0.004252	0.003667	0.00188	0.002917	0.002902	0.002211	0.001289	0.001926	0.123566

	6	5	6			4	2			3	
PC36:5	0.028693994	0.065047	0.0278352	0.04236	0.024218	0.0309249	0.0233086	0.0270852	0.0244193	0.0264685	0.180651
PC36:4	0.300831263	0.5693838	0.2899501	0.4638108	0.2874085	0.3655142	0.3631427	0.3671694	0.3624938	0.3878949	0.826538
PC36:3	0.124226721	0.2502749	0.1246142	0.2217896	0.1505052	0.1811317	0.1674229	0.1987164	0.1820347	0.1890604	0.732696
PC36:2	0.056932493	0.1139436	0.0699746	0.0848465	0.0726538	0.0831055	0.0666608	0.0896159	0.0938817	0.0761949	0.842103
PC36:1	0.042130003	0.0568047	0.0529642	0.0603157	0.0498735	0.0440951	0.0380919	0.0478136	0.0418923	0.0428061	0.0265
PC36:0	0.28454855	0.3074137	0.3052853	0.3372365	0.2498098	0.2057216	0.2127704	0.1994074	0.1834669	0.1792864	0.000216
PC38:6	0.020381805	0.023613	0.0243559	0.0244044	0.0218957	0.0172546	0.0170506	0.0143717	0.0126035	0.018264	0.000669
PC38:5	0.01332218	0.0157049	0.0211341	0.0240251	0.0174723	0.0107529	0.0107927	0.0100187	0.0105268	0.0079191	0.003012
PC38:4	0.022431439	0.023613	0.0206187	0.0295888	0.0215639	0.0176714	0.0131508	0.0140953	0.0135344	0.016409	0.001468
PC38:3	0.008198271	0.0147024	0.0097939	0.0112539	0.0088468	0.0080855	0.0090695	0.0089823	0.0075908	0.0086325	0.117502
PC38:2	0.003871433	0.0042325	0.0042526	0.0045522	0.0038704	0.0039177	0.0022673	0.004353	0.0043682	0.0043519	0.494795
PC38:1	0.001138646	0.0027846	0.0023196	0.0015174	0.0027646	0.0029174	0.0021767	0.0010364	0.0030793	0.0015696	0.923313
PC40:3	0.001821799	0.0015593	0.0016752	0.0006322	0.0005529	0.0005835	0.001179	0.0008292	0.0005013	0.0003567	0.106647
PC40:2	0.001480223	0.0020049	0.0006443	0.0012645	0.0008847	0.001417	0.000907	0.0011055	0.0015038	0.0005707	0.610055
PC40:1	0.0009109	0.0013366	0.0007732	0.0012645	0.0006635	0.0007502	0.0007256	0.0008292	0.0007877	0.0003567	0.094523
PC42:3	0.000113829	0.0001113	0.0010309	0.0002529	0.0002212	0.0002501	9.073E-05	0.0001382	0.0005013	0.0003567	0.688035
PC42:2	0.000341576	0.0003341	0.0002578	0.0005058	0.0003318	0.0001667	0	0	0.0002148	0.0004281	0.063423
PC42:1	0.000113829	0.0002228	0	0	0	0.0001667	0.0004534	0.0001382	7.161E-05	0.000214	0.11179
PC43:3	0.000455494	0.0004456	0.0002578	0.0006322	0.0006635	0.0004168	9.073E-05	0.0002073	0.0006445	0.0001427	0.168587
PC43:2	0.000113829	0.0004456	0.0005155	0.0003793	0.0003318	0.0002501	0.0006348	0	0.0002148	0.0004994	0.782648
PC43:1	0.000569323	0.0003341	0.0007732	0.0003793	0.0004423	8.338E-05	0.0003628	0.0002073	0	0.0003567	0.023987
PC44:3	0.000227747	0.0001113	0.0002578	0.0011381	0.0003318	0.0002501	0.0003628	6.907E-05	0.0001432	0.0001427	0.284508
PC44:2	0	0.0001113	0	0.0001264	0	0	0	0	0.0001432	0.0001427	0.837933
PC44:1	0.000113829	0.0001113	0	0.0001264	0.0001106	8.338E-05	0	0.0002073	0.0002148	7.133E-05	0.643443
LPE16:0	0.0026339	0.0054273	0.0033159	0.0048345	0.0024302	0.0027507	0.0031552	0.0043051	0.0046734	0.0043024	0.880951
LPE18:1	0.008719162	0.0108606	0.0110325	0.0133342	0.0079256	0.0078205	0.0077207	0.0072827	0.0079822	0.0073662	0.021581
LPE18:2	0.0033908	0.0061049	0.0041279	0.0060754	0.0037553	0.0039033	0.0035703	0.0046441	0.0054871	0.0053314	0.885245
LPE18:3	0.00020586	0.0003751	0.0003037	0.0004088	0.00025	0.000202	0.0001887	0.0002532	0.0002437	0.0001723	0.045466
PE32:3	6.28479E-05	8.467E-05	5.86E-05	8.654E-05	6.9E-05	4.687E-05	5.694E-05	4.785E-05	2.733E-05	4.867E-05	0.007212
PE32:2	0.000198213	0.0003152	0.0002622	0.0002813	0.0001794	0.0001094	0.000114	0.0001203	0.0001468	0.0001091	0.001329
PE32:1	8.46469E-05	0.0001482	0.0001171	0.000138	9.071E-05	0.0001094	9.49E-05	6.816E-05	0.0001075	9.734E-05	0.201197
PE32:0	9.43159E-05	7.055E-05	9.675E-05	6.224E-05	5.522E-05	5.381E-05	8.732E-05	7.392E-05	7.34E-05	5.704E-05	0.537244
PE33:3	0.000217638	0.0002987	0.0002419	0.0003787	0.0002011	0.0001372	0.0001519	0.0001131	0.0002014	0.0001762	0.014193
PE33:2	0.000246645	0.0003505	0.0002317	0.0003598	0.0001715	0.000264	0.000226	0.0001827	0.0001861	0.0001829	0.146926
PE33:1	8.21858E-05	9.643E-05	9.931E-05	0.0001217	4.532E-05	7.643E-05	8.927E-05	8.846E-05	5.293E-05	7.554E-05	0.406518
PE34:4	8.70202E-05	0.0001293	0.0001171	8.929E-05	8.279E-05	3.822E-05	7.022E-05	5.219E-05	8.021E-05	5.032E-05	0.007084
PE34:3	0.003989306	0.0062259	0.003926	0.0051236	0.0030538	0.0028416	0.0026163	0.0023573	0.002656	0.0022807	0.009062
PE34:2	0.042968737	0.0785747	0.0469801	0.0637545	0.0449272	0.0485667	0.0429829	0.0467158	0.052958	0.0488235	0.322687
PE34:1	0.005386727	0.0095895	0.0057185	0.0079748	0.0058337	0.007104	0.0062769	0.0071777	0.0091321	0.0077584	0.548541
PE34:0	0.000350542	0.0005057	0.0003718	0.000541	0.00042	0.0004933	0.0004518	0.0004349	0.0005906	0.0004749	0.29733
PE35:4	8.21858E-05	0.0001106	0.0001044	8.39E-05	4.532E-05	7.643E-05	9.873E-05	5.8E-05	8.367E-05	6.712E-05	0.544074

PE35:3	0.000116027	0.0001905	0.0001222	0.0002083	7.692E-05	9.55E-05	9.49E-05	0.0001073	6.827E-05	7.89E-05	0.067658
PE35:2	0.000420686	0.0005998	0.0004812	0.0006492	0.0004357	0.0003178	0.0003968	0.0002957	0.0004353	0.0003524	0.016533
PE35:1	0.001025081	0.0011196	0.0011152	0.0012011	0.0009128	0.0008319	0.0009493	0.0006944	0.0007835	0.0006528	0.003579
PE36:6	0.000415851	0.0006304	0.0003718	0.0006005	0.0003411	0.0003491	0.0003512	0.0002247	0.0003107	0.0002249	0.026705
PE36:5	0.003002813	0.0051534	0.0031953	0.0038143	0.0023973	0.0026905	0.00236	0.0021935	0.0029922	0.0025526	0.086178
PE36:4	0.033372077	0.0556918	0.0324571	0.0440077	0.0312566	0.0402401	0.0355241	0.0356079	0.0442297	0.0400355	0.96418
PE36:3	0.006496455	0.0109443	0.0065408	0.0093951	0.0069554	0.009249	0.0082591	0.0092422	0.0113614	0.010588	0.150076
PE36:2	0.005265866	0.006513	0.0051787	0.0067466	0.0051081	0.0046254	0.0048131	0.0045957	0.0055253	0.004647	0.049138
PE36:1	0.05701213	0.0598133	0.0655181	0.0681933	0.0531637	0.0460907	0.0491528	0.0370495	0.0400137	0.0370876	0.000902
PE37:2	0.000268356	0.0002517	0.0002317	0.0002813	0.000211	0.0002136	0.0001538	0.0001682	0.0002117	0.0001494	0.00609
PE37:1	0.00025148	0.0002281	0.0002317	0.0002462	0.0001715	0.0001946	0.0001462	0.0001261	0.0001946	0.000146	0.012247
PE38:6	0.00046903	0.0005104	0.0004201	0.0005248	0.0004298	0.0003613	0.0004424	0.0002972	0.0003516	0.0003256	0.007114
PE38:5	0.000222473	0.0002094	0.0001935	0.0002164	0.0001518	0.0001424	0.0001633	0.0001595	0.0001707	0.0001242	0.015282
PE38:4	0.000964694	0.0011784	0.0010439	0.0010496	0.0007393	0.0007191	0.0008373	0.0006031	0.0006725	0.0006578	0.006996
PE38:3	0.003522649	0.004055	0.0034524	0.0041578	0.0030144	0.0027773	0.0027284	0.0023544	0.0025177	0.0024687	0.001407
PE38:2	0.001259684	0.0018534	0.0012501	0.001615	0.0012992	0.0010804	0.0011524	0.0009018	0.0011129	0.001022	0.013775
PE38:1	9.66891E-05	0.0002211	0.0001273	0.0002056	0.0001104	0.0001077	0.0001158	0.0001305	0.0001707	0.0001158	0.412322
PE40:3	0.000193378	0.0003411	0.0002317	0.0002218	0.0001637	0.0001615	0.0001557	0.0001145	0.0001007	8.562E-05	0.013194
PE40:2	0.000921184	0.0013972	0.0007817	0.001074	0.0006821	0.0006618	0.0006512	0.0007089	0.0007766	0.0006965	0.064852
PE40:1	0.000502871	0.0005033	0.0005321	0.0004815	0.000483	0.0003578	0.0003209	0.0002697	0.0003516	0.0003524	1.86E-05
PE42:3	0.000140199	0.0002117	0.0001783	0.0001947	0.0001085	0.0001112	9.873E-05	8.264E-05	8.194E-05	7.218E-05	0.004762
PE42:2	0.001283856	0.0021286	0.001217	0.0016339	0.0011238	0.0012818	0.0010404	0.0011424	0.0019886	0.001361	0.656292
PE42:1	0.000244184	0.0003246	0.0002622	0.0002678	0.0002642	0.0002814	0.0002544	0.0002653	0.0004609	0.0003474	0.260031
PE43:3	1.45034E-05	3.764E-05	4.071E-05	8.929E-05	2.757E-05	2.255E-05	1.328E-05	3.188E-05	2.22E-05	2.18E-05	0.17088
PE43:2	0.000171667	0.0003457	0.0001986	0.0003246	0.0002188	0.0001893	0.0001671	0.0002435	0.0002339	0.000287	0.516197
PE43:1	5.56402E-05	7.055E-05	7.128E-05	4.871E-05	4.532E-05	1.907E-05	5.124E-05	4.494E-05	5.634E-05	6.882E-05	0.330588
PE44:3	0.000321535	0.0003811	0.0003641	0.0004382	0.0002839	0.0002988	0.0002374	0.0001972	0.0002953	0.0002366	0.012487
PE44:2	0.001003369	0.0010585	0.0009013	0.001074	0.0009522	0.0008771	0.0008715	0.0006524	0.0008893	0.0008945	0.021517
PE44:1	0.00090668	0.0007385	0.0008682	0.0009225	0.0007886	0.0006097	0.000617	0.0004002	0.0006708	0.0005689	0.001601
PG32:3	5.59039E-05	5.028E-05	6.854E-05	5.085E-05	4.044E-05	3.379E-05	3.365E-05	1.951E-05	3.175E-05	4.77E-05	0.014413
PG32:2	0.000146792	0.0002011	0.0001295	0.000127	8.66E-05	0.0001127	8.419E-05	0.0001072	0.0001799	0.0001008	0.416639
PG32:1	0.000132816	0.0002729	0.0001676	0.000127	0.0002136	0.0002141	0.0001796	0.0001998	0.0001852	0.0002811	0.400528
PG32:0	0.00432719	0.0077209	0.0043339	0.0075119	0.0056351	0.0119723	0.0110536	0.0120026	0.0124695	0.0145526	0.000123
PG33:2	0.000132816	0.0001795	0.00016	0.000127	5.194E-05	0.0001183	9.539E-05	8.287E-05	0.0001482	5.835E-05	0.297822
PG33:1	7.69118E-05	5.028E-05	3.048E-05	8.471E-05	2.308E-05	4.51E-05	4.491E-05	9.747E-05	4.762E-05	6.364E-05	0.685034
PG34:4	0.000139848	0.0001221	0.0002133	0.0002626	7.51E-05	0.000107	0.000129	0.0001316	8.999E-05	0.000122	0.211432
PG34:3	0.00334852	0.0054154	0.0039987	0.0042091	0.0025866	0.0038368	0.0031029	0.003153	0.003038	0.0029434	0.19839
PG34:2	0.027543129	0.0459516	0.028768	0.0366026	0.0271711	0.0440695	0.0382388	0.0416024	0.0387633	0.0383438	0.102701
PG34:1	0.006571169	0.0112257	0.0060933	0.009265	0.0081236	0.0149134	0.0135561	0.0161839	0.0147082	0.0162338	0.000191
PG34:0	0.000887782	0.0014077	0.0006094	0.0015498	0.0016513	0.002924	0.0025698	0.0035428	0.0040541	0.0038609	0.000239
PG36:6	8.38558E-05	0.0001149	3.806E-05	2.542E-05	8.66E-05	6.199E-05	5.048E-05	5.846E-05	9.525E-05	0.0001008	0.857671
PG36:5	0.00025165	0.000538	0.000365	0.000372	0.000213	0.000219	0.000314	0.000282	0.000190	0.000249	0.149468

	5	7	6	6	6	8	2	7	6	3	
PG36:4	0.001810548	0.0028944	0.0022012	0.0028286	0.0018476	0.0025354	0.0021378	0.0027485	0.0027575	0.0025191	0.413463
PG36:3	0.000873806	0.001774	0.0013405	0.0012873	0.0011663	0.001217	0.0013354	0.0016081	0.0014978	0.0013471	0.50382
PG36:2	0.001349165	0.0018817	0.0013939	0.0016176	0.0013222	0.001555	0.001341	0.0012573	0.0013972	0.0014584	0.371593
PG36:1	0.013946352	0.0152693	0.0170156	0.016345	0.0115532	0.0109244	0.0111378	0.0092493	0.0099978	0.0107607	0.002675
PG36:0	0.093807442	0.0945462	0.1058863	0.1096469	0.075474	0.0699636	0.0726565	0.0619382	0.0611777	0.0576642	0.001498
PI32:3	0.00045523	0.0004995	0.0002699	0.0006305	0.0003112	0.0003219	0.0002314	0.0001306	0.0001022	0.0003453	0.034911
PI32:2	0.000995722	0.0012356	0.0005668	0.0006936	0.0010004	0.0006008	0.0004206	0.0006901	0.0007767	0.0004468	0.053716
PI32:1	0.000426751	0.0006573	0.0004858	0.0008197	0.0007113	0.000515	0.0002314	0.0005409	0.000511	0.0006093	0.191964
PI32:0	0.003840668	0.0047848	0.0018623	0.0049177	0.004935	0.0053644	0.0058258	0.0065656	0.0081759	0.0082662	0.010666
PI33:3	0.000341401	0.0005784	0.0002969	0.0005675	0.0001556	0.000279	4.206E-05	9.325E-05	0.0002657	0.000264	0.071107
PI33:2	0.000768151	0.0013934	0.0006748	0.0012609	0.0006446	0.0009656	0.0007782	0.0009326	0.0003679	0.0007921	0.368524
PI34:4	0.0001707	0.0004995	0.0002699	0.0003153	0.0001778	0.0002575	0.0002524	0.0004477	0.0003475	0.0003047	0.626738
PI34:3	0.044039701	0.0645426	0.0409164	0.0510997	0.0364349	0.0309202	0.0261428	0.0285563	0.030537	0.0283936	0.005916
PI34:2	0.285546909	0.4307132	0.2514903	0.3981737	0.2825425	0.3612996	0.3156058	0.3902875	0.4225195	0.4155442	0.240718
PI34:1	0.037923059	0.0611774	0.0321718	0.0560173	0.0409031	0.0572484	0.0521593	0.0631932	0.0733379	0.0692983	0.032547
PI34:0	0.001963053	0.0028919	0.0017273	0.0030893	0.0023341	0.0035405	0.0034493	0.0044206	0.0048442	0.0042245	0.001872
PI35:3	0.000938851	0.0009727	0.0008906	0.0006936	0.0006891	0.0004935	0.0004206	0.0005036	0.0006949	0.0007718	0.020426
PI36:6	0.000967243	0.0017088	0.0010796	0.0009772	0.0005335	0.0004292	0.0004838	0.0006155	0.0006949	0.0003453	0.026704
PI36:5	0.003499268	0.0050215	0.0033197	0.0046024	0.0029343	0.0026607	0.0022714	0.0028538	0.0029024	0.0029247	0.024468
PI36:4	0.027055377	0.0372533	0.0255862	0.034865	0.0226746	0.0265858	0.0202117	0.0253668	0.027287	0.0257127	0.184448
PI36:3	0.01931717	0.0298394	0.0199184	0.0306724	0.0225856	0.0217149	0.0215999	0.0253855	0.0262242	0.0285561	0.936178
PI36:2	0.015561852	0.0228462	0.0141156	0.0235481	0.0176728	0.0188611	0.0166153	0.0218043	0.0248139	0.0211226	0.444486
PI36:1	0.006201992	0.0085707	0.0084478	0.0084168	0.0070692	0.0065445	0.0055945	0.0062298	0.0076036	0.006154	0.052156
MGDG32:5(18:2/14:3)	0.007717902	0.0082136	0.0068346	0.0105484	0.007088	0.0043928	0.0047495	0.0063695	0.0034643	0.0043325	0.003038
MGDG32:4(18:2/14:2)	0.007166598	0.0201366	0.0177078	0.0102188	0.011577	0.0087855	0.0110103	0.0090234	0.0102004	0.0090416	0.164232
MGDG32:3(18:1/14:2)	0.003307647	0.0047692	0.0068346	0.0102188	0.0082693	0.0032468	0.0075561	0.0067233	0.0042341	0.0047091	0.371742
MGDG32:3(18:2/14:1)	0.001378172	0.0031794	0.000932	0.0019779	0.0009451	0.0003819	0.001943	0.002654	0.0019246	0.0026372	0.7118
MGDG32:2(18:1/14:1)	0.004134603	0.0052991	0.0006213	0.0019779	0.0025989	0.000764	0.0008636	0.002477	0.0013472	0.0016953	0.125859
MGDG32:2(18:2/14:0)	0.000275652	0	0	0	0	0.000191	0.0006477	0.0003538	0.0001925	0	0.105331
MGDG34:3(18:2/16:1)	0.001653824	0.0005299	0.0003107	0.0006593	0.0004725	0.000764	0.0012953	0.0010616	0.0009623	0.0009418	0.303022
MGDG34:3(18:3/16:0)	0.00110252	0.0018547	0.0021746	0.0019779	0.0028352	0.0028649	0.0032383	0.0033617	0.002502	0.0024488	0.028385
MGDG34:2(18:1/16:1)	0.001378172	0.0007949	0.000932	0.0023075	0.0014176	0.000764	0.0008636	0.0001769	0.0009623	0.0015069	0.170755
MGDG34:2(16:0/18:2)	0.00854477	0.0148375	0.0055919	0.0121967	0.0063792	0.0114593	0.0196459	0.0198161	0.0150119	0.0178949	0.015374
MGDG34:1(18:1/16:0)	0.003307647	0.0039743	0.001864	0.0052742	0.0018901	0.0038198	0.0032383	0.0021232	0.0038492	0.0039557	0.858219
MGDG36:6(18:2/18:4)	0.012403721	0.0249057	0.0201931	0.0158227	0.010632	0.0137512	0.0133851	0.009908	0.0115476	0.012809	0.132805
MGDG36:6(18:3/18:3)	0.047409933	0.0773667	0.0571619	0.0754873	0.0389839	0.0372429	0.0392917	0.0318473	0.0433036	0.0382385	0.025767
MGDG36:6(15:0/21:6)	0.001378172	0.0007949	0.0021746	0.0013186	0.0007088	0.000573	0.0008636	0.0007077	0.0003849	0.0005651	0.043472
MGDG36:5(18:2/18:3)	0.013781981	0.0368288	0.0192611	0.0260414	0.0132308	0.0204359	0.0196459	0.0205238	0.0240575	0.0188367	0.80801
MGDG36:5(18:4/18:1)	0.002205127	0.0021197	0	0.0023075	0.0007088	0.0013369	0.0010794	0.0014154	0.0013472	0.0007535	0.577301
MGDG36:4(18:2/18:2)	0.02480753	0.0548456	0.0260956	0.0560386	0.0359124	0.0779237	0.0734022	0.1127042	0.0894941	0.0875906	0.000966
MGDG36:4(18:3/18:1)	0.006890946	0.006094	0.0052813	0.0112077	0.0047253	0.0091675	0.0112262	0.0086695	0.0073135	0.00923	0.120361

MGDG36:3(18:0/18:3)	0.012403721	0.0113931	0.0136692	0.0098891	0.0122858	0.0074486	0.0084197	0.007431	0.0144346	0.00923	0.117743
MGDG36:3(18:2/18:1)	0.006615294	0.0132477	0.0065239	0.0141744	0.0066155	0.0166161	0.0235319	0.0291934	0.0286766	0.0210971	0.001211
DGDG34:4(18:3/16:1)	0.000170349	0.0001965	0.0001931	0.0001582	0.0001284	0.0001556	8.851E-05	6.816E-05	6.254E-05	0.0001809	0.063913
DGDG34:3(18:3/16:0)	0.006621623	0.0097623	0.0055038	0.0097437	0.0062563	0.0076849	0.0061343	0.0065821	0.0073782	0.0058674	0.408102
DGDG34:3(16:1/18:2)	0.000159713	0.000273	0.0002534	0.0003296	0.0001835	0.000268	0.0002213	0.00025	0.000268	0.0003067	0.515886
DGDG34:2(18:2/16:0)	0.03607823	0.0592837	0.0362331	0.0553243	0.039877	0.0511837	0.0422581	0.0448253	0.0515671	0.0458064	0.746672
DGDG34:1(16:0/18:1)	0.017320539	0.0260001	0.0159682	0.0262778	0.0185212	0.0262963	0.0202438	0.0239654	0.0277353	0.023383	0.207598
DGDG36:6(18:2/18:4)	0.001096543	0.0018891	0.0011104	0.0014239	0.0012843	0.0013313	0.0011861	0.0013331	0.0012327	0.0013371	0.618974
DGDG36:6(18:3/18:3)	0.010943803	0.0195901	0.0122869	0.015967	0.0113935	0.0136322	0.0096484	0.0112555	0.0133898	0.0106336	0.237587
DGDG36:4(18:2/18:2)	0.016266628	0.0234667	0.0144956	0.0227837	0.0197963	0.0436284	0.0357609	0.0426287	0.0465203	0.043203	1.6E-05
DGDG36:4(18:3/18:1)	0.003321447	0.0052852	0.0034037	0.0048653	0.0044858	0.0078664	0.0063467	0.0073547	0.0080749	0.0077393	0.000201
DGDG36:2(18:0/18:2)	0.005684793	0.0068249	0.0050814	0.0074231	0.0053665	0.0070625	0.0070283	0.0074229	0.0087806	0.0085887	0.01994
TAG38:4(18:3)	0.000538295	0.0007228	0.0006157	0.0006247	0.0004195	0.0004044	0.0005084	0.0003765	0.0004228	0.0004443	0.024308
TAG38:3(18:3)	0.000672341	0.0007144	0.0006114	0.0006157	0.0005185	0.0004044	0.000505	0.0004931	0.0003973	0.0004428	0.002066
TAG39:4(18:3)	0.000498037	0.0006705	0.000464	0.0007379	0.0003567	0.0004028	0.0004395	0.0004503	0.0002878	0.0003143	0.062974
TAG39:3(18:3)	0.000932874	0.0011418	0.0008885	0.0012993	0.0007401	0.0005288	0.0005067	0.0005846	0.0004941	0.0005579	0.001614
TAG43:0(15:0)	0.000616789	0.0005175	0.0003848	0.0006088	0.0002781	0.0002996	0.0002792	0.0002022	0.0014493	0.0002922	0.926823
TAG45:0(15:0)	0.000959684	0.0003625	0.000442	0.0009597	0.0002388	0.000239	0.0002481	0.0001653	0.0005527	0.000335	0.12741
TAG48:2(18:1)	0.000275828	0.0001006	0.0001341	0.0004074	8.644E-05	9.005E-05	7.926E-05	8.561E-05	0.0001567	0.0001579	0.211961
TAG48:1(18:1)	0.000561236	0.0002996	0.0002375	0.0007357	0.0002074	0.0001719	0.0001982	0.0002097	0.0003413	0.0003069	0.170871
TAG48:0(16:0)	0.006344213	0.0015252	0.0012754	0.0036171	0.0010181	0.0009758	0.0010874	0.0007972	0.0038347	0.0022093	0.424389
TAG49:3(18:2)	0.00033182	0.0003062	0.0003152	0.0004424	0.0001585	0.0001572	0.0001693	0.0001402	0.0001692	0.0001686	0.011088
TAG49:2(18:2)	0.000438002	0.0002459	0.0002964	0.0005681	0.0002377	0.0001808	0.0002333	0.0002746	0.0002154	0.0002442	0.087878
TAG49:2(18:1)	0.000743276	0.0002831	0.0004469	0.0007792	0.0002721	0.0002358	0.0002671	0.0001821	0.0003508	0.0002683	0.062146
TAG49:1(17:0)	0.000805157	0.0002645	0.0002917	0.0006837	0.0002481	0.000283	0.0002595	0.0002	0.0003631	0.0002476	0.160329
TAG49:1(18:1)	0.001030794	0.0004733	0.000574	0.001483	0.0004065	0.0004127	0.0003235	0.0002925	0.0006125	0.000509	0.125415
TAG49:0(17:0)	0.001504219	0.0004965	0.000541	0.0013221	0.0003617	0.0002751	0.0003724	0.0002656	0.0009048	0.0005434	0.195516
TAG50:3(18:2)	0.001256431	0.0013594	0.0009033	0.0017142	0.0008647	0.0010298	0.0007975	0.0010237	0.0009726	0.0011488	0.21415
TAG50:3(18:1)	0.00104855	0.0012342	0.0008281	0.0018399	0.0008267	0.0007272	0.0008125	0.0007282	0.0009633	0.0011041	0.189359
TAG50:2(16:0)	0.020585555	0.0355643	0.0151253	0.0368984	0.0248783	0.0326052	0.027631	0.0377257	0.0301459	0.0412084	0.177148
TAG50:2(18:2)	0.009905626	0.018615	0.0078849	0.0203695	0.012849	0.0165797	0.0141484	0.0196165	0.0148682	0.01955	0.291103
TAG50:2(18:1)	0.002163375	0.0012481	0.0009927	0.0031721	0.0007957	0.0009945	0.0009969	0.0010535	0.0011972	0.0015546	0.289492
TAG50:1(18:1)	0.004716144	0.0072706	0.0036555	0.0094508	0.0050948	0.0069495	0.0058272	0.0081772	0.0067063	0.010456	0.260729
TAG50:0(16:0)	0.006459273	0.0021668	0.0016231	0.0036898	0.0015915	0.0015683	0.0016138	0.0015131	0.0061277	0.003271	0.828193
TAG50:0(18:0)	0.004707266	0.0012063	0.0009174	0.0021214	0.000968	0.0009512	0.0008426	0.0006625	0.0057614	0.0016406	0.991999
TAG52:7(18:3)	0.00036276	0.0005522	0.0003058	0.0003619	0.0002549	0.0002083	0.0002219	0.000194	0.0002031	0.0002614	0.019974
TAG52:6(16:0)	0.002844682	0.0036748	0.0018677	0.0033027	0.0017293	0.0016862	0.0014333	0.001713	0.0018343	0.0016406	0.030823
TAG52:6(18:3)	0.006260182	0.008992	0.0043941	0.0088325	0.0039615	0.003856	0.0038371	0.0037932	0.0039333	0.0036527	0.036173
TAG52:6(18:2)	0.000721125	0.0010393	0.0005222	0.0008144	0.0005064	0.0004953	0.0004928	0.0005193	0.0006001	0.0004815	0.079955
TAG52:5(16:0)	0.010617961	0.0155016	0.008473	0.0178309	0.0082778	0.0097049	0.0086034	0.0110632	0.0111566	0.0124165	0.468138
TAG52:5(18:3)	0.013807735	0.0205962	0.0105854	0.0222949	0.0112472	0.0127708	0.0111051	0.0138804	0.0133756	0.0158767	0.390802
TAG52:5(18:2)	0.01411300	0.020331	0.011201	0.023792	0.011908	0.013073	0.011439	0.014990	0.015643	0.016647	0.490646

	9	7	7	9	6	5	8	6	9	1	
TAG52:5(18:1)	0.000464547	0.0005753	0.0003058	0.0005882	0.000403	0.0004166	0.0003386	0.000391	0.0004771	0.000509	0.522649
TAG52:4(16:0)	0.062526399	0.1044472	0.047366	0.1331055	0.0762126	0.0902294	0.0806659	0.1159555	0.0967911	0.1395419	0.314502
TAG52:4(18:3)	0.006180545	0.0100174	0.0050621	0.0118989	0.0065072	0.0073858	0.0061845	0.009189	0.0079804	0.0105558	0.832926
TAG52:4(18:2)	0.142409552	0.2394899	0.1132729	0.2948241	0.1570496	0.2064935	0.1781285	0.2665624	0.2291183	0.3125124	0.265377
TAG52:4(18:1)	0.005561119	0.008064	0.0040648	0.009667	0.005553	0.0069652	0.0061432	0.0087263	0.0070171	0.0099504	0.361695
TAG52:3(18:3)	0.001561705	0.0021204	0.0018301	0.0018047	0.0014641	0.0012067	0.0012414	0.0013072	0.001268	0.0013139	0.003026
TAG52:3(18:2)	0.043215031	0.0739218	0.0336239	0.0937389	0.0566909	0.0774153	0.0658103	0.0975087	0.0779329	0.1190163	0.09226
TAG52:3(18:1)	0.035795722	0.0661918	0.0284958	0.0820418	0.0519199	0.0718061	0.0579441	0.0864599	0.0705988	0.1075617	0.079622
TAG52:2(18:1)	0.02353642	0.0385245	0.0167343	0.0514868	0.031382	0.0435758	0.0343422	0.0538325	0.0426381	0.0708189	0.090787
TAG52:1(18:1)	0.003225198	0.0037768	0.0026064	0.0064647	0.0037755	0.0048937	0.0043337	0.0057539	0.0053367	0.0082961	0.102616
TAG52:0(16:0)	0.00212804	0.0009465	0.0009691	0.0013674	0.0006786	0.0007311	0.0007373	0.0005103	0.0028499	0.0012176	0.986464
TAG52:0(18:0)	0.005074509	0.0019534	0.0012702	0.0022974	0.0014055	0.0009945	0.0009631	0.000952	0.0073279	0.0022666	0.945035
TAG54:9(18:3)	0.001480398	0.0020484	0.0012309	0.0018897	0.0010317	0.0007801	0.0008335	0.0009003	0.0013179	0.0010154	0.029656
TAG54:8(18:3)	0.001290888	0.0018202	0.0011933	0.0018239	0.0010285	0.0009013	0.0009318	0.0009529	0.0015168	0.0013012	0.169371
TAG54:8(18:2)	0.000839701	0.0011847	0.0007835	0.0011736	0.0006008	0.0005688	0.0005276	0.0007076	0.0011447	0.0008889	0.382201
TAG54:7(18:3)	0.004662526	0.0066905	0.0042991	0.0082029	0.0042922	0.0041309	0.0040657	0.0049184	0.00569	0.0061057	0.484154
TAG54:7(18:2)	0.009714708	0.0133616	0.0080741	0.015278	0.0086557	0.0089527	0.0088093	0.010503	0.0135779	0.0129351	0.972485
TAG54:7(18:1)	0.000923733	0.0011486	0.0006384	0.0011155	0.0006804	0.0005858	0.0006324	0.0006731	0.0008209	0.0006872	0.086294
TAG54:6(18:3)	0.003125344	0.0045286	0.0026681	0.006252	0.0036056	0.0037934	0.003549	0.0046834	0.0055316	0.006336	0.392752
TAG54:6(18:2)	0.093502432	0.13712	0.0734183	0.1929721	0.1097599	0.1173275	0.114455	0.1565442	0.1806636	0.2073681	0.251397
TAG54:6(18:1)	0.003725784	0.0053511	0.0030557	0.0074225	0.004339	0.0047517	0.0041955	0.0058919	0.006823	0.0076916	0.305181
TAG54:5(18:3)	0.001363141	0.0021502	0.0011514	0.0030128	0.0019052	0.0018726	0.0018962	0.0028276	0.0027944	0.0031749	0.194791
TAG54:5(18:2)	0.04389625	0.0730965	0.0383518	0.1189984	0.0678372	0.0706462	0.0694529	0.1106499	0.1078923	0.1373706	0.149576
TAG54:5(18:1)	0.023646733	0.0412543	0.020464	0.0590833	0.0383436	0.0392613	0.0386324	0.0625505	0.0593876	0.077235	0.099135
TAG54:4(18:3)	0.000228538	0.0003712	0.0002328	0.0004422	0.0002569	0.0002594	0.0002864	0.0003675	0.0003819	0.0004301	0.48655
TAG54:4(18:2)	0.012976297	0.0213528	0.0110043	0.0338077	0.0200784	0.0208936	0.0202292	0.031745	0.0321501	0.043981	0.131841
TAG54:4(18:1)	0.015391064	0.0272672	0.0135804	0.0441297	0.0256995	0.0281689	0.025989	0.0421398	0.0408019	0.0583147	0.11956
TAG54:3(18:2)	0.001602315	0.0021902	0.0012267	0.003686	0.0021165	0.0021631	0.0020778	0.0032812	0.0032678	0.0045068	0.181185
TAG54:3(18:1)	0.008239144	0.0131102	0.0070872	0.0248259	0.0147756	0.0163561	0.0151841	0.0257394	0.0234128	0.033804	0.079563
TAG54:2(18:1)	0.001540785	0.0021206	0.0013075	0.0037534	0.0023021	0.0023944	0.0024074	0.0036901	0.0037826	0.0051634	0.091603
TAG54:1(18:1)	0.0003908	0.0004512	0.0004336	0.0006274	0.0004596	0.0003775	0.0004119	0.0004855	0.0005433	0.0006457	0.754735
TAG54:0(18:0)	0.001215119	0.0007464	0.0006036	0.0009395	0.0005742	0.0005307	0.0004929	0.0004347	0.0014902	0.000707	0.720664
TAG56:7(18:3)	8.05157E-05	0.0001315	0.0001171	9.641E-05	8.492E-05	5.307E-05	8.002E-05	7.061E-05	9.155E-05	6.034E-05	0.031266
TAG56:6(18:3)	0.000144506	0.0001379	0.0001254	0.0001759	9.977E-05	9.911E-05	9.622E-05	8.521E-05	0.0001525	0.0001068	0.130354
TAG56:5(18:3)	8.41195E-05	0.000156	0.0001101	0.0001744	0.000104	7.514E-05	8.753E-05	8.435E-05	0.000126	0.0001276	0.241021
LPS16:0	0.000416818	0.0005939	0.0004069	0.0005883	0.0003107	0.0001941	0.0001738	0.0002799	0.0002927	0.000233	0.00517
LPS18:3	0.00018661	0.0003875	0.0001763	0.0003088	0.0002709	0.0001663	0.0001537	0.0001463	0.0002156	6.467E-05	0.035764
LPS18:2	0.001064284	0.0021857	0.0012682	0.0027207	0.001442	0.0010441	0.0011323	0.0015244	0.0018334	0.0013269	0.317527
LPS18:1	0.004531028	0.0042229	0.0045643	0.0064124	0.004605	0.0041257	0.0034009	0.0028163	0.0033895	0.0035599	0.013217
LPS18:0	0.001514152	0.0013199	0.0010377	0.0018827	0.0009561	0.0007161	0.0006915	0.0007493	0.0009475	0.0008027	0.012191
LPS19:2	6.58365E-05	4.133E-05	1.363E-05	2.939E-05	3.983E-05	2.309E-05	4.032E-06	1.722E-05	1.546E-05	6.49E-06	0.027941

LPS19:1	1.09874E-05	8.248E-06	6.106E-05	0	0.0001115	1.853E-05	4.846E-05	0	7.758E-06	7.765E-05	0.766249
PS 34:2	0.010549399	0.0203281	0.0118218	0.0180951	0.0113023	0.0098931	0.0085621	0.0099804	0.010544	0.0083531	0.041621
PS 34:1	0.001386258	0.0031284	0.0014115	0.0024618	0.0017223	0.0013384	0.001394	0.0014469	0.0016667	0.0019486	0.229623
PS 36:2	0.005985057	0.0078785	0.006425	0.0082967	0.0064898	0.0055057	0.005054	0.0047902	0.0057628	0.0058909	0.011783
PS 36:1	0.014721096	0.0152125	0.0163817	0.0165612	0.0132432	0.0116253	0.0117746	0.009536	0.0100487	0.011072	0.00036
PS 38:5	0.000349839	0.0001698	0.0002556	0.0003362	0.0001995	0.0001418	0.0001893	9.394E-05	0.0002385	9.78E-05	0.041268
PS 38:4	0.00118409	0.001444	0.0010781	0.0015192	0.0007526	0.0004989	0.0006879	0.0005732	0.0006004	0.0004076	0.00222
PS 38:3	0.003415763	0.0044201	0.0027518	0.0050196	0.0028007	0.002514	0.0023725	0.0022615	0.0023524	0.0024627	0.021419
PS 38:2	0.013064284	0.0226363	0.0153464	0.0257053	0.014312	0.0144907	0.0123699	0.013432	0.0126965	0.0122859	0.076786
PS 38:1	0.001600381	0.0034759	0.0020635	0.0030534	0.0022392	0.0021991	0.0016662	0.0015656	0.0020572	0.0019577	0.13678
PS 40:4	0.000270905	0.0004099	0.0002979	0.0004803	0.000236	0.000142	0.0001477	0.0002272	0.0002669	0.0002127	0.026905
PS 40:3	0.002536507	0.0031817	0.0026809	0.0035011	0.0020761	0.0019788	0.0016019	0.0017535	0.0017146	0.0014704	0.003239
PS 40:2	0.014338119	0.0221013	0.013658	0.0232744	0.011547	0.0126958	0.0105053	0.0111753	0.0122392	0.0113115	0.055667
PS 40:1	0.001837709	0.0032796	0.0020707	0.0035487	0.0021211	0.0019473	0.0016757	0.0019457	0.0024005	0.0017982	0.134087
LPA16:2	0.000408819	0.0003223	0.0002755	0.0002891	0.0001602	0.0002476	0.000236	0.0002098	0.0001201	0.0002917	0.192012
LPA16:1	0.000634193	0.0006532	0.0005679	0.0005178	0.0005081	0.0003463	0.0003332	0.0003278	0.0003323	0.0006533	0.035318
LPA16:0	0.012337181	0.0241068	0.0188735	0.0203853	0.0111778	0.0113745	0.0102104	0.013301	0.0130063	0.0130305	0.073892
LPA18:3	0.007695488	0.0121408	0.010022	0.0099577	0.0054049	0.0044155	0.0041129	0.0048327	0.0044721	0.004318	0.003995
LPA18:2	0.034380369	0.071529	0.0591588	0.0545695	0.0332411	0.0332622	0.0310985	0.035988	0.0379386	0.0350524	0.066105
LPA18:1	0.00533056	0.0085693	0.0078971	0.0081999	0.0051542	0.0057565	0.0052835	0.0069737	0.0071779	0.0074061	0.5644
LPA18:0	0.002934163	0.002835	0.0028128	0.0037326	0.0022608	0.0019106	0.0020681	0.0021761	0.002706	0.0027964	0.083284
LPA19:3	0.002063434	0.0028631	0.0019698	0.0025408	0.0015302	0.0015095	0.0012906	0.0014813	0.0015471	0.0012049	0.011554
LPA19:2	0.001569001	0.0015741	0.0017379	0.0013243	0.0006886	0.0010891	0.0005783	0.0006598	0.0006781	0.0008876	0.01982
LPA19:1	0.000978054	0.000866	0.0010067	0.0013004	0.0006958	0.0007078	0.0006617	0.0005462	0.0008409	0.0008624	0.065493
PA32:2	0.002572898	0.0071025	0.0098405	0.0031871	0.0021764	0.0040362	0.0045661	0.0045564	0.0022432	0.0041908	0.517232
PA32:1	0.000813068	0.0020636	0.0024913	0.0007614	0.0004843	0.000704	0.0008007	0.0007929	0.0004019	0.0007236	0.154811
PA32:0	0.000779578	0.001822	0.001653	0.0010623	0.0006011	0.0008931	0.0010297	0.0011679	0.0008362	0.0007339	0.345856
PA34:3	0.019436449	0.0700973	0.0807039	0.0227427	0.0167514	0.0257282	0.0274255	0.0263995	0.0163848	0.0199095	0.215439
PA34:2	0.106049077	0.4308834	0.4656596	0.1472673	0.1126637	0.198998	0.228309	0.2276269	0.1321473	0.1855894	0.50133
PA34:1	0.014918781	0.0649465	0.0686769	0.0205898	0.0178427	0.0335256	0.0349757	0.0332553	0.0222216	0.031548	0.622136
PA36:6	0.00585857	0.0214751	0.0249178	0.0062708	0.0040027	0.0056933	0.0068164	0.0047365	0.0027737	0.0038191	0.121783
PA36:5	0.014189481	0.0574355	0.0623969	0.0165095	0.012574	0.0228854	0.0230214	0.0217786	0.0118022	0.0185328	0.285986
PA36:4	0.079790331	0.3409286	0.3855412	0.103484	0.0798315	0.1693797	0.1750783	0.1647841	0.1046601	0.1602824	0.550859
PA36:3	0.023677058	0.0933016	0.1091305	0.0321629	0.0245118	0.049945	0.0497417	0.0479401	0.0330508	0.0454375	0.561851
PA36:2	0.011541078	0.0355878	0.0414704	0.0115921	0.0108408	0.0173384	0.019361	0.0188301	0.0124371	0.0172363	0.471738
PA36:1	0.002162496	0.0060246	0.0068903	0.0022	0.0019663	0.0031515	0.0038321	0.0032369	0.0024683	0.0031357	0.550087
PA38:6	0.000418928	0.0016544	0.0025168	0.0006583	0.0003734	0.0005413	0.0005814	0.0006849	0.000394	0.000764	0.244591
PA38:5	0.000595166	0.000818	0.0015763	0.0004233	0.0004085	0.000661	0.0004387	0.0005768	0.0003537	0.0006434	0.335938
PA38:4	0.00099748	0.0036252	0.0046027	0.0012694	0.0007411	0.0016574	0.0017636	0.0012905	0.0007799	0.0015076	0.321157
PA38:3	0.001207032	0.0041099	0.0061027	0.0014293	0.0009102	0.0019493	0.0027551	0.0020835	0.0014148	0.0021412	0.528701
PA38:2	0.002581424	0.009018	0.0119251	0.0031873	0.0020655	0.0035211	0.0043662	0.0039001	0.0023798	0.0028642	0.278121
PA38:1	0.00834778	0.033505	0.036334	0.011131	0.008448	0.017724	0.017540	0.017186	0.009430	0.014542	0.529929

	8	2	1	6	6	6	2	3	5	7	
PA40:6	0.000234691	0.0013574	0.0011182	0.0003384	0.0004025	0.0005238	0.0005625	0.0004758	0.0003457	0.0006633	0.473748
PA40:5	0.000653706	0.0022686	0.0023902	0.0008179	0.0006768	0.0008588	0.0010007	0.000656	0.0004662	0.0005628	0.148806
PA40:4	0.0012908	0.0044066	0.0052126	0.0014574	0.0010152	0.002104	0.0019068	0.001932	0.0008685	0.0015478	0.301675
PA40:3	0.005422678	0.0225168	0.0279434	0.0072298	0.0052804	0.0127094	0.0137559	0.0117147	0.0067534	0.0106332	0.618653
PA40:2	0.00317659	0.0142981	0.0163752	0.0049077	0.0033433	0.0080808	0.0079121	0.0078723	0.0043817	0.0073169	0.668512
PA40:1	0.00159247	0.0048158	0.0063315	0.0017675	0.0010793	0.0026364	0.002898	0.0022708	0.0017205	0.0021006	0.475266
CL72:11(18:3)	0.00031591	0.0025078	0.0024698	0.0005249	0.0002625	0.000721	0.0021965	0.000514	0.0002962	0.0005838	0.585481
CL72:11(18:2)	0.000129212	0.0022114	0.001164	0.0002374	0.0001859	0.000459	0.0005999	0.0003034	0.0001032	0.0002272	0.31109
CL72:11(18:1)	5.74861E-05	0.0005904	0.0013079	0.0001018	6.573E-05	0.00023	0.0007496	0.0001868	0.0001416	6.518E-05	0.595418
CL72:10(18:3)	0.000689218	0.0081833	0.0065397	0.0010323	0.0006775	0.0012119	0.0022969	0.0011425	0.0005282	0.00146	0.24073
CL72:10(18:2)	0.000933226	0.0087727	0.0074116	0.0011506	0.0008087	0.0026877	0.0038441	0.0019592	0.0010047	0.0013943	0.397232
CL72:10(18:1)	0.000143452	0.0005904	0.0021821	0.0001863	0.0001859	0.0006886	0.0007489	0.0003964	0.0001418	0.0002267	0.609109
CL72:9(18:3)	0.00117741	0.0140811	0.0114792	0.0017936	0.0014864	0.0047833	0.0051435	0.0038499	0.001507	0.003082	0.440792
CL72:9(18:2)	0.005829651	0.0580106	0.0475193	0.0071078	0.0053336	0.0184452	0.0202203	0.0150467	0.0069167	0.0134915	0.423639
CL72:9(18:1)	0.000172546	0.0011068	0.0018878	1.698E-05	0.000142	0.0003932	0.0004509	0.0002803	0.0001546	0.0001304	0.327814
CL72:9(18:0)	4.30706E-05	0.0002953	0	0.0001186	3.298E-05	0.0001642	0.0004493	0.0002796	0.0001288	0.0002919	0.065855
CL72:8(18:3)	0.000516847	0.004572	0.005816	0.0005925	0.0004918	0.0013112	0.0024968	0.0014928	0.0007475	0.0011997	0.450033
CL72:8(18:2)	0.02168034	0.2030799	0.1755526	0.0309844	0.0227203	0.0818433	0.0969086	0.0757704	0.0332449	0.0779703	0.684275
CL72:8(18:1)	0.000416554	0.003316	0.0066879	0.0007783	0.0004919	0.0014419	0.0019973	0.0017734	0.0009143	0.000973	0.477222
FFA18:2	0.23357448	0.1955641	0.2133572	0.2285362	0.2096713	0.1380724	0.1492153	0.1233667	0.1346863	0.125538	8.97E-06
FFA18:1	0.234055552	0.1179082	0.2004515	0.1745785	0.1532022	0.0923103	0.1127018	0.0737711	0.1683689	0.0906963	0.028788
FFA18:0	2.567416613	2.0449156	2.4064794	3.0895415	2.7419081	1.8167948	1.9673522	1.3847302	2.0220083	2.0452407	0.009283
FFA16:1	0.258480545	0.2909288	0.1983389	0.3726989	0.307968	0.1400104	0.1894549	0.1289921	0.1295624	0.1737733	0.002732
FFA16:0	3.548173835	2.780051	2.9837899	4.1720283	3.3361959	2.2243331	2.4624095	1.8180133	2.3984786	2.6645714	0.005719
S1P t18:1	0.00015	0.0003001	0.0002635	0.0002916	0.0001451	0.0001061	8.505E-05	0.0001093	0.0001116	0.0001024	0.006223
S1P t18:0	3.61711E-05	4.366E-05	3.944E-05	5.112E-05	1.92E-05	1.56E-05	1.345E-05	1.719E-05	1.739E-05	1.53E-05	0.003275
Sph d18:1	0.000104017	0.0001359	0.0001404	0.0001112	7.241E-05	6.309E-05	5.886E-05	5.477E-05	5.887E-05	5.078E-05	0.002098
Sph d18:0	0.0001745	0.0002783	0.0001999	0.000231	0.0001777	0.0001004	0.0001093	0.00011	9.856E-05	8.171E-05	0.000506
Sph d20:0	8.09698E-06	9.096E-06	8.474E-06	1.001E-05	6.973E-06	6.796E-06	6.115E-06	7.751E-06	8.286E-06	8.485E-06	0.162374
Sph t18:0	0.003581657	0.0056912	0.0045601	0.0058213	0.0032824	0.0020359	0.0021942	0.0018786	0.0020963	0.0018616	0.001206
Cer d18:1/16:0	1.50217E-05	2.976E-05	2.317E-05	1.726E-05	1.398E-05	1.849E-05	1.219E-05	1.358E-05	1.484E-05	1.464E-05	0.142398
Cer d18:1/18:0	1.53583E-05	2.788E-05	1.632E-05	1.545E-05	1.52E-05	1.605E-05	1.58E-05	1.501E-05	1.942E-05	1.634E-05	0.572224
Cer d18:1/20:1	3.13762E-05	5.724E-05	2.939E-05	4.264E-05	3.384E-05	5.986E-05	7.031E-05	5.897E-05	7.781E-05	5.623E-05	0.004306
Cer d18:1/20:0	3.1516E-05	4.379E-05	3.376E-05	2.776E-05	2.276E-05	2.642E-05	2.146E-05	2.64E-05	3.226E-05	3.129E-05	0.309345
Cer d18:1/22:1	6.12921E-06	1.017E-05	8.041E-06	8.727E-06	7.447E-06	8.995E-06	9.387E-06	9.714E-06	1.008E-05	6.902E-06	0.325513
Cer d18:1/22:0	8.72224E-06	1.407E-05	1.224E-05	1.028E-05	6.194E-06	6.704E-06	7.488E-06	6.919E-06	6.96E-06	6.502E-06	0.039245
Cer d18:1/24:1	7.39906E-06	1.438E-05	1.305E-05	1.162E-05	1.009E-05	1.382E-05	1.294E-05	1.476E-05	1.737E-05	1.265E-05	0.076905
Cer d18:1/24:0	1.87636E-05	3.071E-05	2.86E-05	2.619E-05	1.898E-05	2.227E-05	2.104E-05	2.263E-05	2.727E-05	1.981E-05	0.481691
Cer d18:1/26:1	2.40287E-06	4.028E-06	4.241E-06	3.335E-06	3.495E-06	2.75E-06	5.304E-06	4.844E-06	3.861E-06	3.224E-06	0.41479
Cer d18:1/26:0	4.18488E-06	8.479E-06	6.545E-06	7.867E-06	4.622E-06	4.812E-06	4.68E-06	4.956E-06	7.996E-06	4.312E-06	0.388734
Cer d18:0/16:0	1.39027E-06	1.358E-06	1.815E-06	1.162E-06	1.38E-06	1.217E-06	1.126E-06	1.614E-06	1.801E-06	1.062E-06	0.759988

Cer d18:0/18:0	6.47817E-07	1.629E-06	4.576E-07	1.415E-06	1.038E-06	8.878E-07	1.085E-06	1.068E-06	8.186E-07	9.063E-07	0.719967
Cer d18:0/20:1	5.53472E-07	9.655E-07	1.251E-06	1.095E-06	6.202E-07	7.448E-07	3.119E-07	4.844E-07	2.047E-07	6.085E-07	0.033622
Cer d18:0/20:0	7.96367E-07	1.313E-06	1.297E-06	7.244E-07	6.71E-07	1.146E-06	1.451E-06	1.789E-06	1.706E-06	1.398E-06	0.018611
Cer d18:0/22:1	3.29358E-06	2.127E-06	2.364E-06	2.678E-06	2.013E-06	3.709E-06	4.232E-06	3.266E-06	3.698E-06	2.862E-06	0.011728
Cer d18:0/22:0	1.59273E-06	1.177E-06	1.464E-06	1.533E-06	1.633E-06	2.807E-06	1.275E-06	2.111E-06	1.828E-06	2.266E-06	0.061002
Cer d18:0/24:0	1.16086E-06	8.146E-07	6.863E-07	1.011E-06	7.723E-07	1.232E-06	6.375E-07	9.686E-07	8.868E-07	4.142E-07	0.720095
Cer d18:0/26:0	1.61998E-06	1.524E-06	1.129E-06	1.347E-06	1.127E-06	4.296E-07	1.275E-06	8.817E-07	1.405E-06	3.495E-07	0.076244
PhytoCer t18:1/16:0	2.89648E-05	3.736E-05	3.292E-05	4.26E-05	2.948E-05	3.364E-05	3.357E-05	2.391E-05	2.104E-05	2.37E-05	0.092159
PhytoCer t18:1/18:0	2.16387E-05	1.893E-05	2.204E-05	2.865E-05	1.732E-05	2.945E-05	2.262E-05	1.881E-05	2.239E-05	1.989E-05	0.741253
PhytoCer t18:1/20:0	1.81989E-05	2.675E-05	2.041E-05	2.139E-05	1.919E-05	1.507E-05	1.194E-05	1.58E-05	1.676E-05	1.551E-05	0.006719
PhytoCer t18:1/20:1	4.10401E-06	5.28E-06	3.219E-06	3.74E-06	3.242E-06	2.406E-06	3.038E-06	1.987E-06	2.783E-06	2.668E-06	0.012619
PhytoCer t18:1/22:0	2.0237E-05	2.763E-05	2.125E-05	2.352E-05	1.966E-05	1.532E-05	1.491E-05	1.789E-05	1.596E-05	1.59E-05	0.003001
PhytoCer t18:1/22:1	1.81641E-05	2.253E-05	2.014E-05	1.899E-05	1.773E-05	1.762E-05	1.873E-05	1.338E-05	2.172E-05	1.591E-05	0.24775
PhytoCer t18:1/24:0	0.000104229	0.0001463	0.0001211	0.0001433	9.116E-05	8.768E-05	6.196E-05	9.919E-05	8.29E-05	6.26E-05	0.011461
PhytoCer t18:1/24:1	2.02016E-05	2.837E-05	2.335E-05	2.794E-05	2.033E-05	1.99E-05	1.731E-05	1.478E-05	1.885E-05	1.611E-05	0.010413
PhytoCer t18:1/26:0	2.29408E-05	3.596E-05	3.051E-05	3.773E-05	2.425E-05	2.468E-05	1.548E-05	2.191E-05	2.298E-05	2.099E-05	0.027348
PhytoCer t18:1/26:1	3.49634E-06	5.205E-06	5.217E-06	6.536E-06	4.622E-06	4.812E-06	2.699E-06	4.185E-06	2.702E-06	3.781E-06	0.064742
PhytoCer t18:1/28:0	1.06622E-06	1.87E-06	2.319E-06	1.499E-06	1.583E-06	1.074E-06	1.451E-06	6.582E-07	9.55E-07	1.308E-06	0.04913
PhytoCer t18:1/28:1	1.5523E-06	1.916E-06	2.074E-06	1.971E-06	2.292E-06	2.492E-06	2.061E-06	1.366E-06	2.592E-06	1.269E-06	0.987457
PhytoCer t18:0/16:0	1.32488E-05	1.86E-05	1.25E-05	1.771E-05	1.023E-05	1.389E-05	9.578E-06	1.006E-05	7.369E-06	9.754E-06	0.053349
PhytoCer t18:0/18:0	3.83053E-05	4.097E-05	2.65E-05	4.13E-05	3.581E-05	3.368E-05	3.166E-05	3.238E-05	3.219E-05	3.02E-05	0.138288
PhytoCer t18:0/20:0	0.000106353	0.0001326	0.000105	0.0001316	0.0001009	8.796E-05	8.245E-05	8.402E-05	8.303E-05	5.716E-05	0.003409
PhytoCer t18:0/20:1	5.31907E-06	5.809E-06	5.843E-06	7.211E-06	4.318E-06	5.213E-06	4.544E-06	4.111E-06	2.729E-06	3.665E-06	0.030291
PhytoCer t18:0/22:0	0.0001956	0.0002888	0.000191	0.0002469	0.0001892	0.0001664	0.0001408	0.0001743	0.0001847	0.0001336	0.022404
PhytoCer t18:0/22:1	1.168E-05	1.479E-05	1.009E-05	1.144E-05	1.12E-05	9.727E-06	5.928E-06	7.142E-06	6.237E-06	7.162E-06	0.002121
PhytoCer t18:0/24:0	0.001038393	0.0012681	0.0009696	0.0011685	0.0009132	0.0008542	0.0006553	0.0009683	0.0007649	0.0008726	0.018061
PhytoCer t18:0/24:1	1.63413E-05	2.334E-05	1.967E-05	2.26E-05	1.849E-05	1.337E-05	1.373E-05	1.637E-05	1.238E-05	1.426E-05	0.003138
PhytoCer t18:0/26:0	0.000160965	0.0001679	0.0001421	0.0001893	0.0001325	0.0001405	9.648E-05	0.0001423	0.0001291	0.0001274	0.040932
PhytoCer t18:0/26:1	8.19602E-06	8.571E-06	7.644E-06	9.519E-06	6.979E-06	7.334E-06	5.549E-06	5.701E-06	6.018E-06	5.297E-06	0.004288
PhytoCer t18:0/28:0	2.70261E-05	4.9E-05	4.568E-05	4.109E-05	4.375E-05	4.546E-05	4.756E-05	3.909E-05	4.208E-05	4.661E-05	0.507686
PhytoCer t18:0/28:1	1.63317E-06	2.776E-06	2.014E-06	2.476E-06	1.089E-06	1.604E-06	1.343E-06	2.521E-06	1.91E-06	1.334E-06	0.512421
PhytoCer t18:1/h16:0	1.87636E-06	1.841E-06	2.136E-06	1.92E-06	8.104E-07	2.649E-06	1.438E-06	8.692E-07	2.101E-06	8.676E-07	0.7619
PhytoCer t18:0/h16:0	1.0126E-06	5.733E-07	9.154E-07	6.736E-07	4.684E-07	7.734E-07	5.017E-07	5.342E-07	4.364E-07	8.157E-07	0.389492
PhytoCer t18:1/h18:0	5.83211E-06	5.13E-06	4.79E-06	7.008E-06	4.192E-06	5.672E-06	5.521E-06	5.975E-06	5.09E-06	6.696E-06	0.489765
PhytoCer t18:1/h20:0	1.32326E-05	1.776E-05	1.016E-05	1.864E-05	1.649E-05	1.928E-05	1.232E-05	1.516E-05	1.216E-05	1.376E-05	0.732737
PhytoCer t18:1/h21:0	0.000104322	0.0001457	0.0001125	0.0001384	9.376E-05	0.000158	0.000132	0.0001186	0.0001585	0.0001395	0.113289
PhytoCer t18:1/h22:0	3.89311E-05	5.502E-05	3.836E-05	5.253E-05	4.28E-05	3.35E-05	3.116E-05	2.364E-05	2.766E-05	2.438E-05	0.002266
PhytoCer t18:0/h22:1	5.83264E-05	8.239E-05	5.885E-05	7.462E-05	5.458E-05	5.474E-05	3.631E-05	5.674E-05	5.074E-05	4.029E-05	0.028379
PhytoCer t18:0/h22:0	3.37911E-05	5.224E-05	4.188E-05	4.756E-05	4.008E-05	3.962E-05	2.843E-05	4.179E-05	3.519E-05	2.917E-05	0.081698
PhytoCer t18:1/h23:0	1.63932E-05	2.288E-05	2.375E-05	2.19E-05	1.671E-05	1.58E-05	1.149E-05	1.552E-05	1.519E-05	1.475E-05	0.010995
PhytoCer t18:0/h23:0	3.77978E-05	4.595E-05	3.907E-05	4.969E-05	3.633E-05	4.193E-05	2.755E-05	4.382E-05	3.584E-05	3.071E-05	0.190498
PhytoCer	1.88277E-	2.486E-	1.981E-	2.238E-	1.835E-	1.933E-	1.323E-	2.105E-	1.896E-	1.254E-	0.108698

t18:1/h24:1	05	05	05	05	05	05	05	05	05	05	
PhytoCer t18:1/h24:0	0.000180754	0.0001897	0.0001947	0.0002358	0.0002024	0.0001586	0.0001526	0.0001566	0.0001636	0.0001374	0.002032
PhytoCer t18:0/h24:1	8.8443E-05	8.834E-05	8.247E-05	8.866E-05	7.007E-05	7.938E-05	6.472E-05	7.326E-05	7.441E-05	5.886E-05	0.030282
PhytoCer t18:0/h24:0	0.000145544	0.0002339	0.0001805	0.0002024	0.0001777	0.0001837	0.0001564	0.0001454	0.0001651	0.0001541	0.128789
PhytoCer t18:1/h25:0	1.91887E-05	2.966E-05	2.158E-05	2.691E-05	2.302E-05	2.053E-05	1.856E-05	1.891E-05	1.787E-05	1.889E-05	0.028967
GluCer d18:1/h16:0	9.18312E-05	0.0001309	8.177E-05	0.0001265	9.079E-05	0.00012	0.0001051	0.0001127	0.0001128	0.0001008	0.593787
GluCer d18:1/h18:0	7.72877E-05	0.0001171	7.581E-05	0.0001145	7.561E-05	9.046E-05	8.028E-05	8.808E-05	0.0001028	0.0001025	0.944056
GluCer d18:1/h19:0	1.04875E-05	1.191E-05	9.204E-06	1.323E-05	9.832E-06	7.675E-06	8.118E-06	6.552E-06	1.015E-05	1.099E-05	0.075516
GluCer d18:1/h20:0	0.000200323	0.0002934	0.000187	0.000257	0.0001744	0.0002209	0.0001865	0.0002131	0.0002614	0.0002527	0.868789
GluCer d18:1/h22:0	4.01981E-05	6.326E-05	3.793E-05	4.962E-05	3.455E-05	4.905E-05	4.751E-05	4.479E-05	6.072E-05	5.161E-05	0.364848
GluCer d18:1/h23:0	4.23117E-06	7.197E-06	5.217E-06	7.279E-06	4.548E-06	4.705E-06	4.265E-06	3.519E-06	4.695E-06	4.191E-06	0.072064
GluCer d18:1/h24:1	9.19279E-06	1.224E-05	8.613E-06	1.295E-05	7.465E-06	9.419E-06	7.667E-06	8.124E-06	1.068E-05	8.982E-06	0.373925
GluCer d18:1/h24:0	4.32282E-05	5.938E-05	3.725E-05	5.379E-05	3.548E-05	6.635E-05	5.843E-05	6.319E-05	8.403E-05	7.533E-05	0.006763
GluCer d18:1/h25:1	2.23557E-06	3.957E-06	2.648E-06	4.227E-06	1.327E-06	1.898E-06	1.105E-06	2.024E-06	2.391E-06	2.9E-06	0.222919
GluCer d18:1/h25:0	4.70144E-06	5.832E-06	3.583E-06	4.893E-06	4.345E-06	3.371E-06	3.37E-06	3.176E-06	5.1E-06	4.207E-06	0.147042
GluCer d18:1/h26:1	2.49429E-06	3.922E-06	2.953E-06	4.493E-06	1.919E-06	2.618E-06	1.588E-06	2.6E-06	2.836E-06	2.227E-06	0.168613
GluCer d18:1/h26:0	7.34076E-06	8.702E-06	6.379E-06	8.176E-06	6.154E-06	6.947E-06	6.406E-06	7.245E-06	1.011E-05	8.686E-06	0.544829
GluCer d18:0/h20:0	4.72048E-06	7.036E-06	3.986E-06	7.231E-06	3.906E-06	9.115E-06	7.332E-06	7.053E-06	1.116E-05	1.028E-05	0.010455
GluCer d18:0/h22:0	3.51157E-06	4.478E-06	2.667E-06	3.742E-06	3.229E-06	6.84E-06	6.188E-06	6.545E-06	7.818E-06	7.239E-06	3.4E-05
GluCer t18:1/h16:0	6.97334E-06	8.006E-06	6.801E-06	7.185E-06	5.056E-06	5.242E-06	4.7E-06	4.096E-06	4.313E-06	4.31E-06	0.002496
GluCer t18:1/h18:0	2.82244E-06	4.535E-06	3.62E-06	6.294E-06	2.994E-06	3.792E-06	3.058E-06	3.671E-06	3.69E-06	2.974E-06	0.37725
GluCer t18:1/h19:0	2.22824E-06	2.609E-06	3.229E-06	2.766E-06	1.088E-06	1.702E-06	1.423E-06	2.184E-06	1.983E-06	2.791E-06	0.417623
GluCer t18:1/h20:0	3.55544E-05	4.893E-05	3.104E-05	4.794E-05	4.06E-05	3.089E-05	2.76E-05	2.776E-05	2.827E-05	3.392E-05	0.016272
GluCer t18:1/h22:0	8.80809E-05	0.000145	8.877E-05	0.0001381	9.578E-05	7.791E-05	6.793E-05	6.914E-05	7.912E-05	6.928E-05	0.016687
GluCer t18:1/h23:0	2.43627E-05	3.459E-05	2.882E-05	3.572E-05	2.684E-05	1.924E-05	1.789E-05	1.454E-05	1.829E-05	1.906E-05	0.000826
GluCer t18:1/h24:1	7.7908E-06	9.284E-06	7.469E-06	1.067E-05	6.488E-06	3.896E-06	5.754E-06	3.91E-06	3.798E-06	5.137E-06	0.001771
GluCer t18:1/h24:0	0.000377535	0.000532	0.0003544	0.0005147	0.0003745	0.0002629	0.0002546	0.0002504	0.000267	0.000296	0.002926
GluCer t18:1/h25:1	2.67389E-06	5.218E-06	3.239E-06	4.056E-06	3.002E-06	2.913E-06	2.312E-06	1.467E-06	2.768E-06	2.783E-06	0.054403
GluCer t18:1/h25:0	2.77281E-05	3.382E-05	2.715E-05	3.897E-05	2.81E-05	1.898E-05	1.543E-05	1.646E-05	1.927E-05	2.039E-05	0.000753
GluCer t18:1/h26:1	3.88134E-06	3.628E-06	2.743E-06	5.298E-06	2.79E-06	2.977E-06	1.936E-06	1.846E-06	2.745E-06	3.22E-06	0.071891
GluCer t18:1/h26:0	3.77533E-05	5.238E-05	3.528E-05	4.951E-05	3.484E-05	2.085E-05	2.27E-05	2.036E-05	2.441E-05	2.619E-05	0.001197

Table S6 Enriched GO terms analysis of compartments-specific genes in smk10 mutant compared with wild-type. Compartments-specific genes were selected based on public data (Zhan et al., 2015).

A: Enriched GO terms analysis of compartments-specific genes in 6 DAP *smk10* mutant compared with wild-type.

BETL 6 DAP						
GO term	Ontology	Description	Number in input list	Number in BG/Ref	p-value	FDR

GO:0006952	P	defense response	17	3346	1.20E-05	0.013
GO:0001906	P	cell killing	3	47	6.90E-05	0.019
GO:0031640	P	killing of cells of other organism	3	44	5.70E-05	0.019
GO:0044364	P	disruption of cells of other organism	3	44	5.70E-05	0.019
GO:0005576	C	extracellular region	15	2695	1.50E-05	0.0024

B: Enriched GO terms analysis of compartments-specific genes in 12 DAP *smk10* mutant compared with wild-type.

AL 12 DAP						
GO term	Ontology	Description	DE AL Genes	Number in BG/Ref	p-value	FDR
GO:0048316	P	kernel development	14	1259	3.7E-06	0.0053
GO:0010154	P	fruit development	15	1383	2.2E-06	0.0053
GO:0009415	P	response to water	11	1025	0.00006	0.036
GO:0001101	P	esponse to acid chemical	20	3035	0.000063	0.036
GO:0048608	P	reproductive structure development	20	3139	0.0001	0.041
GO:0010431	P	kernel maturation	6	306	0.00014	0.045
GO:0005811	C	lipid particle	4	81	0.000065	0.024
BETL 12 DAP						
GO term	Ontology	Description	DE AL Genes	Number in BG/Ref	p-value	FDR
GO:0001906	P	cell killing	5	47	2.6E-06	0.0034
GO:0031640	P	killing of cells of other organism	5	44	1.9E-06	0.0034
GO:0044364	P	disruption of cells of other organism	5	44	1.9E-06	0.0034
CSE 12 DAP						
GO term	Ontology	Description	DE AL Genes	Number in BG/Ref	p-value	FDR
GO:0042255	P	ribosome assembly	11	221	6.4E-12	9.5E-09
GO:0070925	P	organelle assembly	11	372	1.3E-09	1.3E-09
GO:0006412	P	translation	24	2568	2.4E-09	1.4E-06
GO:0043043	P	peptide biosynthetic process	24	2596	3E-09	1.5E-06
GO:0022618	P	ribonucleoprotein complex assembly	11	414	3.9E-09	1.6E-06
GO:1901566	P	organonitrogen compound biosynthetic process	31	4751	3.2E-08	8.7E-06
GO:0042254	P	ribosome biogenesis	13	1078	1.2E-06	0.00027
GO:1901564	P	organonitrogen compound metabolic process	34	7304	0.000016	0.0031
GO:0019252	P	starch biosynthetic process	6	262	0.000036	0.0067
GO:0005977	P	glycogen metabolic process	4	84	0.000051	0.0089
GO:0005198	F	structural molecule activity	22	1252	8.9E-14	1.5E-11
GO:0045735	F	nutrient reservoir activity	5	106	5.9E-06	0.00064
GO:0035251	F	UDP-glucosyltransferase activity	7	402	0.000044	0.0036
GO:0046527	F	glucosyltransferase activity	7	423	0.00006	0.0039
GO:0022626	C	cytosolic ribosome	23	534	1.2E-22	5.1E-20
GO:0044391	C	ribosomal subunit	21	524	4.3E-20	6.2E-18
GO:0022625	C	cytosolic large ribosomal subunit	13	231	1.5E-14	1.7E-12
GO:0005840	C	ribosome	23	1443	1.6E-13	1.4E-11
GO:0015934	C	large ribosomal subunit	13	299	3.6E-13	2.6E-11
GO:0022627	C	cytosolic small ribosomal subunit	8	148	3E-09	1.3E-07

GO:0009501	C	amyloplast	6	48	2.8E-09	1.3E-07
GO:1990904	C	ribonucleoprotein complex	23	2553	1.1E-08	3.7E-07
GO:0009506	C	plasmodesma	15	1850	0.00002	0.00044
GO:0005911	C	cell-cell junction	15	1892	0.000026	0.00054
CZ 12 DAP						
GO term	Ontology	Description	DE AL Genes	Number in BG/Ref	p-value	FDR
GO:0006091		generation of precursor metabolites and energy	13	1278	3.8E-08	0.00011
GO:0009408		response to heat	8	589	2.6E-06	0.0018
GO:0009628		response to abiotic stimulus	23	5674	2.3E-06	0.0018
GO:0015979		photosynthesis	7	585	0.000026	0.014
GO:0009888		tissue development	13	2545	0.000071	0.025
GO:0045333		cellular respiration	5	303	0.000092	0.029
EMB 12 DAP						
GO term	Ontology	Description	DE AL Genes	Number in BG/Ref	p-value	FDR
GO:0009657	P	plastid organization	23	736	7.7E-12	4.9E-08
GO:0048367	P	shoot system development	37	2650	2.1E-08	0.000015
GO:0009416	P	response to light stimulus	34	2186	6.6E-09	0.000015
GO:0006334	P	nucleosome assembly	10	161	1.9E-08	0.000015
GO:0009240	P	isopentenyl diphosphate biosynthetic process	13	339	3.3E-08	0.000015
GO:0015995	P	chlorophyll biosynthetic process	10	184	6.1E-08	0.000024
GO:0034728	P	nucleosome organization	10	183	5.9E-08	0.000024
GO:0031497	P	chromatin assembly	10	193	9.4E-08	0.000035
GO:0019682	P	glyceraldehyde-3-phosphate metabolic process	15	529	1.3E-07	0.000042
GO:0048646	P	anatomical structure formation involved in morphogenesis	32	2336	3.4E-07	0.000089
GO:0006323	P	DNA packaging	10	232	4.8E-07	0.00012
GO:0009628	P	response to abiotic stimulus	56	5674	4.6E-07	0.00012
GO:0008610	P	lipid biosynthetic process	26	2086	0.000024	0.0024
GO:0046982	F	protein heterodimerization activity	14	274	2.9E-10	2E-07
GO:0044815	C	DNA packaging complex	10	176	4.1E-08	8.8E-06
GO:0000786	C	nucleosome	10	170	3E-08	8.8E-06
GO:0009536	C	plastid	58	5796	1.7E-07	0.000017
GO:0009941	C	chloroplast envelope	21	1132	4.4E-07	0.000031
GO:0034357	C	photosynthetic membrane	16	715	1.1E-06	0.000053
GO:0009535	C	chloroplast thylakoid membrane	15	622	9.7E-07	0.000053
GO:0031976	C	plastid thylakoid	16	778	3.1E-06	0.00012
GO:0005618	C	cell wall	24	1901	0.000042	0.0015
ESR 12 DAP						
GO term	Ontology	Description	DE AL Genes	Number in BG/Ref	p-value	FDR
GO:0045168	P	cell-cell signaling involved in cell fate commitment	3	21	0.000012	0.028
GO:0033612	F	receptor serine/threonine kinase binding	3	41	0.000073	0.02
GO:0005576	C	extracellular region	18	2695	1.3E-06	0.00034
GO:0048046	C	apoplast	10	905	6.3E-06	0.00084
GO:0044421	C	extracellular region part	7	527	0.000056	0.0049

NU 12 DAP						
GO term	Ontology	Description	DE AL Genes	Number in BG/Ref	p-value	FDR
GO:0005576	C	extracellular region	29	2695	5.3E-07	0.00025
GO:0009505	C	plant-type cell wall	13	780	0.000013	0.0032
GO:0005618	C	cell wall	20	1901	0.000052	0.0082
GO:0030312	C	external encapsulating structure	20	1960	0.000078	0.0093
GO:0048046	C	apoplast	12	905	0.00024	0.023
PC 12 DAP						
GO term	Ontology	Description	DE AL Genes	Number in BG/Ref	p-value	FDR
GO:0006950	P	response to stress	78	12007	3.8E-08	0.000088
GO:0009605	P	response to external stimulus	38	4265	5.1E-07	0.00078
GO:0044255	P	cellular lipid metabolic process	27	2844	0.000011	0.0061
GO:0009651	P	response to salt stress	20	1708	9.5E-06	0.0061
GO:0006631	P	fatty acid metabolic process	15	1090	0.000022	0.01
GO:0043207	P	response to external biotic stimulus	26	2998	0.000072	0.021
GO:0009607	P	response to biotic stimulus	27	3201	0.000082	0.022
GO:0006629	P	lipid metabolic process	31	4009	0.00011	0.027
GO:0072330	P	monocarboxylic acid biosynthetic process	14	1163	0.00017	0.039
GO:0004497	F	monooxygenase activity	10	522	0.000039	0.0084
GO:0015926	F	glucosidase activity	5	132	0.00018	0.024
GO:0016491	F	oxidoreductase activity	28	3759	0.00046	0.049
GO:0005576	C	extracellular region	28	2695	1.3E-06	0.0006
GO:0005783	C	endoplasmic reticulum	21	2031	0.000035	0.008
GO:0071944	C	cell periphery	50	8255	0.00031	0.044
GO:0005618	C	cell wall	18	1901	0.00038	0.044
PE 12 DAP						
GO term	Ontology	Description	DE AL Genes	Number in BG/Ref	p-value	FDR
GO:0009605	P	response to external stimulus	49	4265	1.2E-06	0.0025
GO:0090558	P	plant epidermis development	20	1167	0.000014	0.013
GO:0051704	P	multi-organism process	48	4833	0.000071	0.048
GO:0016798	F	hydrolase activity, acting on glycosyl bonds	17	962	0.000044	0.036
GO:0005576	C	extracellular region	44	2695	2.2E-10	1.2E-07
GO:0009505	C	plant-type cell wall	14	780	0.00018	0.045
GO:0048046	C	apoplast	14	905	0.00024	0.045
PED 12 DAP						
GO term	Ontology	Description	DE AL Genes	Number in BG/Ref	p-value	FDR
GO:0019748	P	secondary metabolic process	31	1706	9.7E-10	5.2E-06
GO:0009628	P	response to abiotic stimulus	55	5674	1.1E-06	0.0019
GO:0006811	P	ion transport	34	2879	3.7E-06	0.005
GO:0009698	P	phenylpropanoid metabolic process	15	761	0.00001	0.0093
GO:0019725	P	cellular homeostasis	17	982	0.000014	0.0093
GO:0048878	P	chemical homeostasis	18	1064	0.00001	0.0093
GO:0006970	P	response to osmotic stress	24	1843	0.000026	0.014

GO:0009651	P	response to salt stress	22	1708	0.000067	0.022
GO:0050801	P	ion homeostasis	14	791	0.000065	0.022
GO:0055082	P	cellular chemical homeostasis	12	589	0.00006	0.022
GO:0055080	P	cation homeostasis	12	622	0.000099	0.028
GO:0006873	P	cellular ion homeostasis	11	539	0.00012	0.031
GO:0009699	P	phenylpropanoid biosynthetic process	10	456	0.00014	0.034
GO:0030003	P	cellular cation homeostasis	10	479	0.0002	0.048
GO:0016491	F	oxidoreductase activity	39	3759	0.000013	0.011
GO:0005886	C	plasma membrane	62	6798	1.4E-06	0.00066
GO:0031976	C	plastid thylakoid	13	778	0.00021	0.02
GO:0042651	C	thylakoid membrane	11	689	0.00091	0.046

Table S7 Expression of transporter genes in the DEGs quantified by the RNA-seq analysis.

Gene ID	Note	log2 (FPKM)
Zm00001d038275	Auxin transporter-like protein 2. amino acid transmembrane transport(GO:0003333)	-4.9468
Zm00001d052063	Vacuolar amino acid transporter 1. amino acid transmembrane transport(GO:0003333)	2.74779
Zm00001d002673	Lysine histidine transporter-like 8. amino acid transmembrane transport(GO:0003333)	3.049
Zm00001d018336	Vacuolar amino acid transporter 1. amino acid transmembrane transport(GO:0003333)	3.34827
Zm00001d049640	Lysine histidine transporter 1. amino acid import(GO:0043090)	3.43567
Zm00001d008285	Probable vacuolar amino acid transporter basic amino acid transmembrane export from vacuole(GO:0034488)	5.2269
Zm00001d037057	Vacuolar amino acid transporter 1. amino acid transmembrane transport(GO:0003333)	6.61336
Zm00001d042438	Proline transporter 1. proline transport(GO:0015824)	9.07794
Zm00001d002176	Lysine histidine transporter 2. amino acid transport(GO:0006865)	11.9544
Zm00001d020208	ABC transporter A family member 8. lipid transport(GO:0006869)	2.3424
Zm00001d022321	Probable sphingolipid transporter lipid transport(GO:0006869)	-20
Zm00001d049252	Bidirectional sugar transporter SWEET14. sucrose transmembrane transporter activity(GO:0008515)	-7.74491
Zm00001d007365	Bidirectional sugar transporter SWEET14. sucrose transport(GO:0015770)	-7.63622
Zm00001d015912	Bidirectional sugar transporter SWEET4. carbohydrate transport(GO:0008643)	-5.47739
Zm00001d021880	CMP-sialic acid transporter 2. sugar:proton symporter activity(GO:0005351)	-4.8796
Zm00001d026156	Putative glycerol-3-phosphate transporter carbohydrate transport(GO:0008643)	-2.8979
Zm00001d021064	Bidirectional sugar transporter SWEET11. copper ion transport(GO:0006825)	-2.86998
Zm00001d015905	Bidirectional sugar transporter SWEET4. carbohydrate transport(GO:0008643)	1.80851
Zm00001d018803	Probable inositol transporter 2. glucose import(GO:0046323)	2.80741
Zm00001d023941	Polyol transporter 5. glucose import(GO:0046323)	3.28383
Zm00001d021936	Polyol transporter 5. glucose import(GO:0046323)	3.3429
Zm00001d029251	Sugar transporter ERD6-like 16. glucose import(GO:0046323)	3.73362
Zm00001d043735	Bidirectional sugar transporter SWEET2b. carbohydrate transport(GO:0008643)	4.6351
Zm00001d044421	Bidirectional sugar transporter SWEET6a. carbohydrate transport(GO:0008643)	7.23092
Zm00001d033053	Molybdate transporter 1. molybdate ion transport(GO:0015689)	-20
Zm00001d025416	Putative magnesium transporter MRS2-D. ion transport(GO:0006811)	-20
Zm00001d052201	Probable magnesium transporter NIPA8. magnesium ion transport(GO:0015693)	-20
Zm00001d003749	Aluminum-activated malate transporter 1. malate transport(GO:0015743)	-8.34836

Zm00001d019327	Metal transporter Nramp5. ion transport(GO:0006811)	-6.96278
Zm00001d025373	Aluminum-activated malate transporter 1. malate transport(GO:0015743)	-6.71769
Zm00001d020395	Vacuolar iron transporter 1.2. cellular manganese ion homeostasis(GO:0030026)	-4.4371
Zm00001d029842	Putative transporter arsB. arsenite transport(GO:0015700)	-3.28468
Zm00001d036784	Potassium transporter 10. potassium ion transmembrane transporter activity(GO:0015079)	-3.2636
Zm00001d037756	Zinc transporter 7. zinc ion transmembrane transporter activity(GO:0005385)	-2.76173
Zm00001d022449	Probable potassium transporter 9. potassium ion transmembrane transporter activity(GO:0015079)	-2.68513
Zm00001d028164	Low affinity sulfate transporter 3. secondary active sulfate transmembrane transporter activity(GO:0008271)	-2.61953
Zm00001d030665	Membrane magnesium transporter. cation transmembrane transport(GO:0098655)	-1.96452
Zm00001d033527	Magnesium transporter MRS2-A, magnesium ion transport(GO:0015693)	2.23388
Zm00001d029497	Zinc transporter 2. zinc II ion transport(GO:0006829)	2.25735
Zm00001d020325	Potassium transporter 23. potassium ion transmembrane transporter activity(GO:0015079)	2.27083
Zm00001d040422	Probable peptide/nitrate transporter regulation of stomatal closure(GO:0090333)	2.80142
Zm00001d049349	Protein NRT1/ PTR FAMILY 4.4. transporter activity(GO:0005215)	3.59611
Zm00001d043614	Sulfate transporter 3.1. sulfate transport(GO:0008272)	3.61784
Zm00001d017069	Inorganic phosphate transporter 2-1, phosphate ion transport(GO:0006817)	3.88505
Zm00001d040002	Metal tolerance protein 7. cation transmembrane transporter activity(GO:0008324)	3.98696
Zm00001d031875	Inorganic phosphate transporter 1-6. phosphate ion transport(GO:0006817)	4.10766
Zm00001d037305	Potassium transporter 24. potassium ion transmembrane transporter activity(GO:0015079)	4.20851
Zm00001d040125	Phosphate transporter PHO1-1. phosphate ion transport(GO:0006817)	4.47095
Zm00001d002269	Potassium transporter 26. potassium ion transmembrane transporter activity(GO:0015079)	7.2735
Zm00001d027700	Inorganic phosphate transporter 1-2. phosphate ion transport(GO:0006817)	8.45414
Zm00001d042244	Potassium transporter 5. potassium ion transmembrane transporter activity(GO:0015079)	10.505
Zm00001d032610	Aquaporin TIP3-2. transporter activity(GO:0005215)	-20
Zm00001d014256	ABC transporter F family member 3. defense response to bacterium(GO:0042742)	-20
Zm00001d035993	Nucleobase-ascorbate transporter LPE1. transmembrane transport(GO:0055085)	-20
Zm00001d048520	Aquaporin TIP3-1. transporter activity(GO:0005215)	-14.9957
Zm00001d015746	Adenine nucleotide transporter BT1, ADP transport(GO:0015866)	-14.4942
Zm00001d033585	Nucleobase-ascorbate transporter LPE1. transmembrane transport(GO:0055085)	-4.80774
Zm00001d017728	Polyamine transporter PUT1. polyamine transport(GO:0015846)	-3.83885
Zm00001d044895	ABC transporter G family member 4. ATP binding(GO:0005524)	-3.54766
Zm00001d026426	ABC transporter B family member 19. acropetal auxin transport(GO:0010541)	-3.16965
Zm00001d011193	Uncharacterized membrane protein C776.05. transmembrane transporter activity(GO:0022857)	-3.10537
Zm00001d028951	Aquaporin SIP2-1. transporter activity(GO:0005215)	-2.86937
Zm00001d032322	Nucleobase-ascorbate transporter 6. transmembrane transport(GO:0055085)	-2.85018
Zm00001d048959	Aquaporin SIP1-1. transporter activity(GO:0005215)	-2.56378
Zm00001d010426	ABC transporter G family member 15. response to karrikin(GO:0080167)	1.85829
Zm00001d016237	Aquaporin NIP1-1. transporter activity(GO:0005215)	1.98829
Zm00001d049905	ABC transporter F family member 1. ATP binding(GO:0005524)	2.0271
Zm00001d008512	ABC transporter G family member 7. ATP binding(GO:0005524)	2.36426
Zm00001d025888	Probable metal-nicotianamine transporter transmembrane transporter activity(GO:0022857)	2.44963
Zm00001d025977	Probable metal-nicotianamine transporter response to iron ion(GO:0010039)	2.51625
Zm00001d003354	ABC transporter G family member 51. ATP binding(GO:0005524)	2.52196

Zm00001d043599	Putative ABC transporter B family member ATP binding(GO:0005524)	2.74904
Zm00001d042953	ABC transporter G family member 16. pollen wall assembly(GO:0010208)	2.85344
Zm00001d009243	ABC transporter C family member 14. transmembrane transport(GO:0055085)	2.99335
Zm00001d002503	ABC transporter C family member 9. response to nematode(GO:0009624)	3.17533
Zm00001d002871	ABC transporter G family member 11. cotyledon vascular tissue pattern formation(GO:0010588)	3.20044
Zm00001d011006	ABC transporter G family member 16. pollen wall assembly(GO:0010208)	3.34003
Zm00001d051514	ABC transporter B family member 2. ATP binding(GO:0005524)	3.39391
Zm00001d005528	ABC transporter B family member 9. ATP binding(GO:0005524)	3.8902
Zm00001d048621	ABC transporter G family member 11. cotyledon vascular tissue pattern formation(GO:0010588)	4.16621
Zm00001d032601	ABC transporter G family member 11. cotyledon vascular tissue pattern formation(GO:0010588)	4.60574
Zm00001d039258	Triose phosphate/phosphate translocator, transporter activity(GO:0005215)	4.61517
Zm00001d038068	ABC transporter G family member 23. ATP binding(GO:0005524)	4.67353
Zm00001d029321	Probable polyamine transporter At3g13620. polyamine transport(GO:0015846)	4.76033
Zm00001d024600	ABC transporter B family member 14. ATP binding(GO:0005524)	4.92556
Zm00001d046226	ABC transporter C family member 8. transmembrane transport(GO:0055085)	5.02071
Zm00001d013960	ABC transporter G family member 11. cotyledon vascular tissue pattern formation(GO:0010588)	5.06899
Zm00001d051872	Aquaporin PIP1-5. transporter activity(GO:0005215)	5.71374
Zm00001d026207	Oligopeptide transporter 4. protein transport(GO:0015031)	5.75899
Zm00001d044476	ABC transporter G family member 10. ATP binding(GO:0005524)	6.90058

REFERENCES

Chen J, Zeng B, Zhang M, Xie S, Wang G, Hauck A, Lai J. 2014. Dynamic transcriptome landscape of maize embryo and endosperm development. *Plant Physiol* **166**, 252-64.

Walley JW, Sartor RC, Shen Z, Schmitz RJ, Wu KJ, Urich MA, Nery JR, Smith LG, Schnable JC, Ecker JR, Briggs SP. 2016. Integration of omic networks in a developmental atlas of maize. *Science* **353**: 814-818.

Zhan J, Thakare D, Ma C, Lloyd A, Nixon NM, Arakaki AM, Burnett WJ, Logan KO, Wang D, Wang X, Drews GN, Yadegari R. 2015. RNA sequencing of laser-capture microdissected compartments of the maize kernel identifies regulatory modules associated with endosperm cell differentiation. *Plant Cell* **27**: 513-531.
